# Lipoprotein X Causes Renal Disease in LCAT Deficiency

**DOI:** 10.1371/journal.pone.0150083

**Published:** 2016-02-26

**Authors:** Alice Ossoli, Edward B. Neufeld, Seth G. Thacker, Boris Vaisman, Milton Pryor, Lita A. Freeman, Christine A. Brantner, Irina Baranova, Nicolás O. Francone, Stephen J. Demosky, Cecilia Vitali, Monica Locatelli, Mauro Abbate, Carlamaria Zoja, Guido Franceschini, Laura Calabresi, Alan T. Remaley

**Affiliations:** 1 Centro Grossi Paoletti, Dipartimento di Scienze Farmacologiche e Biomolecolari, Università degli Studi di Milano, Milano, Italy; 2 Lipoprotein Metabolism Section, Cardiovascular and Pulmonary Branch, National Heart, Lung and Blood Institute, National Institutes of Health, Bethesda, Maryland, United States of America; 3 NHLBI Electron Microscopy Core Facility, National Institutes of Health, Bethesda, Maryland, United States of America; 4 Clinical Center, National Institutes of Health, Bethesda, Maryland, United States of America; 5 IRCCS-Istituto di Ricerche Farmacologiche Mario Negri, Centro Anna Maria Astori, Science and Technology Park Kilometro Rosso, Bergamo, Italy; INSERM, FRANCE

## Abstract

Human familial lecithin:cholesterol acyltransferase (LCAT) deficiency (FLD) is characterized by low HDL, accumulation of an abnormal cholesterol-rich multilamellar particle called lipoprotein-X (LpX) in plasma, and renal disease. The aim of our study was to determine if LpX is nephrotoxic and to gain insight into the pathogenesis of FLD renal disease. We administered a synthetic LpX, nearly identical to endogenous LpX in its physical, chemical and biologic characteristics, to wild-type and *Lcat*^-/-^ mice. Our *in vitro* and *in vivo* studies demonstrated an apoA-I and LCAT-dependent pathway for LpX conversion to HDL-like particles, which likely mediates normal plasma clearance of LpX. Plasma clearance of exogenous LpX was markedly delayed in *Lcat*^-/-^ mice, which have low HDL, but only minimal amounts of endogenous LpX and do not spontaneously develop renal disease. Chronically administered exogenous LpX deposited in all renal glomerular cellular and matrical compartments of *Lcat*^-/-^ mice, and induced proteinuria and nephrotoxic gene changes, as well as all of the hallmarks of FLD renal disease as assessed by histological, TEM, and SEM analyses. Extensive *in vivo* EM studies revealed LpX uptake by macropinocytosis into mouse glomerular endothelial cells, podocytes, and mesangial cells and delivery to lysosomes where it was degraded. Endocytosed LpX appeared to be degraded by both human podocyte and mesangial cell lysosomal PLA_2_ and induced podocyte secretion of pro-inflammatory IL-6 *in vitro* and renal Cxl10 expression in *Lcat*^-/-^ mice. In conclusion, LpX is a nephrotoxic particle that in the absence of Lcat induces all of the histological and functional hallmarks of FLD and hence may serve as a biomarker for monitoring recombinant LCAT therapy. In addition, our studies suggest that LpX-induced loss of endothelial barrier function and release of cytokines by renal glomerular cells likely plays a role in the initiation and progression of FLD nephrosis.

## Introduction

Lecithin:cholesterol acyltransferase (LCAT) deficiency is a rare monogenic disorder caused by loss-of-function mutations in the human *LCAT* gene. LCAT is primarily synthesized by the liver and is secreted into the plasma compartment where it catalyzes the conversion of free cholesterol to cholesteryl esters on HDL and to a lesser degree on LDL [1]. Two different syndromes with different biochemical and clinical features are caused by mutations in the LCAT gene, namely Familial LCAT deficiency (FLD) and Fish-Eye disease (FED) [2]. Homozygous and compound heterozygous carriers of LCAT deficiency have drastic alterations in their lipid/lipoprotein profile, principally characterized by an increased percentage of unesterified cholesterol and by low levels of HDL-C (< 10 mg/dL in FLD, < 27 mg/dL in FED). Heterozygous carriers have an intermediate biochemical phenotype [3]. FLD and FED cases also have other alterations in their lipoprotein distribution, such as the loss of mature spherical HDL and a corresponding increase in small discoidal HDL particles, increased levels of triglycerides and low levels of LDL-C [2,4]. Clinical manifestations of homozygous FLD include corneal opacity, hemolytic anemia and renal disease, whereas FED patients typically have only corneal opacities [2]. Unlike FLD patients, where loss of LCAT activity is observed on both HDL and LDL, mutations that cause FED appear to result in some residual enzyme activity, particularly on LDL [5].

Renal disease is the primary cause of morbidity and mortality in FLD subjects, with proteinuria usually first developing in the teenage years and then progressing to end-stage renal disease (ESRD), typically during the third and fourth decade of life [6–8]. Plasma albumin, serum creatinine, and blood urea nitrogen levels, as well as clearance of creatinine, and inulin may remain normal for years [2]. Many FLD patients have a prolonged history of proteinuria (1–2 g/24 hours) before their BUN and creatinine levels show a substantial increase [5]. The rate of deterioration of kidney function, however, is quite variable and unpredictable and can sometimes rapidly develop in younger individuals. Nephrotic syndrome develops with the onset of renal failure, which can occur rapidly and without warning. FLD patients are often treated by dialysis [9,10] or renal transplant, but the disease can rapidly occur in the transplanted kidneys within only a few years [11].

On renal biopsy, focal segmental glomerular sclerosis is often observed in FLD patients [5]. Other common findings include mesangial expansion, a mild increase in mesangial cellularity, and irregular thickening of the glomerular capillary walls, with vacuolization of the glomerular basement membrane due to intramembranous lipid deposits, resulting in a typical “foamy” appearance [2,5]. Electron microscopy reveals deposition of electron-dense membranes in the capillary lumen, the basement membrane, and the mesangial and pericapsular regions [2]. The capillary walls are abnormal, showing loss of endothelial cells, irregular thickening of the basement membrane, and fused podocyte foot processes [2,5]. Diffuse tubular atrophy with thickening of the tubular basement membranes, along with focal interstitial fibrosis [5]. Mononuclear cells infiltrates can also be found late in FLD [5]. Lipid analysis of isolated glomeruli shows marked increase in the amount of free cholesterol and phospholipids [2].

The cause of the renal disease in FLD is not well understood but has been attributed to the formation of an abnormal lipoprotein particle called lipoprotein-X (LpX), which occurs in FLD but not in FED [3,6] and can also occur with severe cholestasis [12]. Unlike typical lipoproteins, which are micelle-like structures, containing a single layer of surface phospholipids and a hydrophobic core of cholesteryl esters and triglycerides, LpX is a vesicle or a multilamellar vesicle comprised of phospholipid/cholesterol bilayers surrounding an aqueous core. In addition, LpX is enriched in free cholesterol and relatively devoid of hydrophobic core lipids (cholesteryl esters and trigycerides) and apolipoproteins [5]. Unlike typical lipoproteins, LpX migrates toward the cathode during agarose gel electrophoresis [13]. In cell culture studies, LpX was found to be cytotoxic and pro-inflammatory [14]. By in situ perfusion studies, LpX was found to accumulate in the kidney [15] and thus could account for the lipid deposition in mesangial cells, one of the main pathologic findings in the kidney of FLD patients [2,5]. *Lcat*^-/-^ mice have many of the same lipid and lipoprotein abnormalities as FLD patients but do not form substantial amounts of LpX and do not develop significant renal disease [16]. This may be due to the low level of apoB-containing lipoproteins in mice, which may possibly be converted to LpX in the absence of LCAT, during the lipolysis of triglycerides. *Lcat*^-/-^ mice crossed with SREBP1-transgenic mice overproduce VLDL, form LpX, and do spontaneously develop renal disease, but they also contain many other abnormalities in their lipoprotein profile, thus making it difficult to ascertain the cause of their renal disease [17].

Currently, no effective treatment is available for FLD. The effectiveness of dietary interventions, as well as the use of lipid lowering medications and/or ACE inhibitors [18–21] in preventing the development of renal disease in FLD patients is not known. Because LCAT is a relatively low abundant protein and is fairly stable, FLD may be amenable to treatment by enzyme replacement therapy. In fact, recombinant LCAT has been shown to reverse the lipid and lipoprotein abnormalities in *Lcat*^-/-^ mice [22]. Recently, a Phase I clinical trial [23] has shown that recombinant LCAT is safe in patients with stable cardiovascular disease and moreover, the lipid and lipoprotein abnormalities of one patient with FLD was reported to be corrected by treatment with recombinant LCAT [24]. Further development of recombinant LCAT for the treatment of FLD will likely depend on the use of biomarkers, because of the rarity of the disease and the long time period that is necessary for renal disease to develop. Monitoring the effect of recombinant LCAT on LpX levels could provide an early indicator of the effectiveness of the therapy, but it still is not known whether LpX is causally involved or simply associated with the renal disease.

In the present study, we examined the possible role of LpX in renal injury by intravenously administering exogenous LpX into *Lcat*^-/-^ mice. We show that the chronic administration of LpX into these mice results in the accumulation of LpX in the kidney and recapitulates most of the renal findings of FLD. Furthermore, we show that LpX administration induces the expression of genes associated with nephrotoxicity and leads to proteinuria, thus demonstrating for the first time a direct causal role of LpX in the pathogenesis of renal disease.

## Materials and Methods

### Formation of Synthetic LpX

Multilamellar LpX particles containing 24 mole % cholesterol were formed by combining 24.4 mg L-α-lecithin (32 μmoles) together with 4.25 mg cholesterol (10 μmoles) from their respective stock solutions in chloroform. For in vivo studies, fluorescent synthetic LpX particles included the addition of 171 μg (130 nmoles) fluorescent-tagged PE (1,2-dioleoyl-*sn*-glycero-3-phosphoethanolamine–N-(lissamine rhodamine B sulfonyl)). For *in vitro* studies, 74 μg (128 nmoles) fluorescent TopFluor cholesterol (23-(dipyrrometheneboron difluoride)-24-norcholesterol) in chloroform was also added. The lipid mixtures were dried under nitrogen. All lipids were obtained from Avanti Polar Lipids, Inc. Two ml of saline or PBS were added to the dried lipids for preparations to be used *in vivo* and *in vitro*, respectively. The lipids in buffer were vortexed for 10 min to resuspend the dried lipids and then sonicated for 10 min using 1 min bursts separated by a 15 sec rest interval to generate multilamellar particles. The cholesterol and phospholipid composition of the synthetic LpX particles was confirmed using enzymatic colorimetric assays, as previously described [16].

### *In Vitro* Studies of LpX Particle Remodeling

Dual-labeled synthetic fluorescent LpX was made as described above. Human apoA-I isolated as previously described [25] was labeled with Alexa-647 as per the manufacturer’s instructions (Invitrogen). Dual-labeled synthetic fluorescent LpX (20 μl containing 288 μg total lipid) was incubated overnight at 37°C with de-fatted BSA (DF-BSA) (20 μl containing 160 μg), apoA-I (6 μl containing 6 μg) and 20, 40, 60 mU recombinant LCAT (MedImmune Corporation) with sufficient PBS for a total reaction mixture volume of 70 μl. Control reactions included LpX incubated with fluorescent LpX and DF-BSA with either fluorescent apoA-I alone or recombinant LCAT alone. LpX remodeling was monitored by electophoresis of 10 μl of the reaction mixture using Sebia agarose gels. Fluorescent spots on the gel were monitored using a Typhoon 9400 Variable Mode Imager (GE). Fluorescent PE, fluorescent cholesterol and, fluorescent apoA-I were scanned and fluorescence detected using excitation/emission wavelengths of 532/560 nm, 488/520nm and, 633/670 nm, respectively. Following imaging of fluorescent lipids and protein, gels were stained with Sudan Black and scanned.

### Animals

Mice were housed under controlled conditions, with a 12/12 h light/dark cycle with free access to food and water and under pathogen–free conditions. Mice were fed a standard rodent autoclaved diet (NIH31 chow diet; Zeigler Brothers Inc., Gardners, PA). *Lcat* ^-/-^ mice with a knockdown *Lcat* gene, which had been transferred through eight backcrosses to the C57BL/6 mouse stain, as previously described [16], were used in these studies. Experiments were performed on 8–12 month old *Lcat* ^*-/-*^ females. Sex and age matched control C57Bl/6N mice were from Taconic. This study was carried out in strict accordance with the recommendations in the Guide for the Care and Use of Laboratory Animals of the National Institutes of Health. The protocol was approved by the National Heart, Lung and Blood Institute Institutional Animal Care and Use Committee, NIH, Bethesda, MD, United States (NIH Protocols H-0050 and H-00100). All surgery was performed under Avertin anesthesia. When required, mice were euthanized by CO_2_ exposure from a compressed source (e.g., cylinder or house supply) into a closed chamber followed by cervical dislocation. Cervical dislocation alone was only performed by staff that had been certified as proficient in this technique. All efforts were made to minimize suffering.

### Chronic *In Vivo* Administration of Fluorescent LpX

Control wild type (C57BL/6; Taconic Biosciences) and *Lcat*^-/-^ mice [22] were injected intra-orbitally with either 0.5 mg or 1 mg of filter-sterilized non-fluorescently tagged synthetic LpX suspended in 200 μl saline three times per week for five weeks for low (n = 8) and high dose (n = 5) studies, respectively. For the high dose study, mice were then injected with 1 mg of filter-sterilized fluorescent PE-tagged LpX suspended in 200 μl saline 4 hrs prior to euthanasia and harvesting of tissues. This time point was determined by pilot studies that demonstrated nearly complete plasma clearance of fluorescent PE-tagged LpX in wild type mice. Immediately after being euthanized, in the low dose studies, one half of the right kidney was collected in RNAlater (Invitrogen) for gene expression analysis and the other half collected for histological analysis; in the high dose studies, left kidney was bisected and one half was immersed in EM fixative (see below) while the other half was immersed in OCT and frozen on dry ice.

### Plasma Clearance of Synthetic Fluorescent PE-tagged LpX

Blood samples were obtained at 0 (pre-bleed), 5, 10, 20, 60, 120, 240, and 360 min following intra-orbital injection of 1 mg fluorescent PE-tagged LpX into WT and *Lcat*^-/-^ mice. Pooled serum (10 μl) at each time point was diluted with 240 μl and rhodamine fluorescence in plates was measured using 540/600 excitation/emission filters using a Perkin Elmer Victor^3^ 1420 Multichannel Counter. Pooled serum samples from WT and *Lcat*^-/-^ mice were analyzed by FPLC as described below.

### LpX Uptake of Fluorescent PE-tagged LpX by Mouse Renal Glomeruli *In Vivo*

Frozen kidney sections (16 μm) from WT or *Lcat*^-/-^ mice chronically treated with LpX, and then given a bolus of fluorescent PE-tagged LpX (as described above), were mounted on microscope slides, fixed with 4% paraformaldehyde for 10 min at room temperature, washed 3x with PBS and coverslipped. These preparations were immediately imaged though the entire thickness with a Zeiss 780 LSM using a 40x Plan-Apochromatic oil lens. LpX lissamine rhodamine-PE fluorescence was imaged using excitation/emmision wavelengths of 541 nm/570-695 nm, respectively. No detectable signal was observed in sections of fixed frozen kidney sections from control mice that did not receive LpX. Image stacks were rendered as maximum projection images using Zeiss Zen software.

### Electron Microscopic Analyses

Synthetic LpX particles: fluorescent PE-tagged synthetic LpX formed as described above was mixed with 10% liquefied low-melting point agarose and then solidified on ice. The embedded LpX was then immediately fixed in 2.5% glutaraldehyde /1% paraformaldehyde in 0.12 M sodium cacodylate buffer (pH 7.4) at 4°C overnight. Fixed samples were washed in cacodylate buffer, postfixed in 1% OsO4 in cacodylate buffer, washed, stained en bloc with uranyl acetate, ethanol dehydrated, and EMbed-812 embedded (Electron Microscopy Sciences, Hatfield PA). Thin sections were stained with uranyl acetate and lead citrate prior to imaging with a JEM1400 electron microscope (JEOL USA) equipped with an AMT XR-111 digital camera (Advanced Microscopy Techniques Corp). Vesicle diameters were measured using Image J software. Synthetic LpX particle distribution in mouse renal glomeruli: For TEM analysis, mouse kidney cortex was cut into 1 mm^3^ pieces in 2.5% glutaraldehyde, 1% paraformaldehyde, 0.12M sodium cacodylate buffer, pH 7.4 at room temperature and then placed in fresh fixative overnight and then processed, stained, and imaged as above. For SEM analysis, kidney cortical pieces were fixed washed and post-fixed with 1% OsO4 in cacodylate buffer and washed in buffer as above. The tissue was stained *en bloc* with 1% uranyl acetate for one hour and serially dehydrated in ethanol. The samples were critical point dried (Samdri-795, Tousimus, Rockville, MD), placed on carbon adhesive tape coated with 10nm gold in an EMS 575X sputter coater (Electron Microscopy Sciences, Hatfield, PA). The images were obtained in a Hitachi S3400-N1 SEM (Hitachi High Technologies, Pleasanton, CA).

### Renal Histology

Kidney samples were fixed in Duboscq-Brazil, dehydrated and embedded in paraffin. Three-micrometer sections were stained with periodic acid-Schiff (PAS) reagent, and at least 50 glomeruli were examined for each animal. The degree of glomerular matrix expansion was quantified using a score from 0 to 3 (0 = no mesangial matrix expansion; 1 = minimal; 2 = moderate; 3 = diffuse mesangial matrix expansion). Biopsies were analyzed by the same pathologist who was unaware of experimental groups.

### FPLC Analysis

For *in vivo* studies, 250 μl of pooled serum from five WT or *Lcat*^-/-^ mice sampled at various times after injection of LpX, or 40 μl of reaction mixture in *in vitro* studies, were diluted with 360 μl PBS and applied to Separose 6 10/300 GL columns on an AKTA FPLC (Amersham Biosciences) and 0.5 ml fractions were collected.

### Renal Function Assay

Urine samples were collected over 24 hr prior to and then at the end of each week of treatment, using metabolic cages. Albumin content in urine was measured with commercial indirect competitive ELISA (Exocell) while creatinine content was determined by colorimetric assay (Exocell).

### Gene Expression Analysis

After chronic treatment of *Lcat*^-/-^ mice with LpX or saline, one half of the kidney was used for gene expression analysis, as previously described [26]. Isolated RNA had an A260:A230 ratio greater than 1.7; an A260:A280 ratio of approximately 2.1 ± 0.1; and a RIN number of 8.2 ± 0.2. The effects of LpX treatment on gene expression in kidneys was analyzed with the Mouse Nephrotoxicity RT^2^ Profiler PCR Array (Qiagen, catalog No. PAMM-094ZE) following the manufacturer instructions. ABI 7900HT Real-Time PCR System with 384-well block was used. The array profiles the expression of 84 key genes implicated as potential biomarkers of kidney toxicity. Relative expression of the genes was calculated by the comparative C_**T**_ (ΔΔCT) method [27], using software provided by manufacturer of the Array and the REST 2009 software from Qiagen.

### Cell Culture

Mouse mesangial cells (SV40 MES 13) were obtained from ATCC and cultured as recommended. Briefly, 2:1 DMEM:F12 media was supplemented with 5% FBS and media was changed every 2 to 3 days. Human podocytes transfected with the temperature-sensitive SV40-T gene (a kind gift from Dr. Jeffery Kopp) [28] were maintained at the permissive temperature of 33°C. Ten days prior to experiments with podocytes, cells were transferred to 37°C to allow differentiation into mature podocytes. Cells were maintained in RPMI + 10% FBS with media changed every 2–3 days. HUVEC cells (ATCC^®^ CRL-1730^™)^ were maintained in EndoGRO-VEGF (Millipore) and the medium was changed every 2-3d. Mouse mesangial cells grown to confluence in 24 multi-well plates were supplemented with or without 200 μg LpX/ml. After 18 hr of treatment, supernatants were harvested. The IL-6 content in the supernatants was quantified using ELISA (Biolegend) following the manufacturer’s recommendations. Phalloidin staining (Invitrogen) was performed according to manufacturer’s instructions.

### HUVEC Cell Monolayer Impedance Measurements

HUVEC cells (ATCC^®^ CRL-1730^™^) were cultured using EndoGRO-LS Complete Media Kit (Millipore, cat # SCME001) in the presence of 10% FBS. Cells were seeded at 40,000 per well on ACEA RT-CES 16-well E-plates, allowed to sediment at room temperature for 30 min and then placed into the ACEA RT-CES instrument (ACEA Biosciences) for impedance monitoring. Following a 20–24 hr equilibration period to allow cells to form a confluent monolayer, the plate was removed and lipoproteins [LDL, HDL (1 mg/ml) or LpX (5 mg/ml)] were added. The plate was returned to the CO_2_ incubator, connections with the instrument were re-established and impedance monitoring was continued for the next 2 hrs. Responses were reported as Cell Index, a parameter derived from the impedance measurements.

### Fluorescent PE-tagged LpX Uptake by Podocytes and Mesangial Cells *In Vitro*

Mesangial and podocyte cells were plated on chambered coverglass slides and allowed to attach overnight, then treated for 18 hours with 200 μg/ml fluorescent-PE-labeled LpX in the absence or presence of 5 μM amiodarone (Sigma). Cells were then washed and stained with Lysotracker green (Invitrogen) for 2 hrs, in the absence or presence of 5 μM amiodarone and washed 4x times with Ringers buffer. Cells were imaged using 20x or 63x objectives on a Zeiss 780 confocal microscope. Imaging parameters for LpX-lissamine-rhodamine fluorescence were optimized using amiodarone-treated, LpX-labeled cells, which had relatively intense fluorescence. Non-treated cells were then imaged using the same imaging conditions to allow for comparison of LpX between control and treated cells under non-saturating imaging conditions.

## Results

### Formation of Synthetic LpX Particles

**Fig 1:**
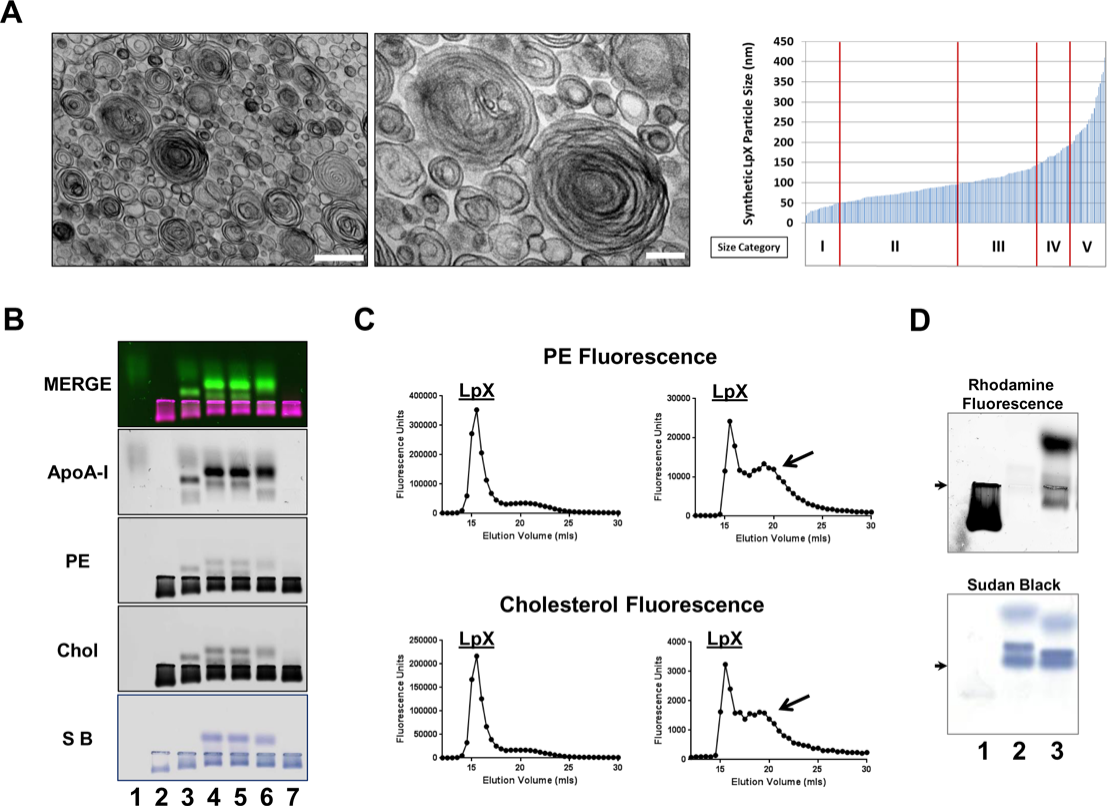
LpX remodeling *in vitro.* (A) TEM analysis of synthetic LpX particles. *Left panel*: Low magnification image (Scale bar; 500 nm*)*. *Middle panel*: High magnification (Scale bar; 100 nm). *Right panel*: LpX particle size distribution. Size categories (nm): I (0–50), II (50–100), III (100–150), IV (150–200), and V (200–245). Small unilamellar vesicles as well as small, medium and, large multivesicular vesicles are seen. (B) LpX remodeling by LCAT and apoA-I in vitro. Agarose gel electrophoresis of LpX labeled with both fluorescent PE *(red)* and cholesterol (*blue*) incubated with Alexa 647-tagged apoA-I (*green*) and/or LCAT in vitro and scanned. Colocalization of LpX PE and cholesterol fluorescence is seen as *magenta* (merged image). Lane 1: ApoA-I; Lane 2: LpX; Lane 3: LpX + ApoA-I; Lanes 4–6: LpX + ApoA-I + 2, 4, or, 6 mg LCAT, respectively; Lane 7: LpX + 6 mg LCAT. (C) FPLC analysis of dual fluorescent PE- and cholesterol-tagged LpX incubated without (*left*) or with apoA-I and 6 mg LCAT *(right*). Fractions were analyzed for rhodamine (PE) fluorescence (*upper panels*) and TopFluor cholesterol fluorescence (*lower panels*). Note the additional peak (*arrows*) after incubation with apoA-I and LCAT. (D) LpX is converted to plasma HDL in vitro. Fluorescent PE-tagged LpX was incubated overnight with pooled human plasma. Agarose gels were scanned for PE fluorescence and then stained with Sudan Black. Lane 1: Fluorescent LpX. Lane 2: Pooled human plasma. Lane 3: Pooled human plasma + fluorescent LpX. *Arrows* indicate origin.

In order to produce a consistent and well characterized source of LpX, we developed a procedure, using cosonication (see Methods) to produce synthetic multilamellar vesicles with a lipid composition and physical characteristics similar to LpX found in FLD patients [5]. The synthetic LpX contained 75% phosphatidylcholine and 25% free cholesterol (% mole fraction), similar to the lipid composition of LpX described in FLD patients [5]. Also similar to LpX isolated from FLD patients, synthetic LpX was found by transmission EM (Fig 1A), to be a heterogeneous population of vesicles (diameter range: 19–450 nm) that included small, medium, and large multilamellar particles (50–400 nm), as well as small unilamellar vesicles (< 50 nm). Vesicles assigned to size categories I (0–50 nm), II (50–100 nm), III (100-150nm), IV (150–200 nm), and V (200–245 nm) had a median size (% total) of 38 nm (11%), 74 nm (42%), 115 nm (25%), 172 nm (11%), and 257 nm (11%), respectively (Fig 1A). To fluorescently tag the synthetic LpX, trace amounts of fluorescent lipids were added during the cosonication procedure.

### ApoA-I Removes Lipids from LpX to Form a Particle that is Remodeled by LCAT

It has been previously shown that endogenous LpX formed by cholestatic patients undergoes a shift in its electrophoretic migration towards the anode once it binds apoA-I and can undergo further remodeling to a smaller and faster migrating particle when incubated with apoA-I and LCAT [13]. In Fig 1B, we tested whether our synthetic LpX would behave similarly. For these studies, LpX was labeled with both a non-exchangeable lipid, fluorescent PE and with fluorescent cholesterol (TopFluor-cholesterol). ApoA-I was tagged with Alexa 647. Fluorescent apoA-I ran as a diffuse band near the top of the gel toward the anode (Lane 1). In contrast, the synthetic LpX (Lane 2) showed cathodal migration like endogenously produced LpX. In the presence of apoA-I, the overall amount of LpX was reduced, and, a new anodal migrating particle appeared that bound apoA-I and possessed both LpX-derived fluorescent PE and cholesterol (Lane 3). This new apoA-I containing particle generated from LpX did not stain with the neutral lipid stain Sudan black, suggesting the absence of cholesteryl esters in the particle. In the presence of LCAT, apoA-I promoted the formation of an even faster anodal migrating particle that contained both LpX-derived fluorescent PE and cholesterol and stained with Sudan black, consistent with LCAT-mediated esterification of LpX-derived cholesterol (Lanes 4, 5, 6). LpX incubated with LCAT alone did not form a new particle (lane 7), confirming that LCAT-mediated esterification of LpX cholesterol is apoA-I-dependent. FPLC analysis (Fig 1C) confirmed the formation of LpX-derived particles (containing both fluorescent PE and cholesterol) by LCAT in the presence of apoA-I. These particles eluted between 17.5–25 ml with a peak at 20 ml at the same location as isolated HDL. To further explore the role of LCAT in LpX remodeling, we incubated fluorescent PE-tagged LpX with pooled normal human plasma *in vitro*. As shown in Fig 1D, the LpX particle was almost completely remodeled and the bulk of fluorescent PE–tagged LpX associated with HDL, consistent with LCAT-mediated conversion of LpX into a HDL-like particle. Taken together, these findings establish that remodeling of fluorescent synthetic LpX can occur by a process that is dependent upon both apoA-I and LCAT.

### Plasma Clearance of LpX is Delayed in *Lcat*^-/-^ Mice

**Fig 2:**
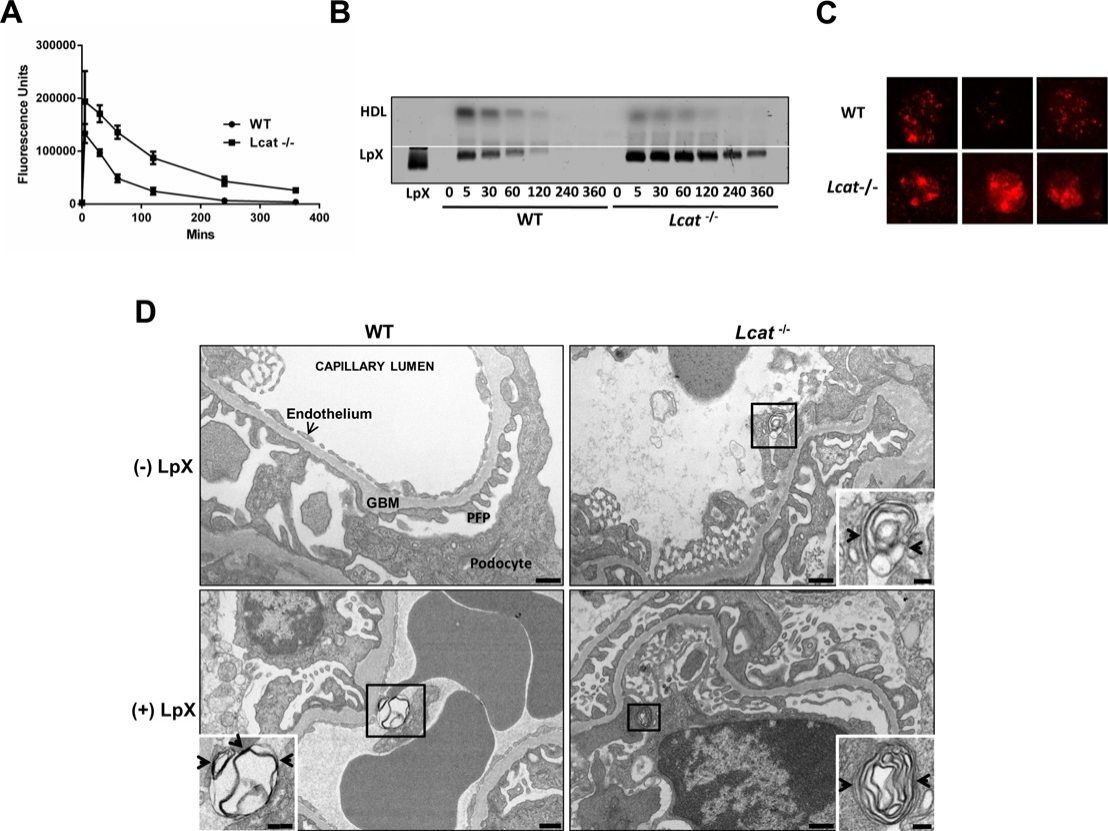
LpX plasma clearance and glomerular upake *in vivo.* LCAT deficiency markedly decreases LpX plasma clearance. WT and *Lcat*^-/-^ mice were injected with lissamine rhodamine B PE-tagged LpX and plasma samples were taken at the indicated times. (A) Plasma-associated fluorescence. Each data point represents the total fluorescence of pooled mouse plasma samples (mean ± S.D.; n = 3). (B) Agarose gel electrophoresis of pooled mouse plasma lipoprotein PE fluorescence (same samples as in (A)). LpX cleared from WT plasma by 240 min, whereas *Lcat*^-/-^ LpX remained elevated at all times. HDL-associated fluorescence was increased in WT plasma. W*hite line* indicates origin. (C) Fluorescent LpX retention in renal glomeruli is markedly increased in *Lcat*^-/-^ mice. Representative confocal maximum projection images of 10 μm fixed frozen kidney sections 4 hrs after injection of fluorescent-PE tagged LpX in mice chronically treated with 3 mg/wk synthetic LpX. Note the markedly increased retention of LpX in *Lcat*^-/-^ mice glomeruli. (D) Electron microscopic analysis of LpX in renal glomerular capillaries. Representative TEM of renal glomerular capillaries in WT (*left panels*) and *Lcat*^-/-^ mice (*right panels*). Endogenous multilamellar structures with features of LpX particles were occasionally present in the capillaries of (-) LpX *Lcat*^-/-^, but not (-) LpX WT mice. Synthetic LpX particles resembling endogenous LpX were frequently observed in renal capillaries of both (+) LpX WT and (+) LpX *Lcat*^-/-^ mice. Both endogenous and exogenous synthetic LpX were often seen to be engulfed by endothelial cell processes (*insets*). Exogenous LpX in the capillary lumen bound to red blood cells in LpX-treated WT and *Lcat*^-/-^ mice. GBM: Glomerular Basement Membrane; PFP: Podocyte Foot Process. Scale bars = 500nm. Inset scale bars = 250 nm (WT+LpX); 100 nm (*Lcat*^-/-^ ± LpX).

To assess the relative rates of LpX plasma clearance in WT and *Lcat*^-/-^ mice, fluorescent PE-tagged synthetic LpX was intravenously administered in mice and PE fluorescence in mouse plasma was monitored. As shown in Fig 2A and 2B, LpX-associated fluorescent PE rapidly cleared from the plasma of WT mice between 5 and 120 min and was nearly undetectable within 4 hrs. Approximately half of the LpX-derived fluorescent PE was associated with HDL throughout the time course in WT mice. In contrast, plasma clearance of LpX was markedly delayed in *Lcat*^-/-^ mice and LpX levels remained elevated up to 6 hrs post-injection. Plasma LpX in *Lcat*^-/-^ mice was elevated approximately 2-4-fold relative to WT levels at all times up to 6 hrs.

### LpX Uptake by Renal Glomeruli is Markedly Increased in *Lcat*^-/-^ Mice

To assess the effect of chronic administration of LpX, WT and *Lcat*^-/-^ mice were injected with 1 mg of synthetic LpX three days a week for 4 wks and then for the last injection, 1 mg of fluorescent PE-tagged synthetic LpX 4 hrs prior to plasma and tissue collection. Persistently elevated levels of LpX were observed 4 hrs post-injection in the *Lcat*^-/-^ mice, but no LpX was detected in WT plasma by filipin staining of free cholesterol or by monitoring PE fluorescence (S1A Fig), consistent with the results obtained with a single injection of fluorescent LpX (Fig 2A and 2B). In both WT and *Lcat*^-/-^ mice fluorescent PE-tagged LpX was observed by confocal microscopy to accumulate in renal glomeruli, but the *Lcat*^-/-^ mice had markedly increased renal glomerular deposition. (Fig 2C; S2 Fig). Moreover, retention of LpX was more widespread throughout the *Lcat*^-/-^ mouse glomeruli, suggesting the possibility that LpX was retained in the mesangium. Fluorescent tagged LpX was also observed in the tubular cells of *Lcat*^-/-^ but not WT mice (S2 Fig). Taken together, these findings suggest that the sustained plasma levels of LpX observed in *Lcat*^-/-^ mice leads to greater renal deposition.

We next conducted extensive electron microscopic studies to better characterize the distribution of exogenous LpX in WT and *Lcat*^-/-^ mouse renal glomeruli. Renal glomerular endothelium, glomerular basement membrane (GBM), podocytes, and podocyte foot processes (PFPs) appeared to be normal in control WT mice (Fig 2D; WT (-) LpX). In *Lcat*^-/-^ mice, endogenously produced LpX particles were occasionally observed in the capillary lumen, typically bound to the surface of endothelial cells (Fig 2D; *Lcat*^-/-^ (-) LpX; *inset*). After chronic treatment with exogenous synthetic LpX, multilamellar LpX particles were readily observed in the lumen of renal capillaries, particularly in *Lcat*^-/-^ mice, and were typically bound to the surface of endothelial cells (ECs; Fig 2D: (+) LpX; S3 Fig, S4 Fig) The synthetic multilamellar LpX particles in WT ((+) LpX) and *Lcat*^-/-^ mice ((+) LpX) were remarkably similar in structure to endogenously produced LpX in *Lcat*^-/-^ mice (Fig 2D). LpX was also frequently observed to be bound to the surface of red blood cells (RBCs; S3M Fig) or bound to both RBCs and the endothelial surface (Fig 2D WT (+) LpX; S4A Fig), which could account for the 28-fold increase in RBC-associated LPX–fluorescent PE found in *Lcat*^-/-^ mice compared to WT mice (S1B Fig). Splenic clearance of RBCs with an abnormal lipid composition due to their interaction with LpX provides a potential mechanism for the anemia that occurs in FLD patients [2].

### LpX is Internalized by Glomerular Capillary Endothelial Cells by Macropinocytosis

**Fig 3:**
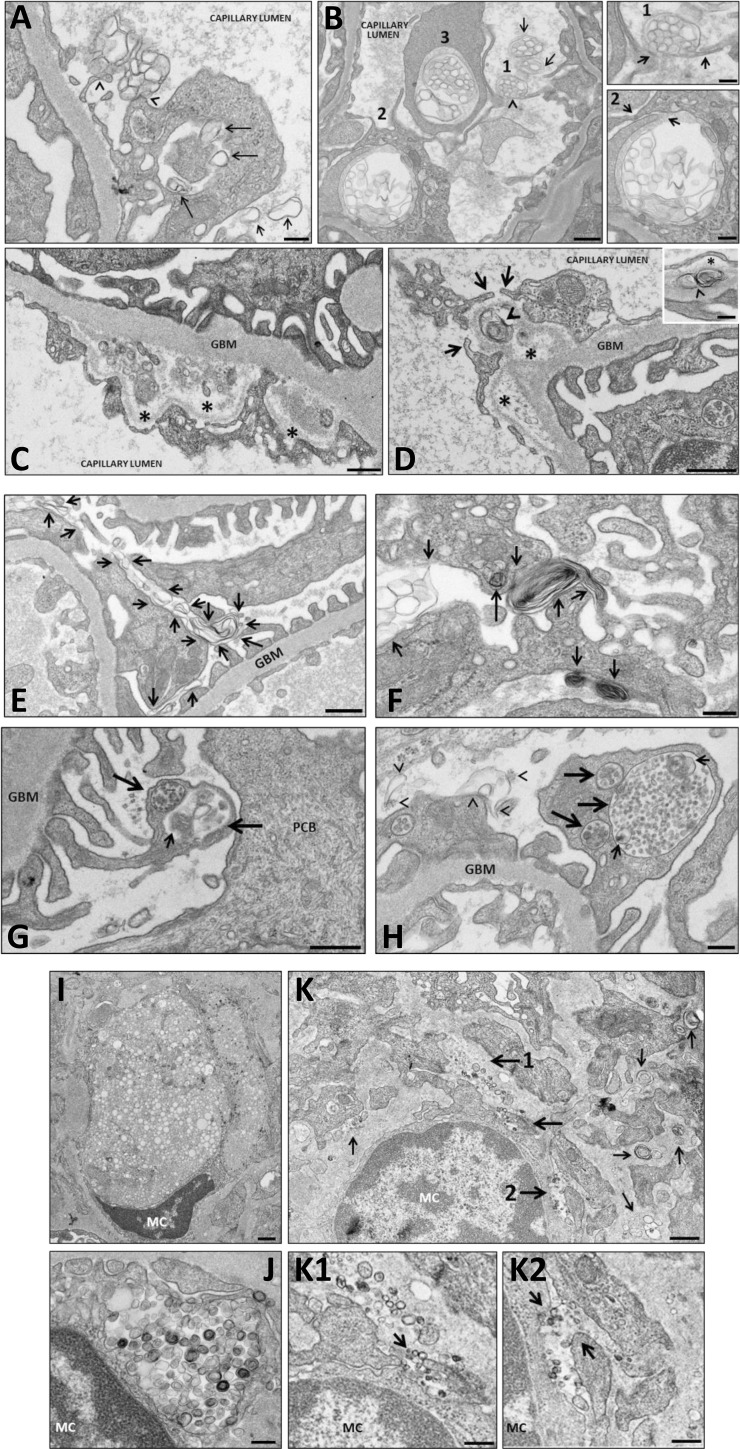
Electron microscopic analysis of LpX movement through renal glomerular compartments. Circulating LpX particles (small arrows in (A, B)) bind to endothelial cell lamellipodia in (A) WT and (B) *Lcat*^-/-^ mouse glomerular capillaries (arrowheads), are internalized (long arrows in (A), and degraded (see also S3 Fig, S4 Fig). LpX bound to the cell surface (B1), is partially (B2), small arrows in inset) and then completely engulfed (B3). LpX penetrates the glomerular basement membrane (GBM) in WT (C) and *Lcat*^-/-^ mice ((D) and, inset in (D), arrowheads), markedly disrupting its structure (C, D; asterisks). The typical intramembranous lesion as found in the peripheral GBM of human FLD is seen in the inset in D, displaying a characteristic lamellar structure within a lucent lacuna in *Lcat*^-/-^ mice. In (D), several lamellipodia (arrows) engulf an LpX particle in the GBM. LpX penetrates the glomerular urinary space of both WT (E, G) and *Lcat*^-/-^ (F, H) mice. LpX binds to podocyte cell bodies (PCBs) and foot processes (PFPs) at multiple sites (E, F: small arrows; H: arrowheads), and was internalized into PCBs (F; large arrow). Large vacuoles (G, H; large arrows) containing partially degraded LpX particles (G, H; small arrows) as well as numerous small unilamellar vesicles are often observed, consistent with cell-mediated LpX degradation. (I) In WT mice, LpX did not accumulate in the mesangial matrix and occasional foamy mesangial cells were observed. (J) Mesangial cells near the sites of LpX deposition engulf LpX particles. (K) Marked retention of LpX in *Lcat*^-/-^ mouse mesangial matrix. The regions near large arrows 1 & 2 in (K) are shown enlarged in K1&2. LpX binds to the mesangial cell prior to engulfment. Scale bars: A, B1, F, H, J = 200 nm; B2, D (inset), K1, K2 = 250 nm; B–E, G, I, K = 500 nm. See S3 Fig and S4 Fig, for additional examples.

By transmission electron microscopy (TEM), we found the following evidence for the macropinocytotic uptake of LpX by capillary ECs: (i) initial binding of LpX to the surface of ECs (Fig 3A; S3A Fig; S4A and S4C Fig) or to EC lamellipodia (Fig 3A and 3B; S3B and S3D Fig; S4B, S4C, S4D and S4J Fig), with concomitant plasma membrane ruffling, followed by (ii) cellular entrapment of LpX particles via extension of EC lamellipodia (Fig 3B; S3C Fig; S4E, S4F, S4H and S4I Fig) and, then; (iii) contact and fusion of lamellipodia with the cell body, resulting in the formation of large intracellular vesicles containing multilamellar LpX (Fig 3B; S3D, S3E and S3F Fig; S4D, S4G–S4I and S4K Fig). Large intracellular vesicles were also observed that contained both intact and partially degraded LpX particles, suggesting that LpX particles were degraded in these intracellular vesicles (Fig 3A; S3C, S3D and S3F Fig; S4E and S4J Fig). Many ECs contained small intracellular unilamellar vesicles that likely represent partially degraded multilamellar LpX particles (S3G–S3K Fig; S4J, S4L and S4M Fig).

### LpX Penetrates and Disrupts the Glomerular Basement Membrane

Administered LpX particles entered the GBM in both WT (Fig 3C; S4L and S4N Fig) and *Lcat*^-/-^ mice (Fig 3D; S4N–S4T Fig). Regions of the GBM in which LpX particles were embedded were often enlarged and, moreover, the embedded LpX particles disrupted the structural integrity of the GBM and, in many cases, led to dissolution of the GBM matrix. As seen in Fig 3D, LpX particles retained in the disrupted GBM appear to have activated capillary EC macropinocytosis. The most typical intramembranous lesion, as found in the peripheral GBM in biopsy samples of patients with familial LCAT deficiency, was detected in *Lcat*^-/-^ mice treated with LpX (Fig 3D, inset), showing a characteristic lamellar structure within a lucent lacuna.

### LpX is Internalized by Glomerular Podocytes by Macropinocytosis

A considerable number of LpX particles from mice treated with exogenous LpX were observed to enter the glomerular urinary space in WT and *Lcat*^-/-^ mice. Podocyte cell bodies (PCB) and podocyte foot processes (PFP) appeared to macropinocytose LpX particles by a sequential process similar to that observed in capillary ECs. Initially, LpX particles bind to PCBs and PFPs (Fig 3E and 3F; S5A–S5K Fig; S6A–S6N Fig). LpX particles were often seen bound to (i) single PCBs or PFPs; (ii) multiple PCBs or multiple PFPs; or, (iii) both PCBs and PFPs at multiple sites. PFPs were occasionally observed to contain extremely large vacuoles (Fig 3G and 3H; S5O–S5R Fig; S6S–S6V Fig) containing partially degraded LpX, as well as a number of unilamellar vesicles. In addition, both PCBs and PFPs often contained numerous small vacuoles (multivesicular bodies (MVBs) containing small unilamellar vesicles. PCBs containing empty vacuoles were also occasionally seen, likely representing vacuoles in which LpX particles were completely degraded.

### LpX is Internalized by Glomerular Mesangial Cells and Accumulates in Mesangial Extracellular Matrix in *Lcat*^-/-^ Mice

In WT mice, occasional foamy mesangial cells (MCs) were sometimes observed following chronic LpX administration (Fig 3I; S7A and S7B Fig). In marked contrast, extensive MC–mediated macropinocytosis of LpX particles was readily observed in *Lcat*^-/-^ mice (Fig 3J; S8A–S8J Fig), as well as massive deposition of LpX in mesangial matrix (Fig 3K, 3K1 and 3K2; S8A–S8D, S8G–S8P Fig).

### Chronic Administration of LpX Increases Proteinuria in *Lcat*^-/-^ but not WT mice

**Fig 4:**
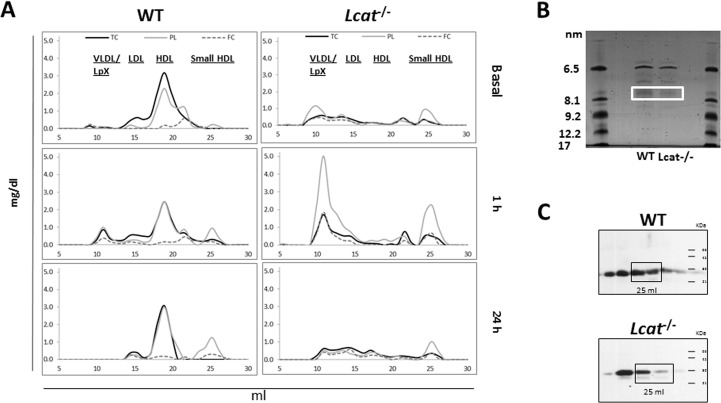
LpX metabolism *in vivo.* (A) Blood samples from *Lcat*^-/-^ and WT mice were collected prior to (“basal”) and, at 1 and 24 hrs after LpX injection. Plasma samples from WT (n = 6) and *Lcat*^-/-^ (n = 6) mice were pooled and lipoproteins were separated by FPLC. Phospholipid (PL), Total Cholesterol (TC), and Free Cholesterol (FC) were measured in collected fractions. Prior to LpX injection, TC, PL and FC were abundant in HDL in WT mice, whereas they were absent in *Lcat*^-/-^ mice, which have only small amounts of lipids in VLDL/LpX and small HDL. One hour after injection in *Lcat*^-/-^ mice, LpX PL and FC are clearly present in a large peak in the VLDL region, whereas in WT mice, the peak is reduced, consistent with our findings using fluorescent PE-tagged LpX (Fig 1B). One hour after LpX administration, a new peak in the HDL region (25 ml elution volume) appeared in WT mice; in *Lcat*^-/-^ mice, this peak was observed prior to LpX administration and was increased at 1 hr post-injection. At this time, the PL and FC content of the *Lcat*^-/-^ peak was increased compared to the *Lcat*^-/-^ pre-injection peak, as well as to the WT peak. (B) Characterization of particles eluted at 25 ml using native gradient gel electrophoresis 1 hr post-injection. Native gradient gel electrophoresis confirmed that lipid-containing particles were present in the 25 ml fraction in the 7–8 nm size range. (C) SDS-PAGE (16% acrylamide gel) apoA-I immunoblot of small HDL particles (25 ml elution volume) generated by LpX at I hr. ApoA-I immunostaining confirmed the presence of apoA-I in these particles, which suggests that in the presence of apoA-I, LpX-derived PL, and to a lesser extent FC, increased the pool size of small HDL particles. These findings *in vivo* are consistent with the apoA-I and LCAT-dependent remodeling of LpX that we observed in vitro (Fig 1B–1D). The peak is still visible 24 hours after injection in WT mice, while in *Lcat*^-/-^ mice, it returns to basal levels (Fig 4A).

**Fig 5:**
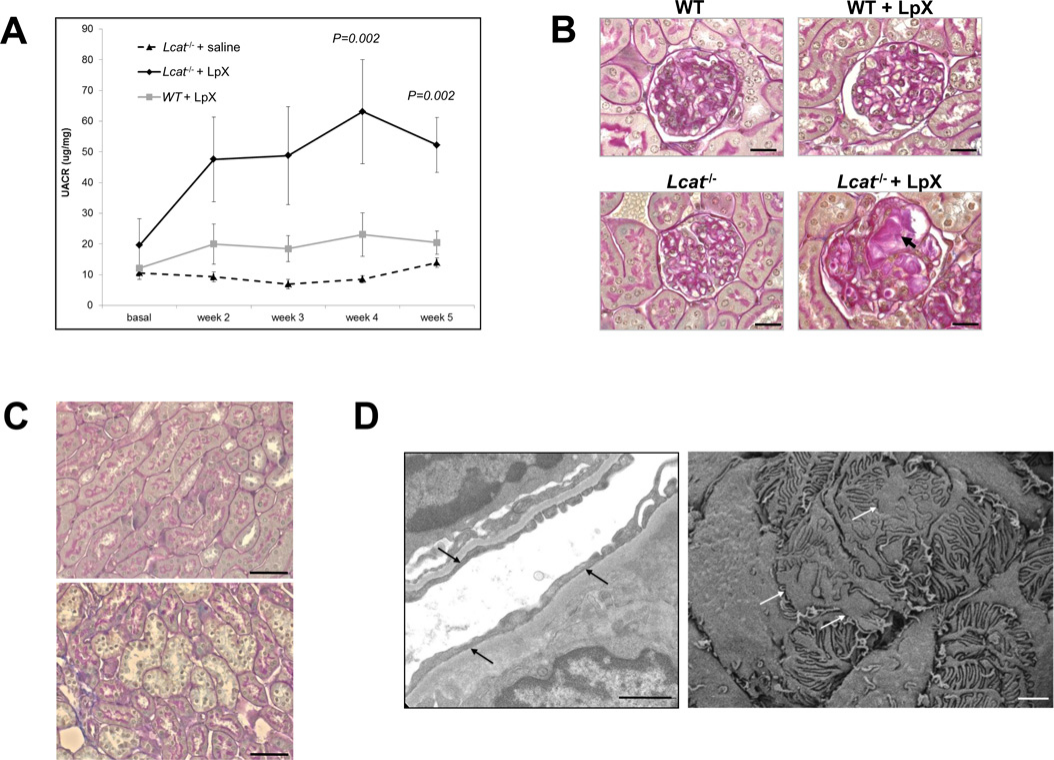
LpX induces nephropathology in *Lcat*^-/-^ mice. (A) Effect of LpX injection on renal function. Albumin to creatinine ratios (μg/mg) in urine (UACR) were measured prior to and then every week after exogenous LpX treatment, starting on week 2. Data are expressed as mean ± SEM. P values of group differences at each time point are reported. (B) Histological analysis. Representative images of PAS-stained sections of kidneys from WT and *Lcat*^-/-^ mice treated or not treated with LpX. No histological alterations were present in WT mice. WT mice treated with LpX showed no changes or only mild mesangial matrix expansion. In *Lcat*^-/-^ mice, LpX treatment increased mesangial matrix (*asterisk*) and, occasionally, PAS-positive material in glomerular capillaries (*arrow*) was observed (Scale bars: 20 μm). (C) Representative images of PAS-stained sections of kidneys from WT (*upper panel*) and *Lcat*^-/-^ (*lower panel*) mice treated with LpX. Tubular cell vacuolation was present focally in *Lcat*^-/-^ mice. (Scale bars: 50 μm). (D) Podocyte effacement revealed by TEM (left, *black arrows*; Scale bar: 1 μm) and SEM (right, *white arrows*; Scale bar: 20 μm) in glomeruli of *Lcat*^-/-^ mice treated with LpX.

We next assessed the *in vivo* catabolism and remodeling of unlabeled synthetic LpX lipids in WT and *Lcat*^-/-^ mice by FPLC analyses, but used a lower dose of LpX (0.5 mg dose three times a week for 5 wks) to generate conditions more likely to selectively cause renal damage in *Lcat*^-/-^ mice. Similar to our fluorescent tagged studies, LpX showed delay clearance in *Lcat*^-/-^ mice (Fig 4A). Interestingly, we also observed in these studies the formation of a small phospholipid-rich cholesterol-poor HDL particle in *Lcat*^-/-^ mice after LpX administration (Fig 4B and 4C). To assess the effect of exogenous LpX administration on renal function, urine samples were collected before the start of LpX treatment and at the end of each week, starting on week 2. *Lcat*^-/-^ mice injected with LpX (Fig 5A) showed a constant increase in the urine μg albumin/mg creatinine ratio (UACR) that increases more than two-fold at the fourth and fifth weeks of treatment (63.1±16.97 and 52.2±8.93 vs 19.6±8.64 μg/mg; *P* = 0.002, One Way ANOVA). In contrast, *Lcat*^-/-^ mice injected with saline solution (Fig 5A), the UACR remained relatively stable and comparable to baseline. In WT mice injected with LpX (Fig 5A), a small trend toward increasing UACR values was observed but did not reach statistical significance (*P* = 0.106, One Way ANOVA). At the last week of treatment, UACR values were significantly increased in *Lcat*^-/-^ mice compared to other two groups (*Lcat*^-/-^+LpX vs *Lcat*^-/-^+saline *P*<0.05; *Lcat*^-/-^+LpX vs WT+LpX *P*<0.05; *Lcat*^-/-^+saline vs WT+LpX *P*>0.05).

We did not observe any significant change in plasma creatinine levels in *Lcat-/-* mice compared to WT, nor with with LpX treatment in either WT (0.10 ± 0.01 *vs* 0.11 ± 0.01, with and without LpX, respectively), or *Lcat*^-/-^ mice (0.10 ± 0.00 *vs* 0.11 ± 0.01 mg/dL, with and without LpX, respectively, mean ± S.D.; p = 0.350; One Way ANOVA). Similarly, BUN levels were not altered either by *Lcat* deficiency or LpX treatment (20.44 ± 1.46 *vs* 20.87 ± 2.24, WT mice with and without LpX, respectively; (22.86 ± 6.20 *vs* 21.54 ± 2.56 mg/dL, *Lcat*^-/-^ mice with and without LpX, respectively, mean ± S.D.; p = 0.753, One Way ANOVA). This is consistent with FLD, where typically plasma creatinine and BUN levels are normal early in the disease and increase only with the onset of ESRD [2].

### *Lcat* Deficiency Alters Glomerular Morphology and Induces Podocyte Effacement

We further assessed the effect of exogenous LpX on WT and *Lcat*^-/-^ mouse kidney morphology by light and EM microscopy. PAS-staining revealed that before LpX treatment neither WT nor *Lcat*^-/-^ mice showed any histologic abnormalities in the glomeruli (Fig 5B). After LpX treatment, the glomeruli of WT mice showed no changes or only some mild mesangial matrix expansion (Fig 5B; upper panels). In contrast, approximately 60% of the glomeruli of *Lcat*^-/-^ mice treated with LpX (Fig 5B, *lower middle panel*), had significantly increased glomerular mesangial matrix accumulation as determined by PAS staining (Score: LCAT+LpX: 0.9±0.2 *vs* WT+LpX: 0.2±0.02, P<0.05, non-parametric Mann Whitney test). However, we did not see evidence of the infiltration of inflammatory cells into renal glomeruli. *Lcat*^-/-^ mice treated with LpX also showed mild tubular injury, consisting of focal tubular cell vacuolation (Fig 5C), in the absence of inflammatory cell infiltration. These histological findings are consistent with our observation that fluorescent PE-tagged LpX was found to accumulate in *Lcat*^-/-^ renal tubules (S2 Fig). SEM analysis revealed that podocyte foot processes appeared normal in WT mice chronically treated with LpX (S9 Fig). However, *Lcat*^-/-^ primary and secondary podocyte foot processes exhibited minor morphological alterations (*bulging*: S9 Fig). Transmission (Fig 5D, *left* panel) and scanning electron microscopic (Fig 5D, *right* panel; S9 Fig) analyses of glomerular pathology also revealed focal foot process effacement in the *Lcat*^-/-^ mice treated with LpX, which is commonly seen in patients with proteinuria [29].

### LpX Endocytosed by Renal Glomerular Cells is Degraded in Lysosomes

**Fig 6:**
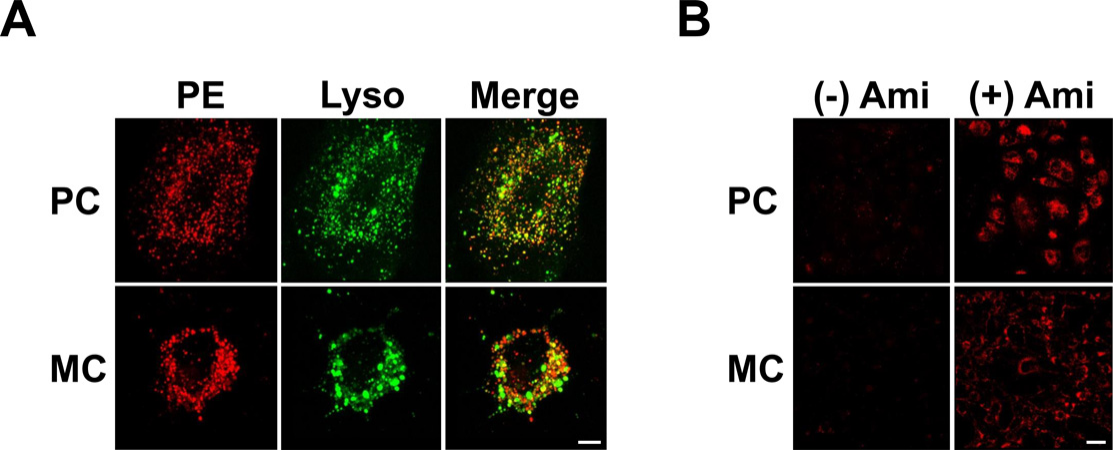
LpX internalized by glomerular cells *in vitro* is degraded in lysosomes. (A) LpX endocytosis. Immortalized human podocytes (PC) and mesangial cells (MC) were incubated with 200 μg LpX for 18 hrs, and then stained with Lysotracker. Note fluorescent PE-tagged LpX (*red*) extensively colocalized with Lysotracker (*green*), seen as *yellow-orange* vesicles in the merged image. Scale bar: 10 μm. (B) Inhibition of lysosomal PLA_2_ increases renal glomerular cellular LpX uptake in vitro. PCs and MCs were incubated in the absence ((-) Ami) or presence ((+) Ami) of the lysosomal PLA_2_-specific inhibitor amiodarone (5 μM) overnight and then incubated with 200 μg/ml fluorescent PE-tagged synthetic LpX for 4 hrs. Amiodarone markedly increased LpX in both PC and MC lysosomes suggesting that endocytosed LpX phospholipid is degraded in lysosomes. Scale bar: 50 μm. Note imaging of PE fluorescence was optimized for amiodarone-treated cells in (B) to allow for comparison of relative LpX upake with and without treatment.

To better understand the mechanisms underlying LpX nephrotoxicity, we next monitored renal glomerular cell uptake of fluorescent PE-tagged LpX *in vitro* by confocal microscopy. Cultured immortalized human podocytes (PC) and mesangial cells (MC) were incubated with fluorescent PE-tagged LpX, as well as Lysotracker, a lysosomal marker, in the absence or presence of amiodarone, a specific inhibitor of lysosomal PLA_2_ (LPLA_2_) [30]. Snake venom PLA has long been known to degrade LpX *in vitro* [31]. Both cell types were observed to endocytose fluorescent LpX into punctate vesicles, and a large portion of the LpX colocalized with Lysotracker (Fig 6A). Amiodarone markedly increased retention of LpX in podocyte and MC lysosomes (Fig 6B), suggesting that LPLA_2_ degrades LpX phospholipids in lysosomes.

### LpX Compromises the Integrity of Endothelial Cell Monolayers

**Fig 7:**
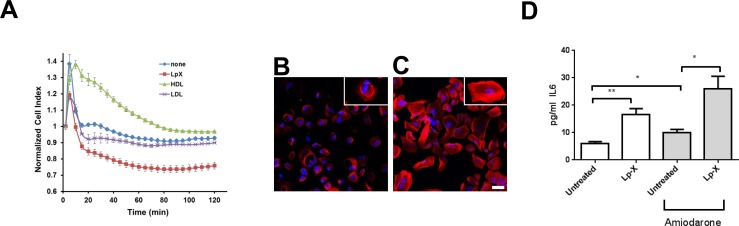
Effect of LpX on glomerular cell function *in vitro.* (A) LpX compromises the integrity of endothelial cell monolayers. Confluent HUVEC cell monolayers were incubated with PBS alone, or with PBS containing HDL (1 mg/ml), LDL (1 mg/ml), or LpX 5 mg/ml). Following a transient artifactual increase in impedance after the change in medium, LpX markedly decreased impedance. (B,C) LpX alters podocyte cytoskeletal actin organization. Representative confocal images of phalloidin-stained immortalized human podocytes incubated in vitro in the absence (B) or presence of (C) synthetic LpX. Scale bar: 100 μm.(D) LpX endocytosis stimulates mesangial cell IL-6 cytokine secretion in vitro. Confluent cultured mesangial cells were treated with 200 μg/ml fluorescent PE-tagged synthetic LpX, in the absence or presence of 5 μM amiodarone for 18 hrs, and then IL-6 was quantified by ELISA assay. Note that amiodarone further increased IL-6 secretion with or without LpX treatment. *p <0.05; **p = 0.01; unpaired two-tailed t-test.

The EC monolayer that lines glomerular capillaries together with the GBM and podocyte slit diaphragms play a critical role in the barrier function that determines the composition of the plasma ultrafiltrate that enters the glomerular urinary space [32]. We used HUVEC cells grown as tight monolayers on Transfilters as an *in vitro* model to access the potential effect of LpX on the integrity of the glomerular capillary endothelium (Fig 7A). Compared to the PBS control, addition of HDL markedly increased the impedance across HUVEC cell monolayers, as previously reported [33], whereas LDL slightly decreased impedance. In contrast, LpX markedly decreased impedance, indicating that LpX may interfere with EC barrier function and promote its infiltration into the glomerulus.

### LpX Alters Podocyte Cytoskeletal Organization

As an indirect measure of podocyte function, we next assessed the effect of LpX treatment on the cytoskeleton of podocytes by monitoring actin-specific phalloidin staining by confocal microscopy (Fig 7B and 7C). The podocyte cytoskeleton is well known to play several important roles in maintaining the normal barrier function of podocytes and cytoskeletal rearrangements are associated with aberrant pathophysiological alterations in podocyte structure and its barrier function [29,34]. We found that LpX treatment markedly altered cellular actin organization (Fig 7B and 7C), generating tightly packed bands of actin that appear to resemble cytoskeletal rearrangements associated with podocyte effacement [29].

### LpX Increases IL-6 Cytokine Secretion by Mesangial Cells

IL-6 is an autocrine pro-inflammatory cytokine secreted by MC’s in response to stressful stimuli [35,36]. Given the observed uptake of LpX by MCs both *in vivo* (Fig 3J; S8 Fig) and *in vitro* (Fig 6), we tested if LpX would stimulate IL-6 secretion by MCs *in vitro*. As shown in Fig 7D, LpX increased MC IL-6 secretion approximately 3-fold both in the absence or presence of amiodarone compared to non-LpX-treated controls. Amiodarone also increased lysosomal LpX content (Fig 7D), likely by inhibiting LPLA_2_-mediated degradation.

### Chronic LpX Treatment Increases Expression of Nephrotoxic Genes in *Lcat*^-/-^ Mice *In Vivo*

**Fig 8:**
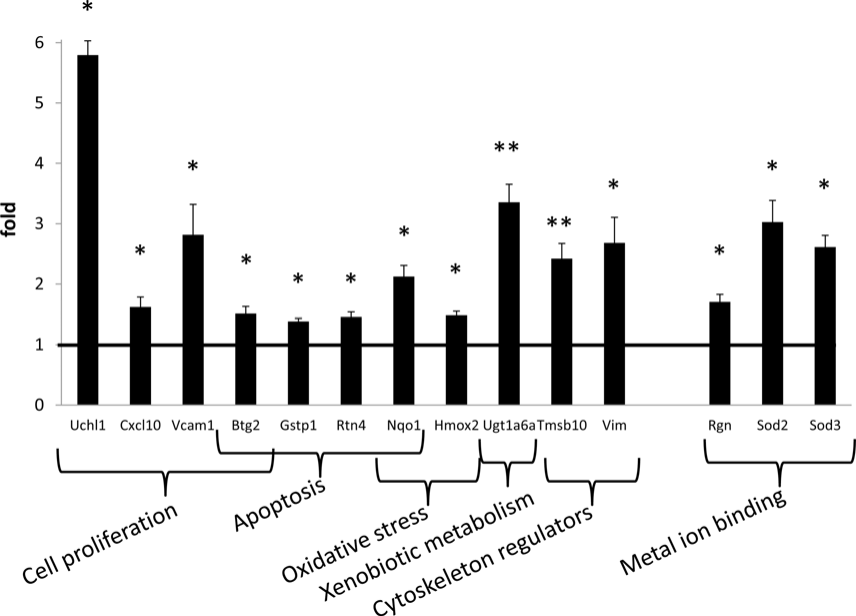
Kidney gene expression in *Lcat-/-* mice after LpX treatment. The expression of 84 genes involved in nephrotoxic pathways was measured. Only genes whose expression was statistically different between LpX- treated *vs* saline-treated *Lcat*^-/-^ mice are reported. Data are expressed as mean ± SEM. * P<0.05, ** P<0.01, paired t-test.

The expression of a panel of genes known to be involved in the nephrotoxicity was analyzed in *Lcat*^-/-^ mice either chronically-treated with LpX (n = 5), or with normal saline (n = 5). The complete list of genes tested and the degree of alteration in expression level with LpX treatment is shown in S1 Table. Overall, 14 out of the 84 genes tested showed significant upregulation after LpX treatment compared to the saline controls (Fig 8). Genes involved in multiple pathways known to be altered by nephrotoxicity showed expression changes, including those involved in apoptosis, oxidative stress, cytoskeleton regulation and cell proliferation. Although we did not assess corresponding protein changes, the altered transcriptomic levels were consistent with the observed alterations in renal morphology at the light and EM levels and functional alterations, i.e., proteinuria.

The observed LpX-induced increase in cytoskeletal regulatory gene expression *in vivo* (Vim, Tmsb10), and LpX-induced stress fiber formation *in vitro* (Fig 7B and 7C) correlated with the LpX-induced podocyte effacement observed *in vivo*. Increased expression of the intermediate filament protein vimentin in podocytes has previously been shown to occur in other nephrotic syndromes and to be associated with altered podocyte cell signaling, cell shape and adhesion to the GBM [37,38].

## Discussion

Although long implicated, there is limited direct experimental evidence that LpX causes renal disease in FLD. Now that recombinant LCAT is being developed as a potential treatment for this disorder [22,39], it is important to determine if LpX is nephrotoxic and, therefore, can be used as a biomarker in FLD patients treated with recombinant LCAT. Herein, we demonstrate that LpX alone is sufficient to induce proteinuria and have gained new insights into the mechanisms underlying LpX-mediated renal pathophysiology in LCAT deficiency.

The synthetic LpX used in this study had a similar lipid composition, and size distribution compared to endogenous LpX [2,5] and was also structurally similar to endogenous LpX (Fig 2D). Our *in vitro* studies (Fig 1) also demonstrated that synthetic LpX behaved like endogenous LpX in its interaction with apoA-I and LCAT [13]. ApoA-I removed both phospholipid and cholesterol from synthetic LpX, forming a new particle that was remodeled into an HDL-like particle in the presence of LCAT. ApoA-I bound to LpX was required to activate LCAT. Human serum also remodeled synthetic LpX *in vitro* to form an HDL-like particle. Together, these findings strongly suggest that LCAT along with apoA-I mediates conversion of LpX to HDL-like particles.

When LpX was chronically administered to WT mice at a relatively low dose, the animals rapidly cleared plasma LpX and did not develop proteinuria or any notable changes in renal glomerular morphology, as assessed by histological and EM analyses. In contrast, LpX plasma clearance was markedly delayed in *Lcat*^-/-^ mice, consistent with the previous finding that plasma clearance of radiolabeled LpX in FLD patients is delayed compared to normal subjects [40]. Together, these results suggest that normal physiological levels of LCAT are sufficient to remodel LpX into HDL, but in the absence of plasma LCAT, high levels of plasma LpX persist. The marked reduction of apoA-I that secondarily occurs in FLD likely further contributes to LpX accumulation, given the requirement for apoA-I in LCAT-mediated conversion of LpX to HDL (Fig 1). A considerable portion of the plasma LpX in *Lcat*^-/-^ mice was deposited in the kidneys, where it induced the same pathological hallmarks seen in FLD [2], namely proteinuria, GBM and endothelial damage, podocyte effacement, expansion of the mesangial matrix and renal tubule vacuolation.

The glomerular capillary filter consists of the following three main filtration barriers: (i) the endothelial surface layer with the endothelial cell (EC) glycocalyx [41–43] and the EC fenestrations that are approximately 70–100 nm in diameter [44]; (ii) the GBM, which provides a fibrillary network with pores ranging from 10 to 40 nm in diameter [32,45] and finally, (iii) the 40 nm slit diaphragm [46] between adjacent podocyte foot processes (FPCs). Our studies indicate that LpX either directly or indirectly may affect all three of these filtration barriers, thus likely accounting for its ability to induce proteinuria.

The glomerular capillary endothelium and its glycocalyx, together with the endothelial surface layer enriched with proteoglycans, provide the initial barrier to entry of the relatively large LpX particles into the urinary space. We found that binding of both endogenous and exogenous LpX to glomerular capillary ECs appeared to be indistinguishable. LpX binding to ECs induced all the features of macropinocytosis, including membrane ruffling, formation of lamellipodia, fusion of lamellipodia with the plasma membrane, and formation, internalization and intracellular trafficking of macropinosomes [47]. The observed activation of glomerular capillary ECs by LpX may play an important role in LpX-mediated proteinuria since glomerular EC injury alone, as seen in pre-eclampsia and thrombotic microangiopathies, can cause proteinuria [43,44]. In addition, the glomerular endothelium is similarly damaged in a number of other conditions, including diabetic nephropathy and transplant glomerulopathy [43]. LpX internalized by ECs *in vitro* compromised cell monolayer integrity and LpX was observed to penetrate the GBM *in vivo*. Together these findings suggest a model in which LpX-induced lamellipodia formation and macropinocytosis alters EC morphology, widening glomerular capillary endothelial fenestrations, thereby facilitating the entry of LpX particles into the GBM. Additional support for this model is provided by the concomitant increase in proteinuria and the diameter of endothelial fenestrae (3-fold) in the absence of altered podocyte morphology or effacement following LPS administration to mice in vivo [48].

We observed that after LpX penetrates the glomerular endothelial barrier, some of it is trapped in the GBM (Fig 3). The GBM is enriched in negatively charged proteoglycans [46], which could possibly retain LpX due to its net positive charge, demonstrated by its cathodal migration on agarose gel electrophoresis (Fig 1). Once in the GBM, LpX disrupted the local GBM matrix (Fig 3), possibly as a result of mechanical disruption of the matrical components, or by activation of EC [41–44] or podocyte [42] secretion of extracellular proteases. Damage to, or activation of, ECs by cytokines, mechanical stress and other factors, induces formation of endothelial podosomes; these are actin-rich adhesion structures that remodel the underlying BM matrix via release of metalloproteinases and serine proteinases which degrade the extracellular matrix [49–51]. Given that proteinase activity may also occur at lamellipodia [49], it is possible that EC lamellipodia extending into the GBM may have secreted proteinases at the sites where we observed concomitant LpX penetration and matrix degradation (Fig 3). It is currently thought that GBM abnormalities can also lead to proteinuria through disruption of the cellular contribution to the barrier [43].

In order for LpX to enter into the glomerular space, it would have to pass through the podocyte slit diaphragm, currently thought to provide a pore size of 40 nm, which is much smaller than many of the LpX particles we observed in the urinary space. Based on a large number of *in vitro* and *in vivo* studies, it is currently thought that an intricate and abundant signaling network utilizing growth factors, cytokines, chemokines, proteases, and other bioactive molecules provides cross-talk between ECs, podocytes, and MCs that regulate the development and maintenance of the glomerular filtration barrier, all of which if dysfunctional contribute to the pathogenesis of proteinuria and nephropathy [42,52–54]. Thus, signaling molecules released by ECs and/or MCs could alter podocyte slit diaphragm morphology, rendering it permeable to larger LpX particles. The observed robust deposition of LpX particles in the GBM near the EC–MC interface could also potentially activate MC signaling. In addition since MCs at or near the hilum of the capillary loop are often in direct contact with the non-fenestrated EC body [44], cytokines secreted by LpX-activated ECs and MCs might also provide signals that could alter podocyte slit diaphragm integrity. Our finding that LpX stimulated IL-6 cytokine secretion from MCs *in vitro* is also consistent with this scenario, as is the increased LpX-induced expression of the cytokine receptor CxCl10 we reported here *in vivo*. In addition, chemokines secreted by MCs activate ECs and up-regulate adhesion molecules, further facilitating inflammation [53]. Podocytes actively and abundantly removed LpX within the urinary space by macropinocytosis, providing the opportunity for LpX-stimulated secretion of podocyte signaling factors that could in turn target both ECs and MCs. Interestingly, albumin-associated FFAs have been shown to induce macropinocytosis in podocytes and the podocyte response to FFAs has been proposed to function in the development of nephrotic syndrome by amplifying the effects of proteinuria [55].

Although it is not thought to be a major contributor to the renal dysfunction of FLD, we observed uptake of LpX into renal tubular cells (S2 Fig) and renal tubular vacuolation (Fig 5). Similar renal tubular damage has also been described in FLD [2], which is probably a consequence of the severe proteinuria and uptake of excess urinary proteins and possibly LpX by the tubular cells.

In addition to LpX trafficking through the various renal compartments, results from this study also revealed the intracellular trafficking of LpX in glomerular cells. Intact LpX particles were observed in both forming and internalized macropinosomes in both EC and podocytes by TEM. LpX particles displaying varying degrees of degradation were also observed in large intracellular vacuoles that appear to represent lysosomes/phagolysosomes. This was confirmed *in vitro* insofar as fluorescent tagged LpX colocalized with Lysotracker in MCs and podocytes (Fig 6). Lysosomal PLA_2_ (LPLA_2_) activity has been previously detected in the kidney cortex [30]. When cells were treated with amiodarone, an antiarrhythmic drug that inhibits LPLA_2_ activity, markedly increased lysosomal accumulation of LpX was observed (Fig 6). These findings suggest that LPLA_2_ may mediate lysosomal LpX degradation. Both LCAT and lysosomal PLA_2_ share structural similarity in their phospholipid binding and catalytically active sites [56]. Unlike LCAT however, LPLA_2_ does not require apoA-I activation, and is only active in an acidic environment, rendering it susceptible to inhibition by a variety of other commonly used cationic amphipathic drugs that interfere with lysosomal acidification and cause phospholipidosis in otherwise normal subjects [30]. Thus, LPLA_2_-mediated hydrolysis of LpX phospholipids in lysosomes is likely to be an important step in the normal intracellular processing of LpX, leading to the generation of FFAs and cholesterol that can be delivered to other cellular sites. Excess intracellular cholesterol, oxidized cholesterol and FFAs in podocytes, MCs, and renal tubular cells generated after lysosomal catabolism of LpX can possibly disrupt normal cell function and thus could contribute to the pathology of FLD [57–59]. Given the increased secretion of pro-inflammatory IL-6 observed in MCs treated with LpX in the presence of amiodarone (Fig 7), amiodarone and other drugs that cause phospholipidosis could potentially exacerbate the renal disease associated with FLD.

In summary, we have shown that LpX is remodeled by LCAT and apoA-I and that LpX alone is sufficient to induce many of the renal pathologic features seen in FLD renal disease, and hence may be a suitable biomarker for monitoring the response to any new therapy for FLD, such as recombinant LCAT.

## Supporting Information

S1 Fig LpX clearance from blood compartments. WT (n = 5) and *Lcat*^-/-^ mice (n = 5) were chronically-treated with unlabeled exogenous LpX (3 mg/wk for 4 wks), and then plasma and RBC blood compartments were analyzed 4 hrs after a final injection with fluorescent PE-tagged LpX. (A) LpX clearance is markedly delayed in *Lcat*^-/-^ mice. Agarose gel of WT and *Lcat*^-/-^ mouse plasma samples stained with filipin, which specifically binds to unesterified cholesterol, to reveal the presence of plasma LpX *(upper panel*), or, scanned for PE fluorescence (*lower panel*). Little, if any of the injected LpX remained in WT plasma, whereas LpX levels *Lcat*^-/-^ mouse plasma remained elevated, consistent with the results reported after a bolus injection of 1 mg fluorescent PE-tagged LpX (Fig 2A & 2B). (B) LpX binding to RBCs is markedly increased in *Lcat*^-/-^ mice. RBC lipids were extracted and PE fluorescence was measured. Data are expressed as mean ± S.D. * P < 0.0001; unpaired two-tailed t-test.
(PDF)

S2 Fig *Lcat* deficiency increases accumulation of synthetic fluorescent LpX in mouse renal glomeruli and tubules. Frozen sections of kidneys of chronically LpX-treated WT and *Lcat*^-/-^ mice were briefly fixed and imaged by confocal microscopy as described in “Methods.” Representative maximum projection image of 16 μm sections.
(PDF)

S3 Fig Macropinocytotic uptake of LpX by WT renal glomerular endothelial cells. Gallery of EM images. (A) Two multiloculated LpX particles are seen bound to an endothelial cell (EC) lining a renal capillary. Note the EC surface ruffling where the LPX binds (*white arrows*). (B) LpX particle (*arrow*) binding to a renal EC lamellipodium. *Inset*: Higher magnification. *Arrow*: EC lamellipodium. (C) LpX particles bound to the EC surface (*small arrows*) are engulfed by lamellipodia (asterisks). (D) Multiloculated LpX particles are seen bound to the surface of an EC *(large white arrow*) and to an EC lamellipodium (*large white arrowhead*). LpX particles internalized by the EC are indicated by the *small white arrows* and LpX within the capillary lumen by the *small black arrows*. (E) Renal capillary EC engulfment of LpX particles. The ends of the EC lamellipodia engulfing the LpX particles are indicated by the *small black arrows*. (F) LpX engulfed by an EC is indicated by the *black arrow*. (G-K) EC lysosomes containing degraded LpX pariticles (*arrows*). *Inset* in (H): Higher magnification of lowermost EC. (L) A partially degraded LpX particle in a renal capillary lumen is bound to, and bridging two RBCs. *Insets*: Higher magnification of LpX contact points with RBCs. (M,N) Matrix degradation (indicated by *asterisks*) in GBM in regions containing numerous LpX particles. Scale bars: I = 100nm; L1,2 = 125 nm; A, D, E = 200 nm. H, H *inset*, B *inset*, J = 250nm; B, C, F-H, K-N = 500 nm.
(PDF)

S4 Fig Macropinocytotic uptake of LpX by *Lcat*^-/-^ renal glomerular endothelial cells. Gallery of EM images. (A) A multiloculated LpX particle bound to an RBC *(upper arrow*) also binds to the surface of an EC (*lower arrow*) lining a renal capillary. (B) An EC lamellipodium (*arrow*) binds an LpX particle. (C) An EC binding an LpX particle extends lamellipodia (*arrows*), initiating LpX internalization. (D) Enlarged version of Fig 3B. (E) Upper EC with LpX-containing vacuoles (1,2,3) extends a lamellipodium (on *left*) in close proximity to a lamellipodium extended by the lower EC (*asterisks*), enclosing numerous LpX particles (E3). The upper and lower ECs appear to have fused at points of contact (*arrows*), enclosing several LpX particles. (F) An EC extends a lamellipodium into the GBM enclosing several LpX particles (*arrow*). (G) Large multilamellar LpX particles (*large arrows*) internalized by an EC as well as small LpX particles infiltrating the GBM (*small arrows*) are seen. (H) EC lamellipodium (*large arrow*) encloses LpX particles within an EC. Note LpX in the urinary space binds to PCB and PFPs (*small arrows*). (I) EC lamellipodium fuses with EC body at points of contact (*arrows*), enclosing LpX particles. (J) EC lamellipodia encircle an LpX particle (*arrowhead*). Note phagolysosome (*asterisk*) and multivesicular body (MVB) (*arrow*) containing dergraded LpX. (K,L) Phagolysosomes (*arrows*) containing degraded LpX in renal capillary endothelium. (M) EC contains MVB with LpX remnants (*large arrow*). LpX particles penetrate the GBM (*small arrow*), disrupting the GBM matrix (*asterisk*). (N) Fig 3D enlarged. (O) Numerous LpX particles penetrate the GBM (*arrows*), disrupting the GBM matrix (*asterisks*). (P-T) LpX particles penetrate the GBM (*arrows*), disrupting the GBM matrix. Scale bars: E1, E2, E3, P, R = 100 nm; F, G = 200 nm; D1 = 250 nm; H = 400 nm; A-D, D2, E, I–O, S, T = 500 nm.
(PDF)

S5 Fig WT mouse glomerular podocyte LpX uptake. Gallery of EM images. (A-K) LpX particles bind to PCBs and PFPs (*arrows*) in the urinary space (US; indicated in (A)). *Double asterisks* in (B, F) denote large lipid droplets within a PCB, and, *single asterisks* denote representative vacuoles seen in PCBs (D,J,M-P). Note the membrane ruffling at sites of LpX binding in (E,F). (L-R) PCBs and PFPs also often contain multivesicular bodies (MVB) with numerous small unilamellar vesicles (*black arrows*). (O,Q,R) Larger MVBs were also seen to contain partially degraded LpX particles. CL = capillary lumen; GBM = glomerular basement membrane. Scale bars: B, D, O = 200 nm; L, N, P = 300 nm; K = 400 nm; A, C, E–J, M, Q, R = 500 nm.
(PDF)

S6 Fig *Lcat*^-/-^ mouse glomerular podocyte LpX uptake. Gallery of EM images. (A-N) LpX particles bind to PCBs and PFPs (arrows) in the urinary space (US; indicated in (A)). (C,F) *Asterisks* denote representative vacuoles seen in PCBs. A multilamellar LpX particle in a phagosome is indicated by the *arrowhead* in (N). (O-W) LpX degradation in podocyte phagolysosomes. MVBs containing small unilamellar vesicles as well as large phagolysosomes containing both partially degraded LpX particles as well as unilamellar vesicles are indicated by *white* and *black* and *arrows*, respectively. LpX particles in the urinary space are indicated by the *arrowhead* in (O) and small arrow in (S). (U) and (V) are enlargements of (S) and (T), respectively. Note the large phagosome containing a large multilamellar LpX particle in a renal capillary EC in (P) (*small white arrows*). Scale bars: U, V, X = 100 nm; A, O, T = 200 nm; W = 250 nm; N = 300 nm; B–M, P-S = 500 nm.
(PDF)

S7 Fig LpX movement into WT mouse glomerular mesangium. Gallery of EM images. Lpid deposition in mesangial cells (MC) and matrix (MM). (A) Foamy MC containing numerous fat droplets. (A1) Higher magnification of the cell in (A). (B) Fat deposition in MM (*black arrow*). Scale bars: 500 nm.
(PDF)

S8 Fig LpX movement into *Lcat*^-/-^ mouse glomerular mesangium. Gallery of EM images. (A) LpX accumulates in the mesangial matrix and is taken up by mesangial cells (MC) by macropinocytosis. LpX binds to MCs which extend lamellipodia (*black arrows*) entrapping matrical LpX. Regions enclosed by *white boxes* in (A) are enlarged in (A1, A2). Fat droplets within MCs are denoted by asterisks, and MC lamellipodia entrapping LpX particles are indicated by *black arrows*. (A, B) Very large macropinosomes in the process of formation are seen to enclose numerous LpX particles ((A2) *large white arrow*; (B)). In (C), a renal capillary EC near the mesangium contains a large phagosome enveloping a large LpX particle, and abundant deposition of LpX particles concomitant with matrix degradation is seen in the nearby mesangial matrix (MM). (D-G). Additional examples of LpX binding to the surface of MCs, MC lamellipodial extension,engulfment of matrical LpX and in (F), internalization and degradation of LpX in MC phagolysosomes. (H,I) Enlargements of regions in (G) showing LpX binding to MC lamellipodia. (J-N) Abundant deposition of LpX in MM. Scale bars: B = 100 nm; A2 = 200 nm; M = 300 nm; A, A1, C–N = 500 nm.
(PDF)

S9 Fig Podocyte foot processes are altered in *Lcat*^-/-^ mouse glomeruli. Representative SEM images of WT (*left panels*) and *Lcat*^-/-^ (*right panels*) podocytes in the absence (*upper panels*) and presence (*lower panels*) of chronic LpX administration. Primary and secondary podocyte foot processes are indicated by *small* and *large arrows*, respectively. WT processes appear to be unaltered by LpX treatment (WT *vs* (WT + LpX)). Note the altered morphology of both primary and secondary podocyte processes in *Lcat*^-/-^ glomeruli with or without LpX; (*Lcat*^-/-^ + LpX) and (*Lcat*^-/-^—LpX), respectively. In *Lcat*^-/-^ mice, several primary and secondary processes are bulged, exemplified by the *small* and *large arrows*, respectively; in *Lcat*^-/-^ mice + LpX, note the bulged primary process (*small arrow*) and focal foot effacement (fused foot processes*; large arrow*). Scale bar = 20 μm.
(PDF)

S1 Table Kidney gene expression after LpX treatment in *Lcat-/-* mice. The expression of a panel of genes known to be involved in the nephrotoxicity was analyzed in *Lcat*^-/-^ mice either chronically-treated with LpX (n = 5) or with saline (n = 5). Differences between LpX- treated *vs* saline-treated *Lcat*^-/-^ mice are reported in this table. Genes whose expressionare statistically different (Paired t-test) are highlighted.
(DOCX)
