# Supplementary figures and images for: Lipoprotein X Causes Renal Disease in LCAT Deficiency
Source: PLoS One. 2016 Feb 26;11(2):e0150083. doi: 10.1371/journal.pone.0150083 (PMC4769176; doi:10.1371/journal.pone.0150083)

# S1

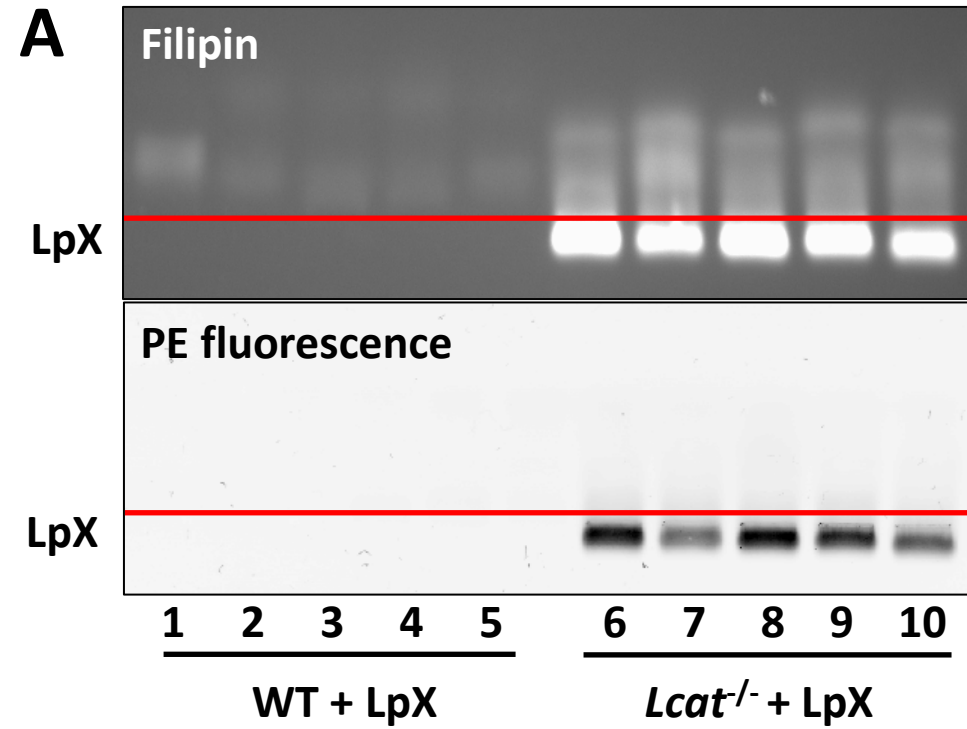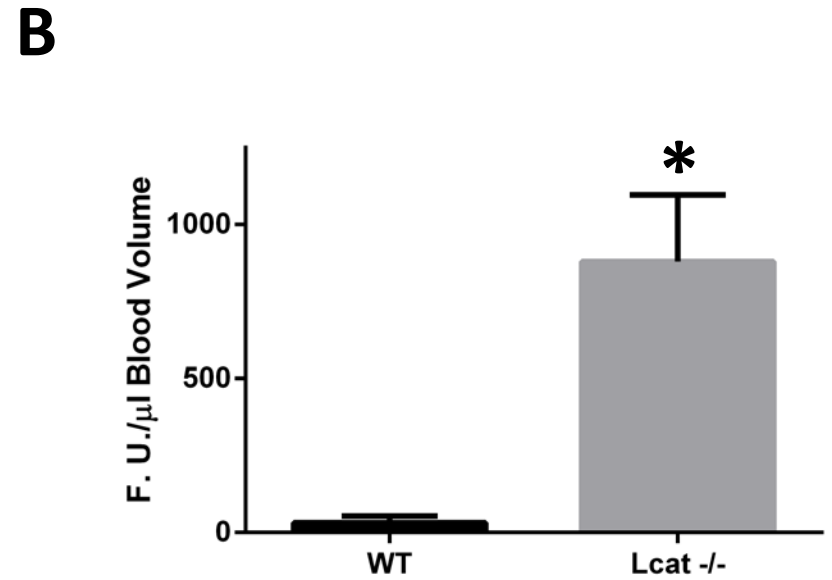

Supplement: S1 Fig — WT (n = 5) and Lcat-/- mice (n = 5) were chronically-treated with unlabeled exogenous LpX (3 mg/wk for 4 wks), and then plasma and RBC blood compartments were analyzed 4 hrs after a final injection with fluorescent PE-tagged LpX. (A) LpX clearance is markedly delayed in Lcat-/- mice. Agarose gel of WT and Lcat-/- mouse plasma samples stained with filipin, which specifically binds to unesterified cholesterol, to reveal the presence of plasma LpX (upper panel), or, scanned for PE fluorescence (lower panel). Little, if any of the injected LpX remained in WT plasma, whereas LpX levels Lcat-/- mouse plasma remained elevated, consistent with the results reported after a bolus injection of 1 mg fluorescent PE-tagged LpX (Fig 2A & 2B). (B) LpX binding to RBCs is markedly increased in Lcat-/- mice. RBC lipids were extracted and PE fluorescence was measured. Data are expressed as mean ± S.D. * P < 0.0001; unpaired two-tailed t-test. (PDF) [file pone.0150083.s001.pdf]

## S2

**WT**

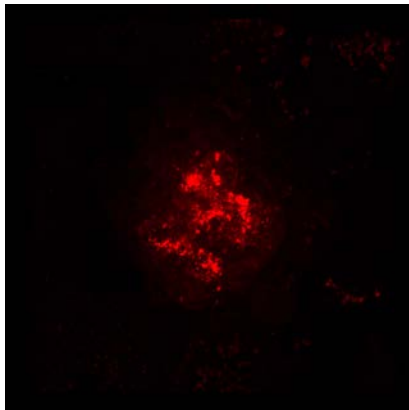

**Renal Glomerulus**

***Lcat*<sup>-/-</sup>**

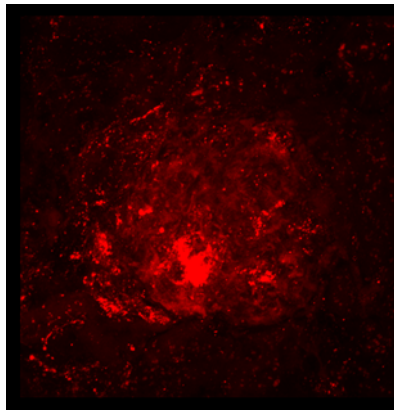

**Renal Glomerulus**

***Lcat*<sup>-/-</sup>**

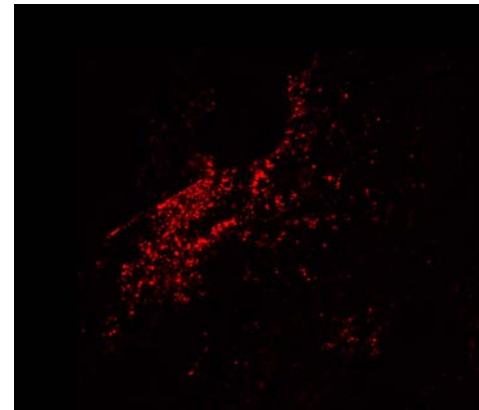

**Renal Tubule**

Supplement: S2 Fig — Frozen sections of kidneys of chronically LpX-treated WT and Lcat-/- mice were briefly fixed and imaged by confocal microscopy as described in “Methods.” Representative maximum projection image of 16 μm sections. (PDF) [file pone.0150083.s002.pdf]

### S3: Macropinocytotic uptake of LpX by normal renal glomerular endothelial cells

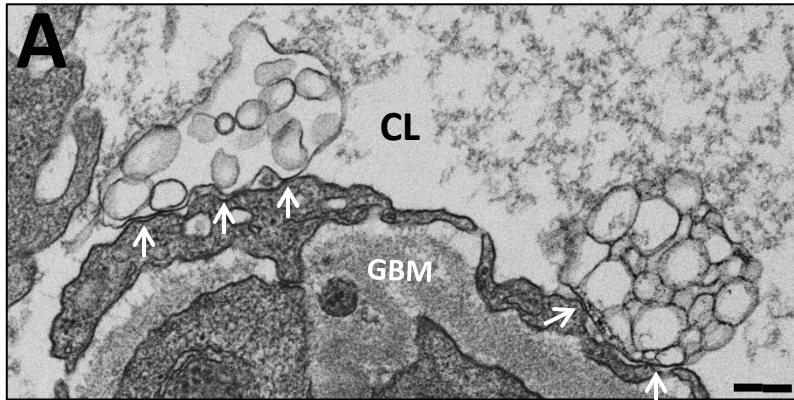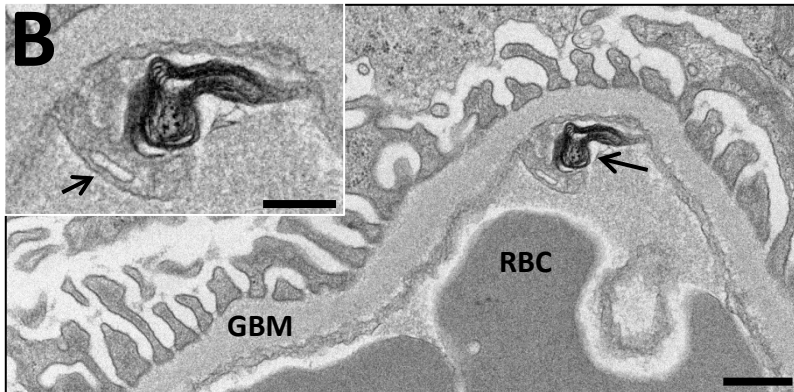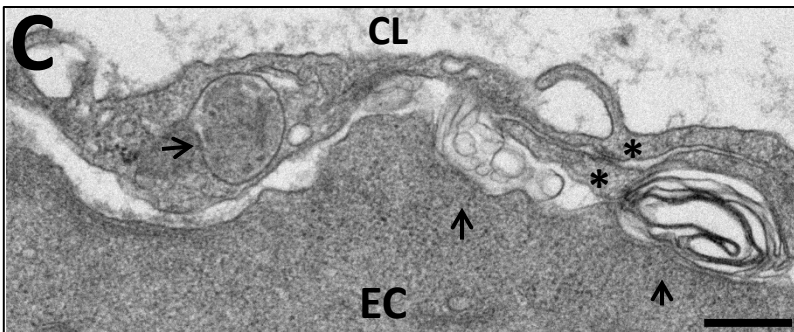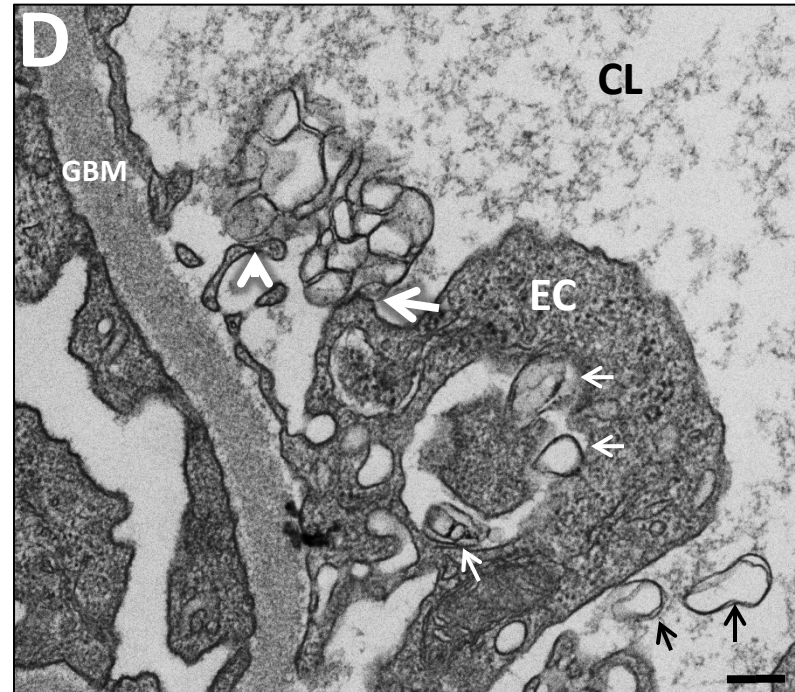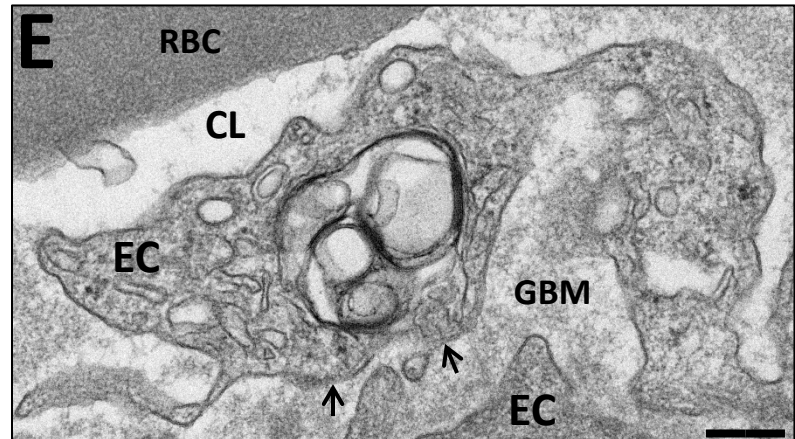

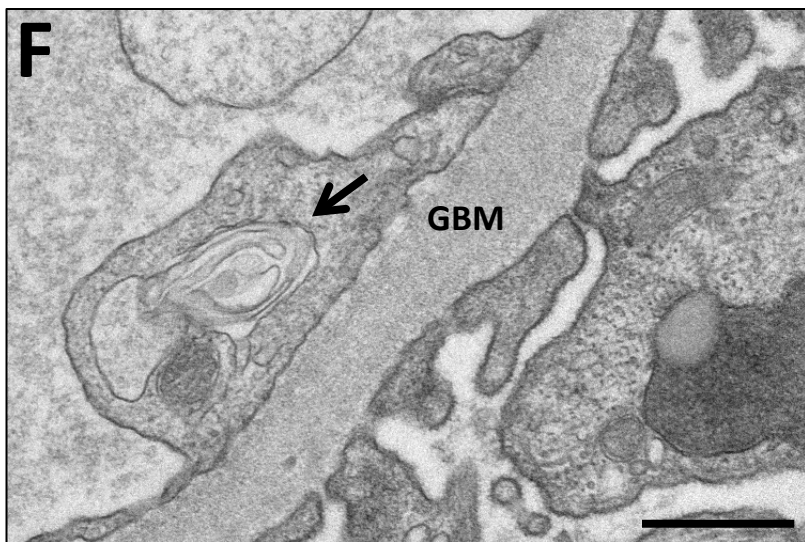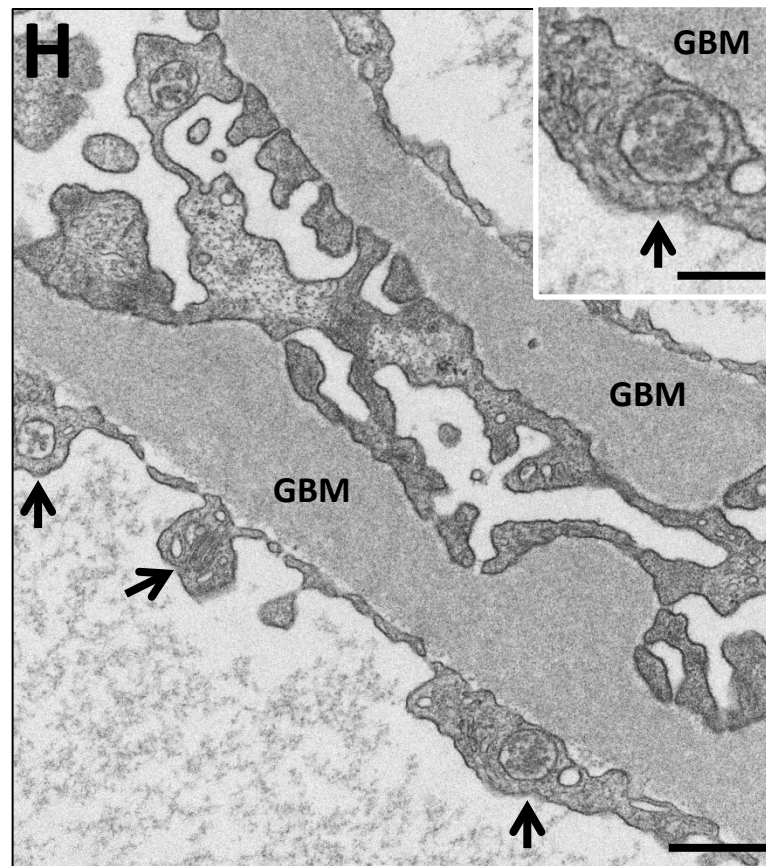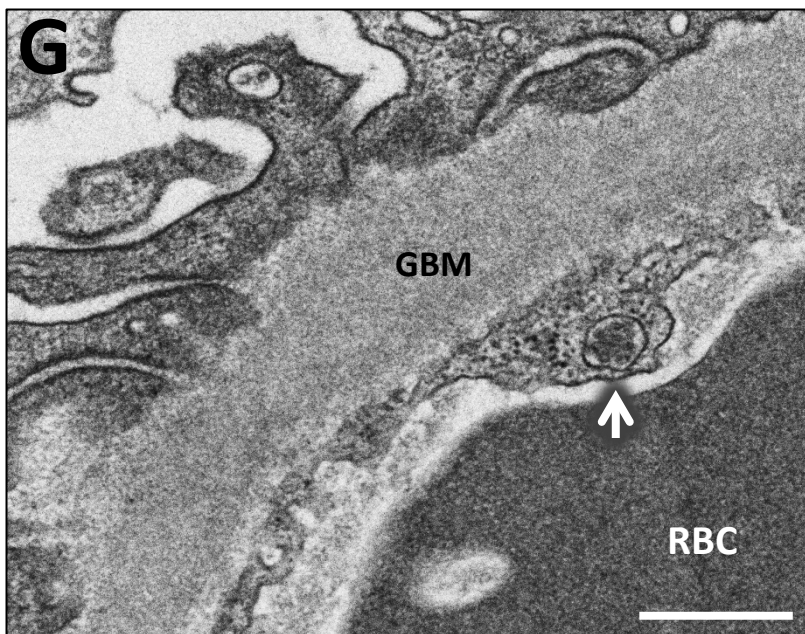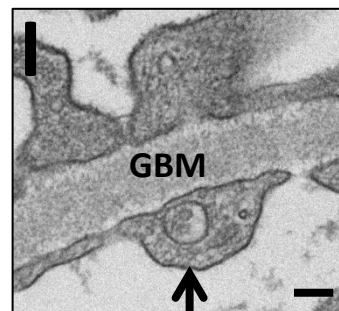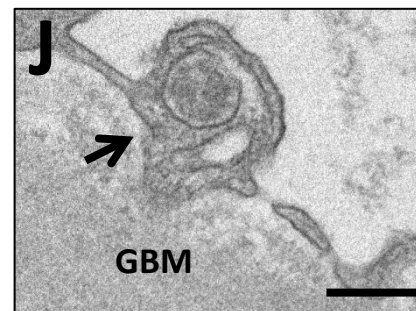

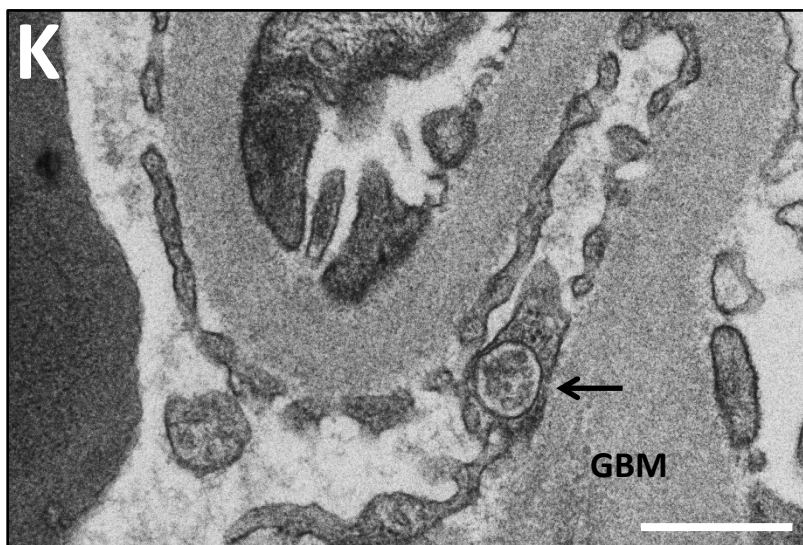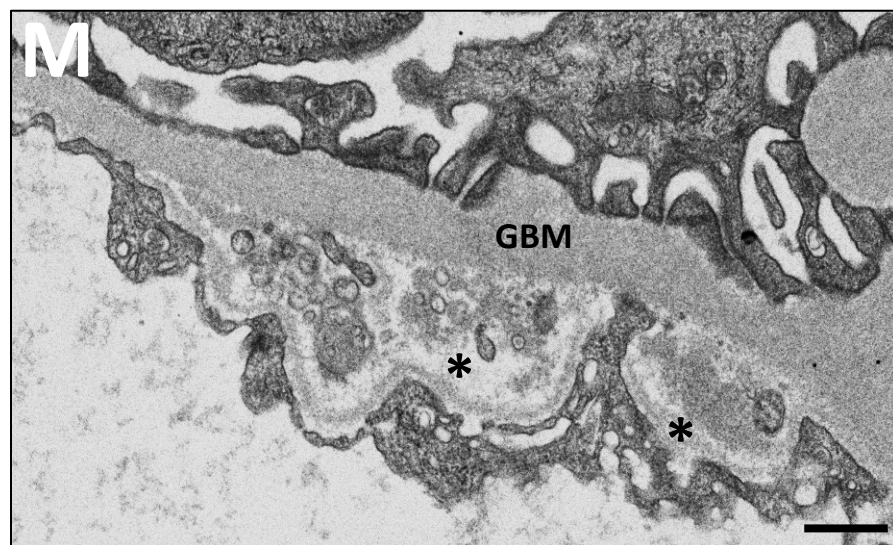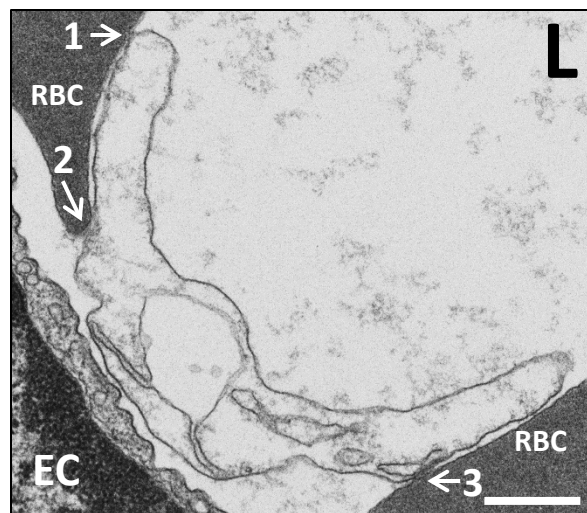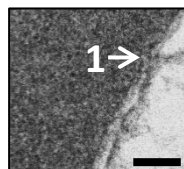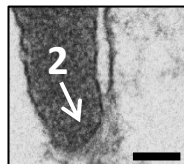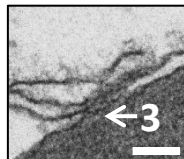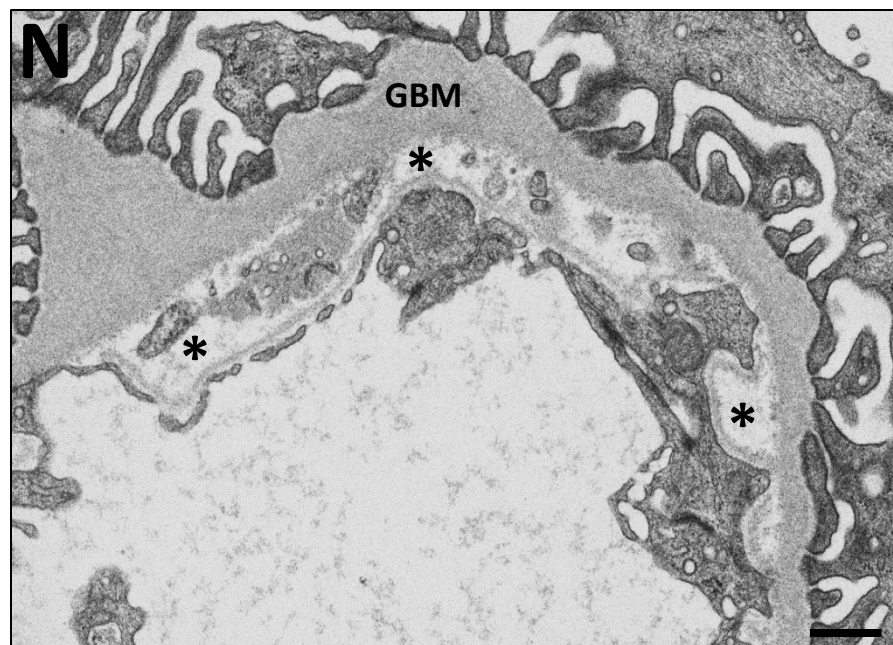

Supplement: S3 Fig — Gallery of EM images. (A) Two multiloculated LpX particles are seen bound to an endothelial cell (EC) lining a renal capillary. Note the EC surface ruffling where the LPX binds (white arrows). (B) LpX particle (arrow) binding to a renal EC lamellipodium. Inset: Higher magnification. Arrow: EC lamellipodium. (C) LpX particles bound to the EC surface (small arrows) are engulfed by lamellipodia (asterisks). (D) Multiloculated LpX particles are seen bound to the surface of an EC (large white arrow) and to an EC lamellipodium (large white arrowhead). LpX particles internalized by the EC are indicated by the small white arrows and LpX within the capillary lumen by the small black arrows. (E) Renal capillary EC engulfment of LpX particles. The ends of the EC lamellipodia engulfing the LpX particles are indicated by the small black arrows. (F) LpX engulfed by an EC is indicated by the black arrow. (G-K) EC lysosomes containing degraded LpX pariticles (arrows). Inset in (H): Higher magnification of lowermost EC. (L) A partially degraded LpX particle in a renal capillary lumen is bound to, and bridging two RBCs. Insets: Higher magnification of LpX contact points with RBCs. (M,N) Matrix degradation (indicated by asterisks) in GBM in regions containing numerous LpX particles. Scale bars: I = 100nm; L1,2 = 125 nm; A, D, E = 200 nm. H, H inset, B inset, J = 250nm; B, C, F-H, K-N = 500 nm. (PDF) [file pone.0150083.s003.pdf]

# S4

**Macropinocytotic uptake of LpX by *Lcat*<sup>-/-</sup>  
renal glomerular endothelial cells**

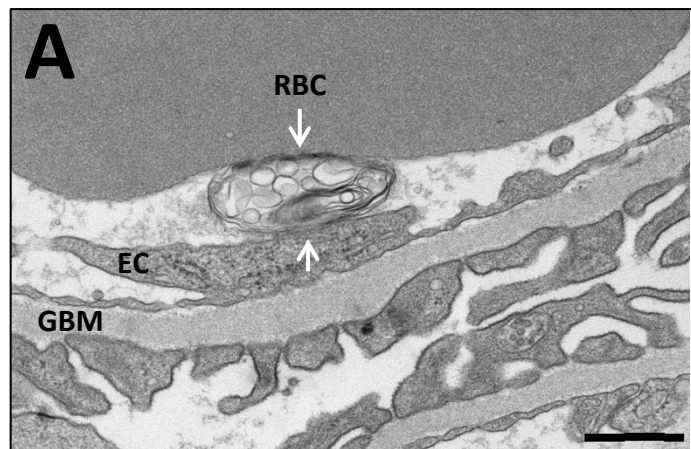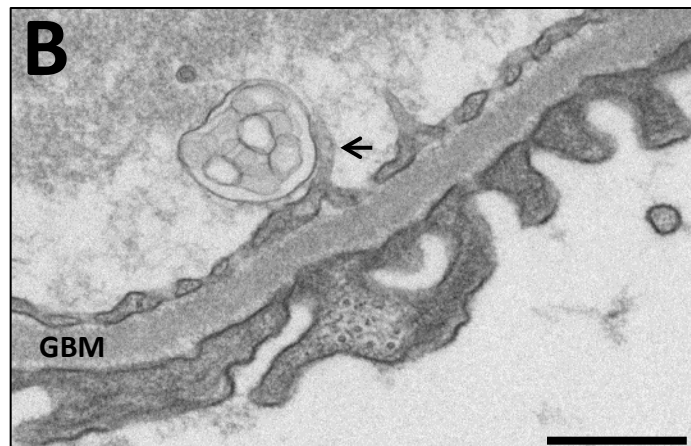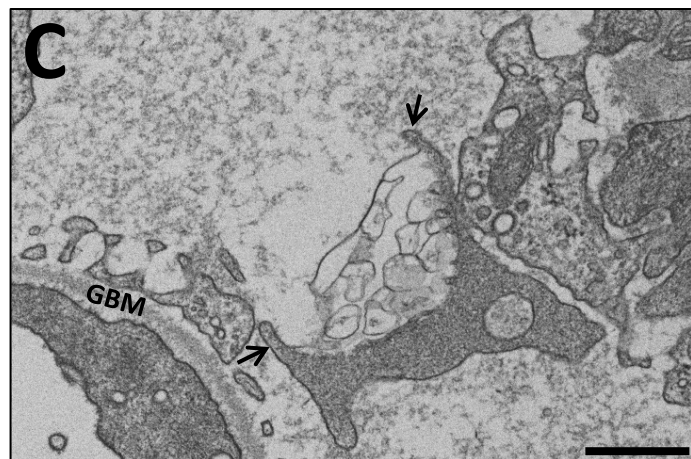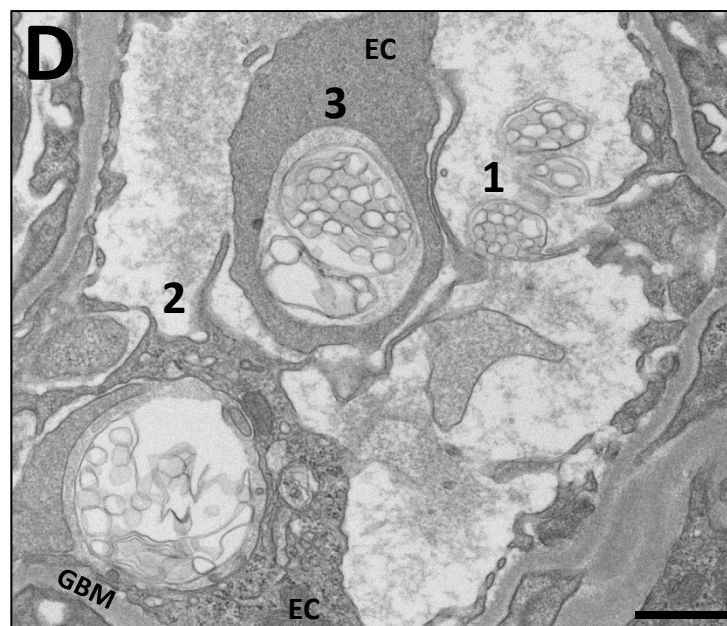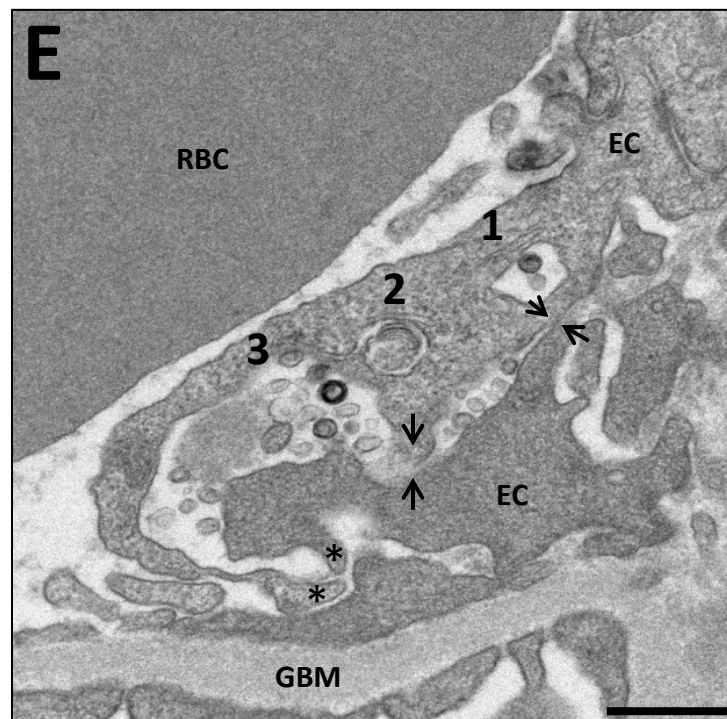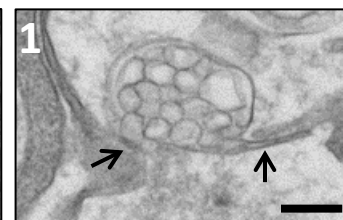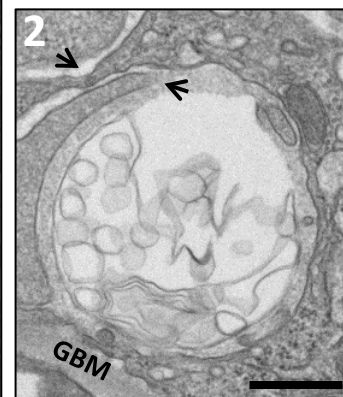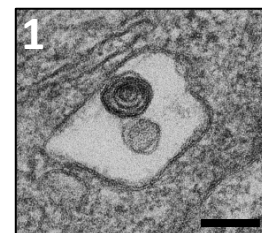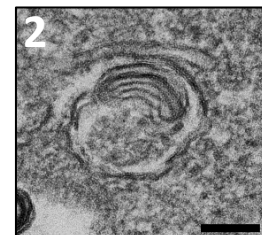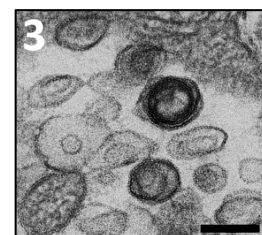

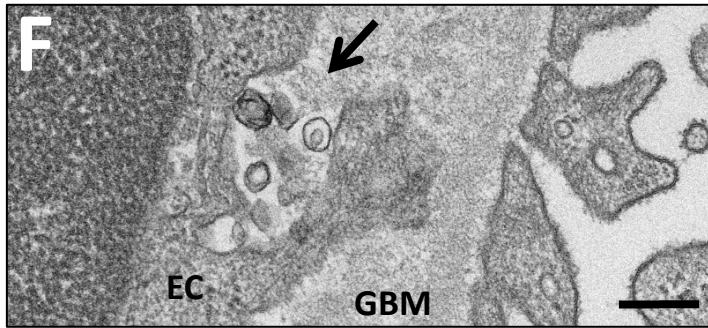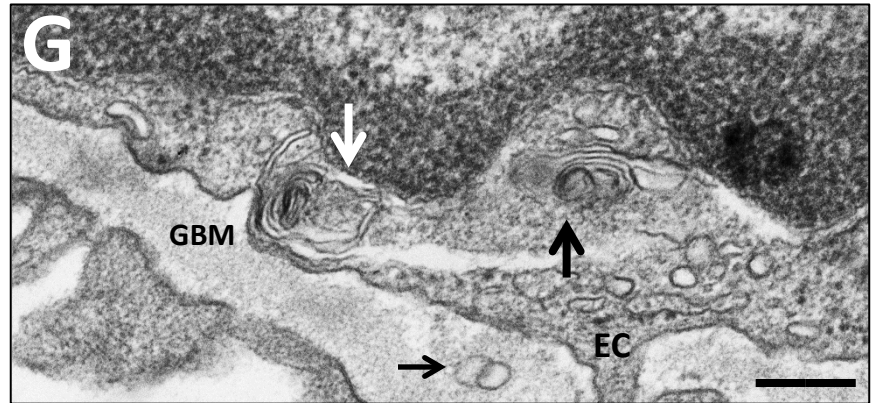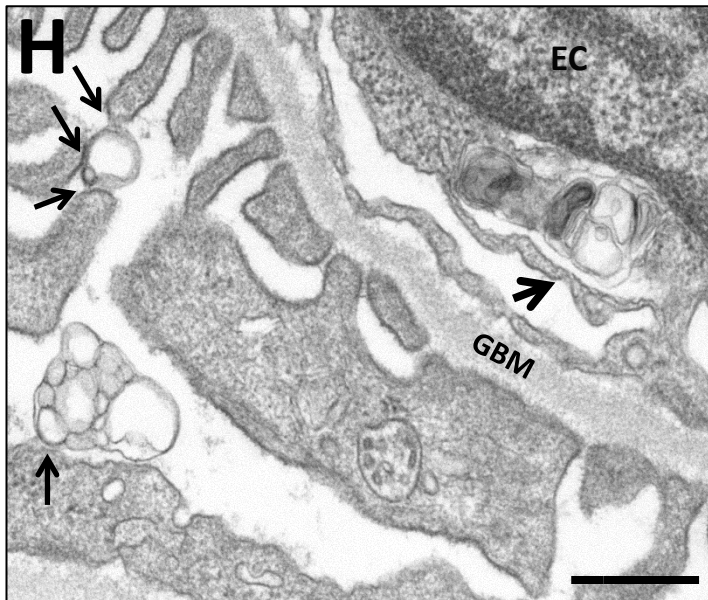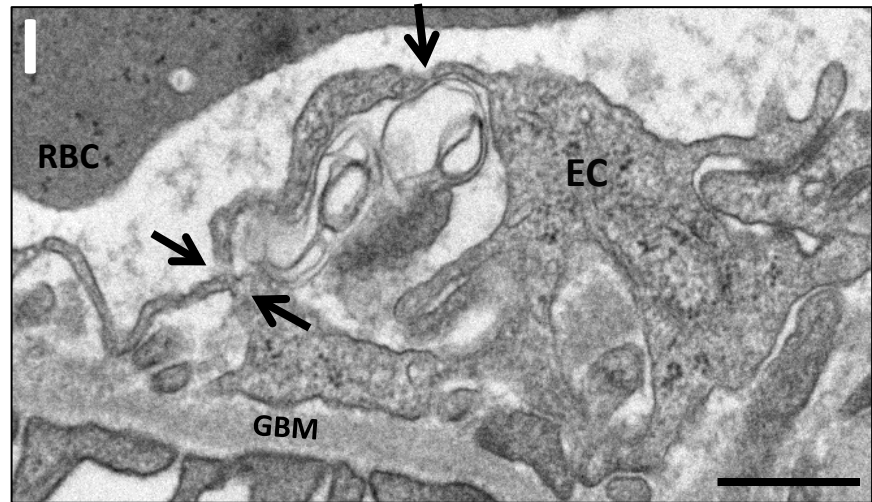

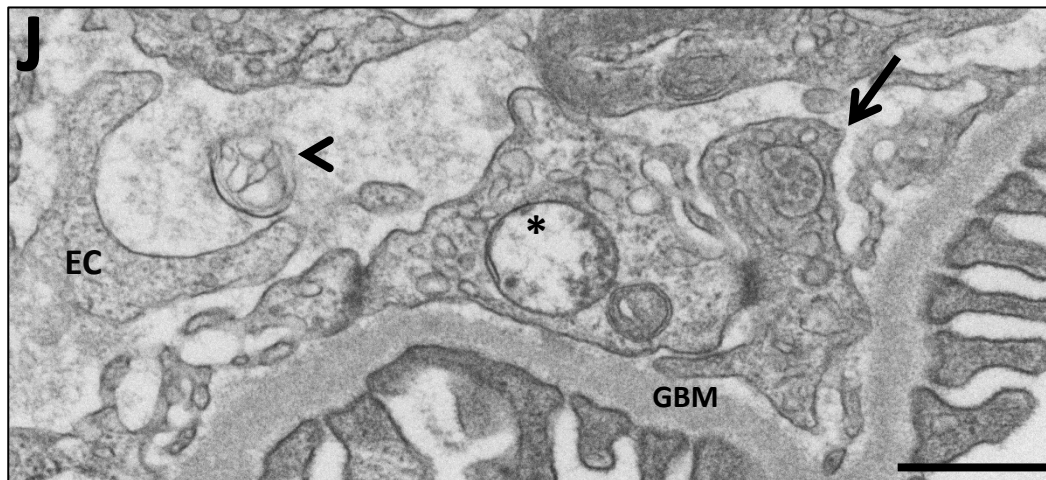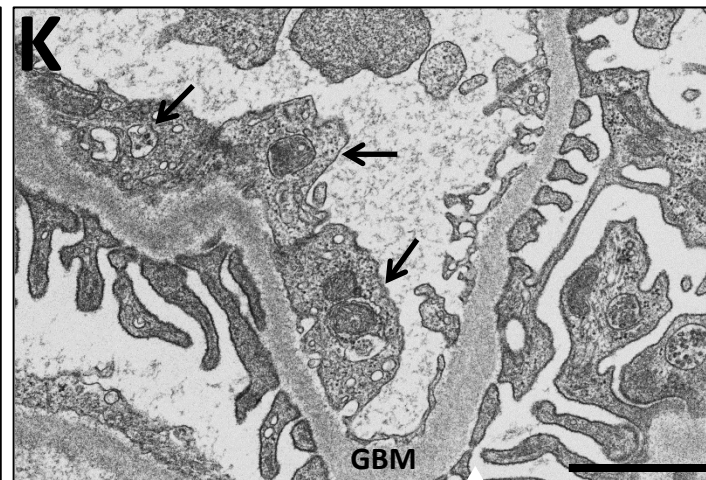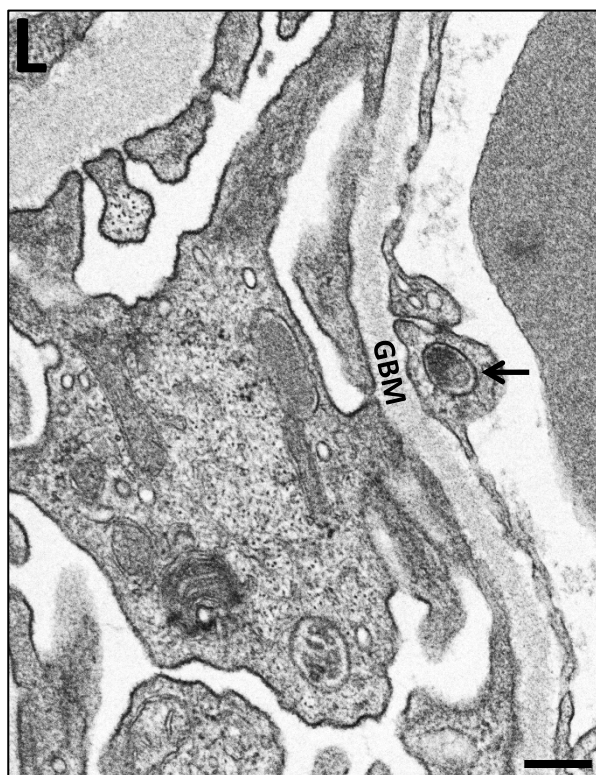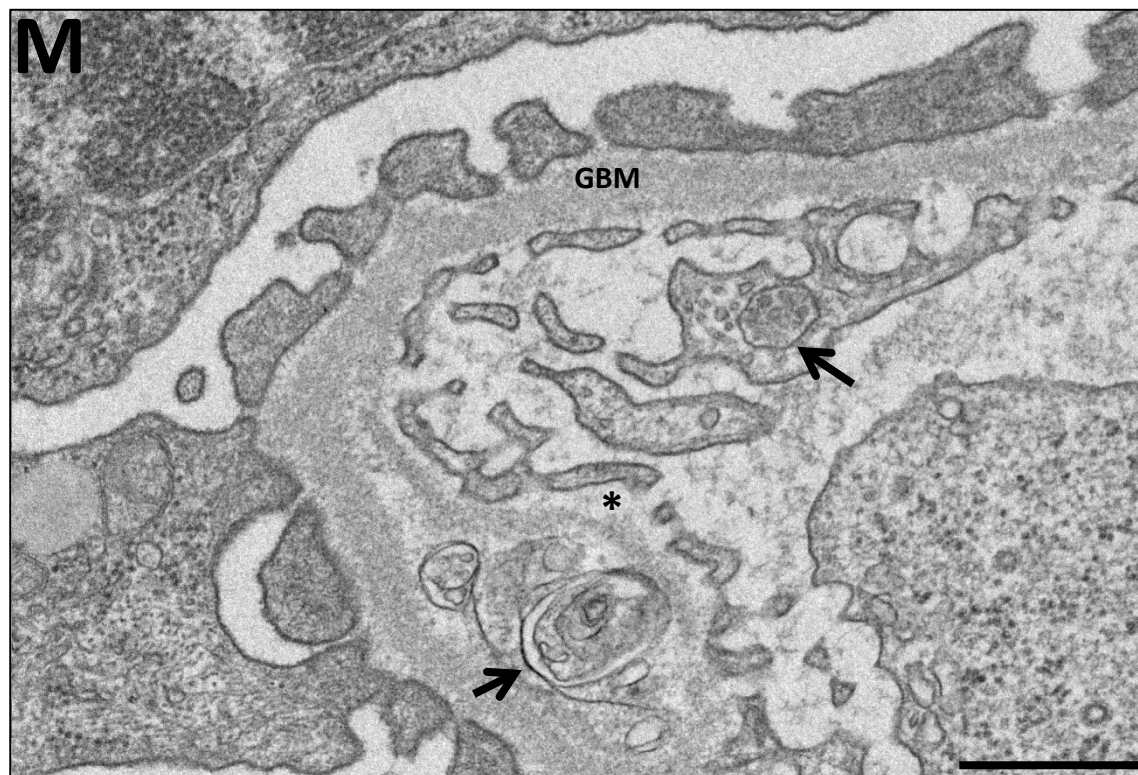

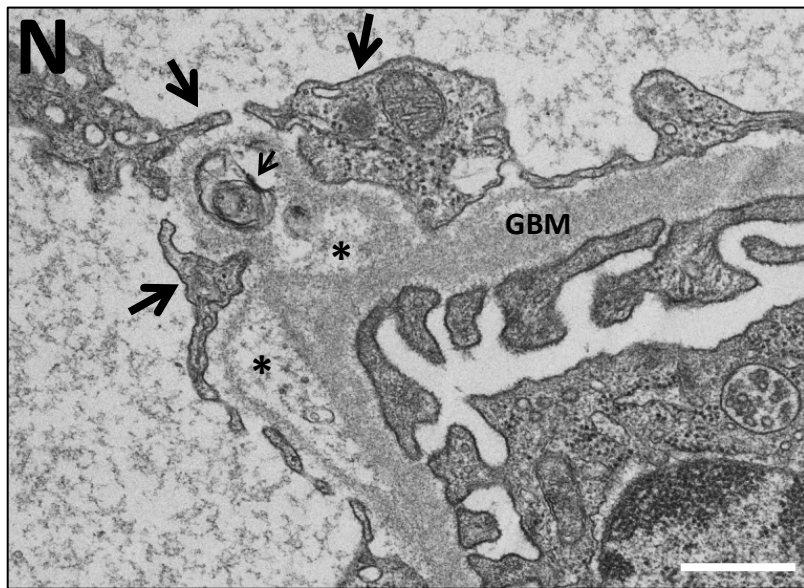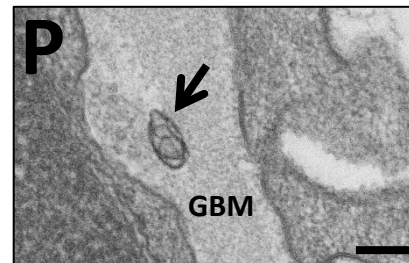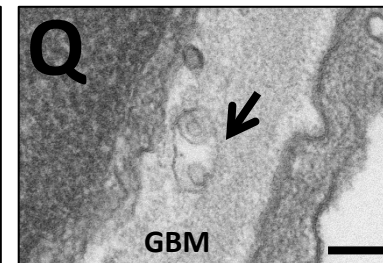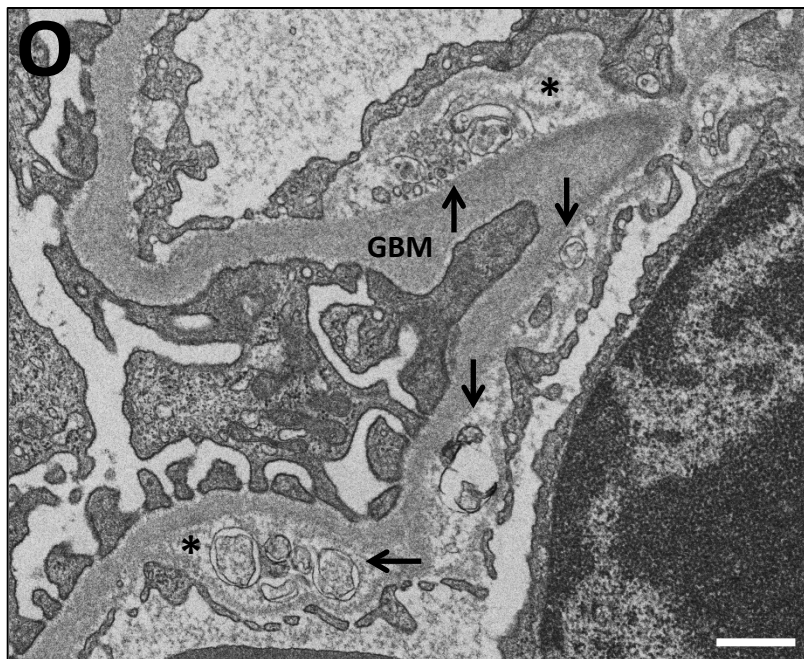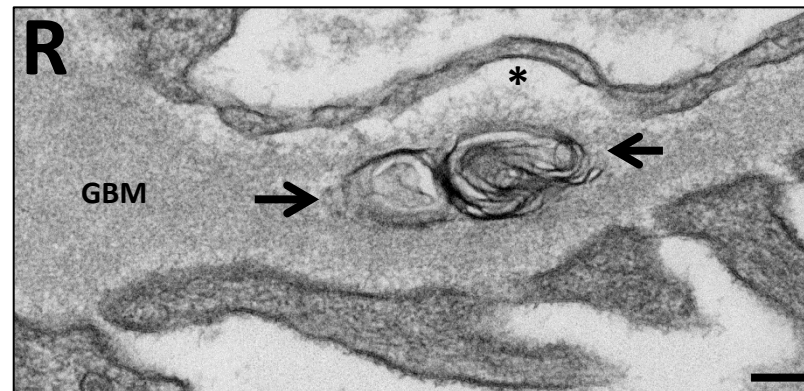

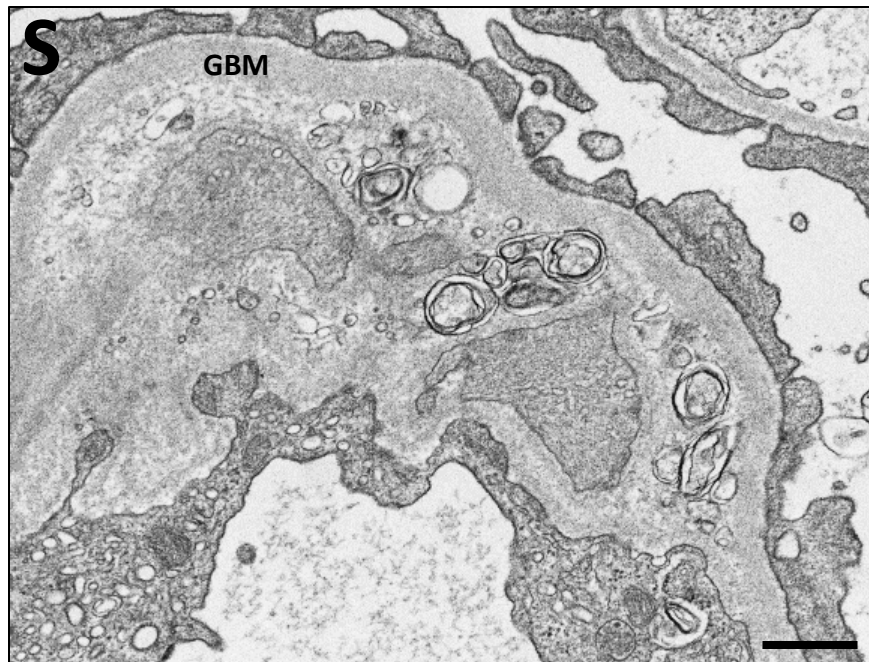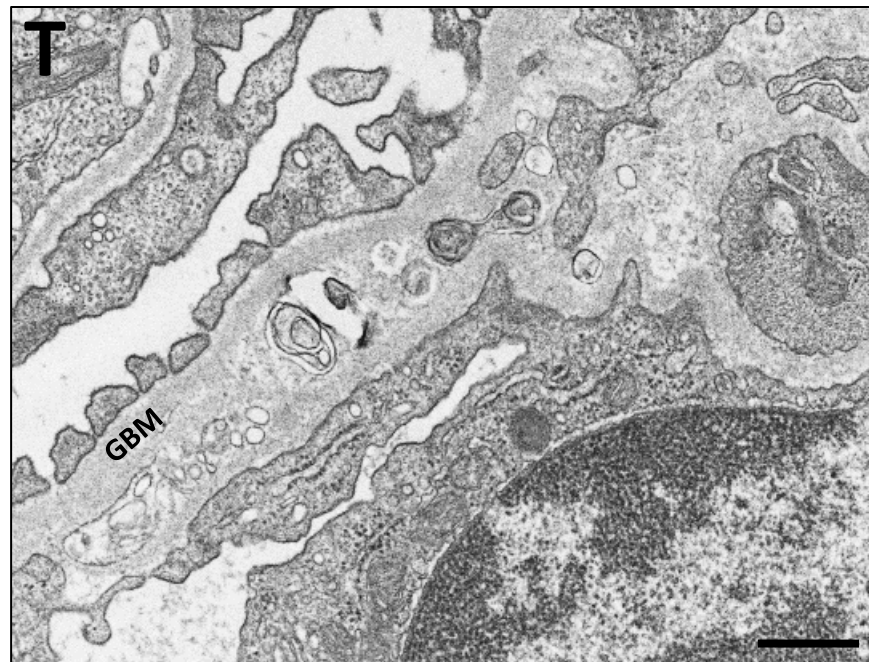

Supplement: S4 Fig — Gallery of EM images. (A) A multiloculated LpX particle bound to an RBC (upper arrow) also binds to the surface of an EC (lower arrow) lining a renal capillary. (B) An EC lamellipodium (arrow) binds an LpX particle. (C) An EC binding an LpX particle extends lamellipodia (arrows), initiating LpX internalization. (D) Enlarged version of Fig 3B. (E) Upper EC with LpX-containing vacuoles (1,2,3) extends a lamellipodium (on left) in close proximity to a lamellipodium extended by the lower EC (asterisks), enclosing numerous LpX particles (E3). The upper and lower ECs appear to have fused at points of contact (arrows), enclosing several LpX particles. (F) An EC extends a lamellipodium into the GBM enclosing several LpX particles (arrow). (G) Large multilamellar LpX particles (large arrows) internalized by an EC as well as small LpX particles infiltrating the GBM (small arrows) are seen. (H) EC lamellipodium (large arrow) encloses LpX particles within an EC. Note LpX in the urinary space binds to PCB and PFPs (small arrows). (I) EC lamellipodium fuses with EC body at points of contact (arrows), enclosing LpX particles. (J) EC lamellipodia encircle an LpX particle (arrowhead). Note phagolysosome (asterisk) and multivesicular body (MVB) (arrow) containing dergraded LpX. (K,L) Phagolysosomes (arrows) containing degraded LpX in renal capillary endothelium. (M) EC contains MVB with LpX remnants (large arrow). LpX particles penetrate the GBM (small arrow), disrupting the GBM matrix (asterisk). (N) Fig 3D enlarged. (O) Numerous LpX particles penetrate the GBM (arrows), disrupting the GBM matrix (asterisks). (P-T) LpX particles penetrate the GBM (arrows), disrupting the GBM matrix. Scale bars: E1, E2, E3, P, R = 100 nm; F, G = 200 nm; D1 = 250 nm; H = 400 nm; A-D, D2, E, I–O, S, T = 500 nm. (PDF) [file pone.0150083.s004.pdf]

**S5**

**WT mouse glomerular podocyte  
LpX uptake**

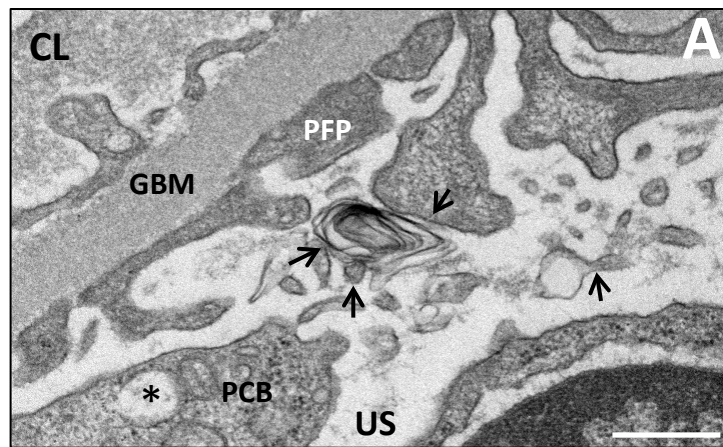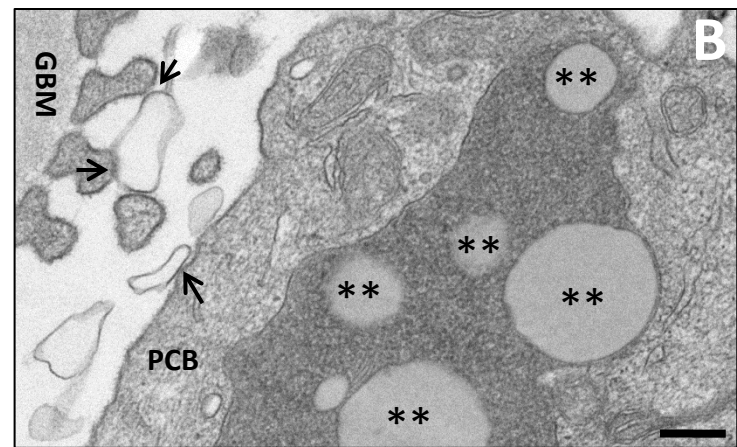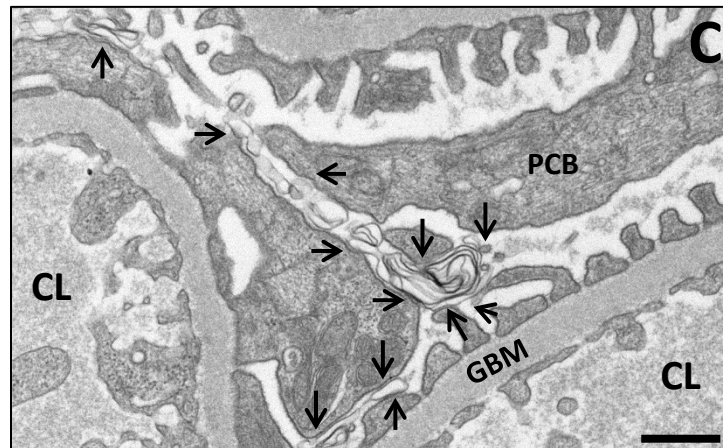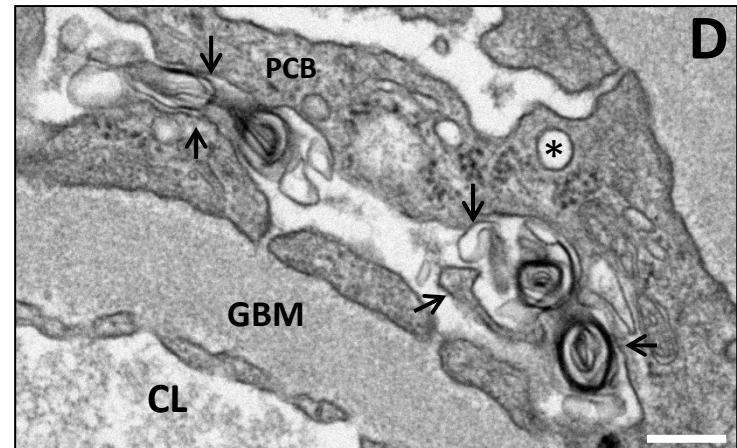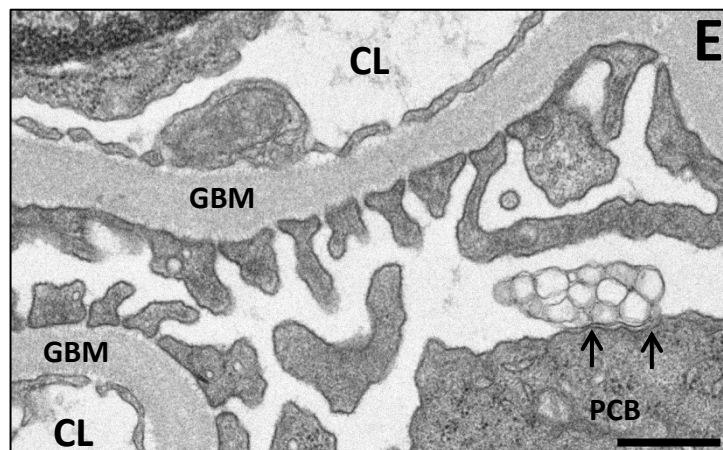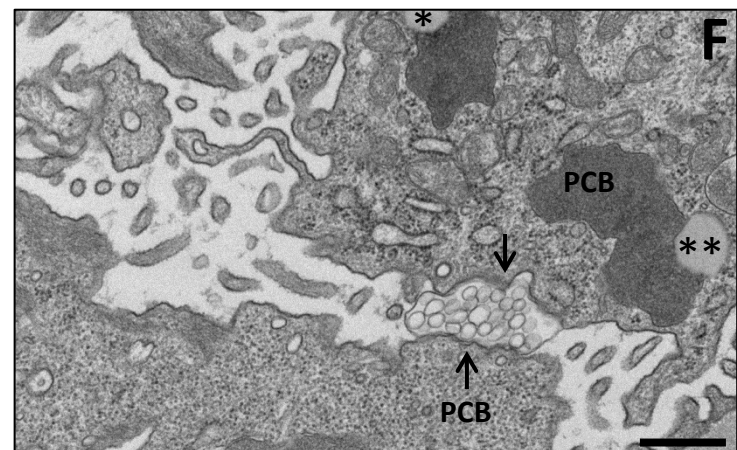

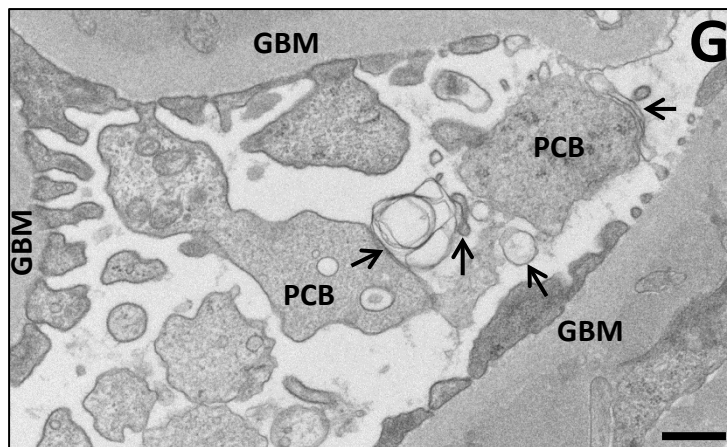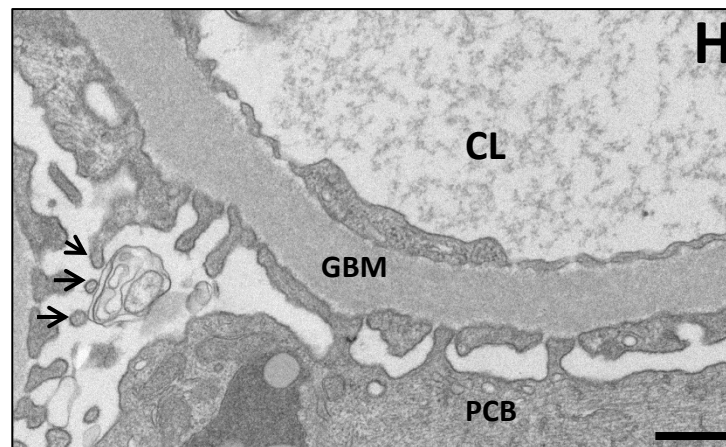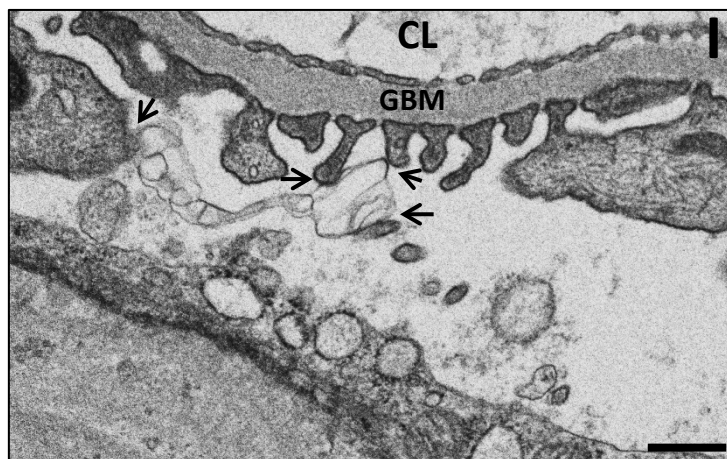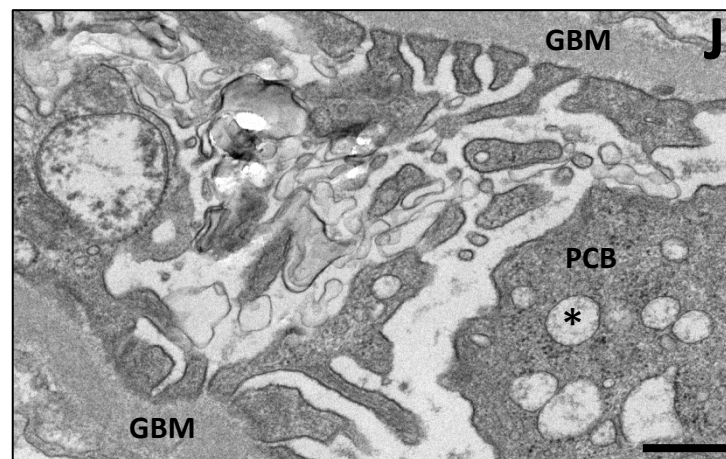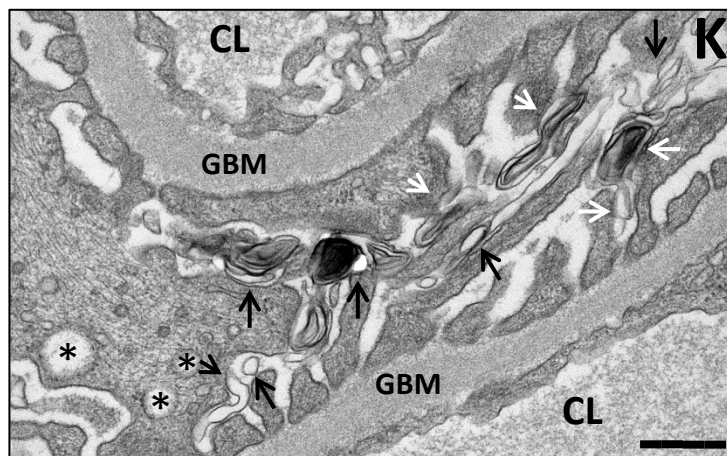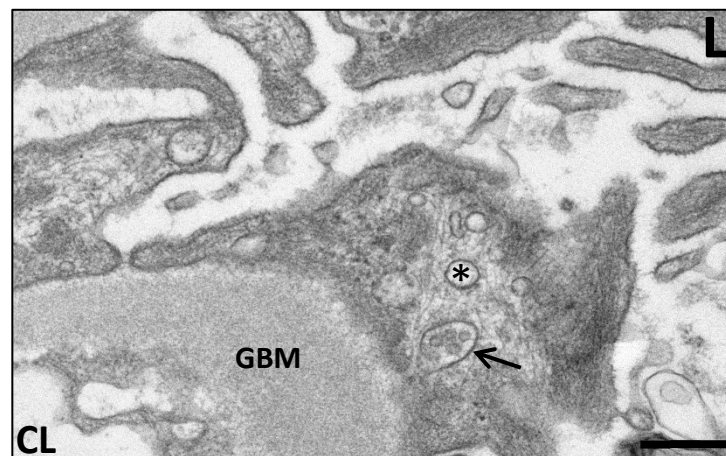

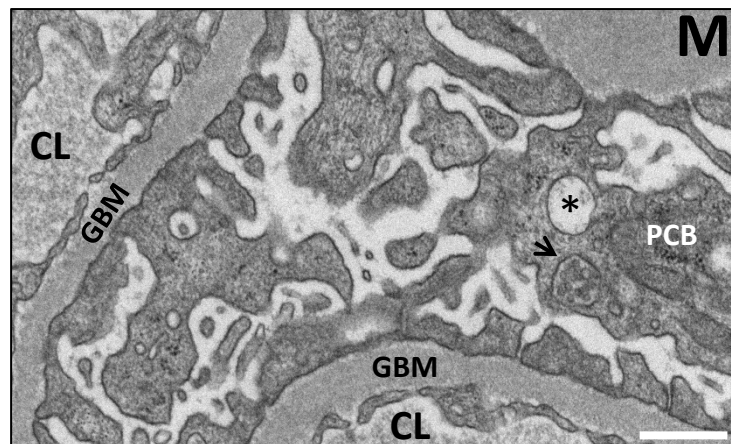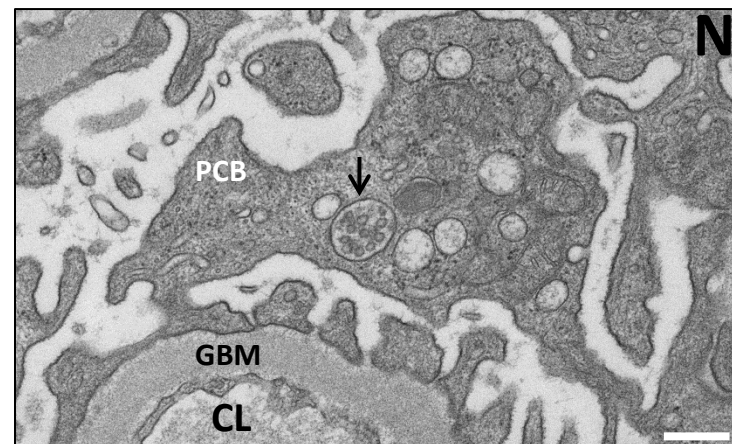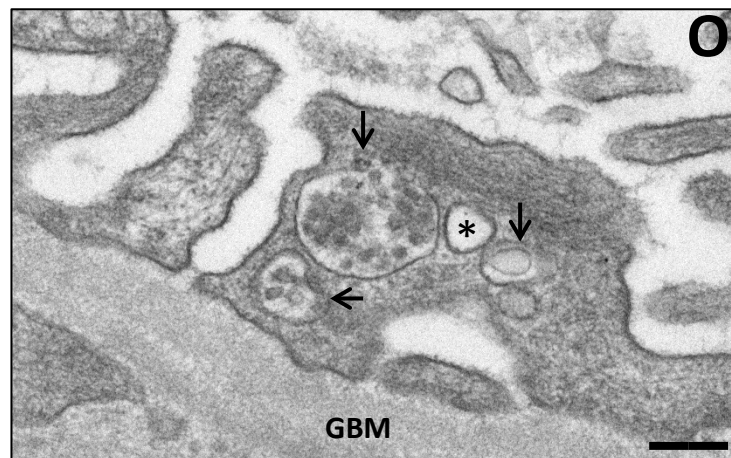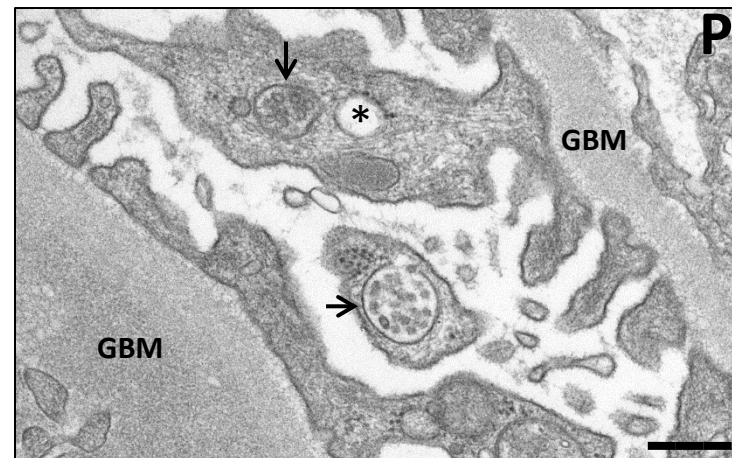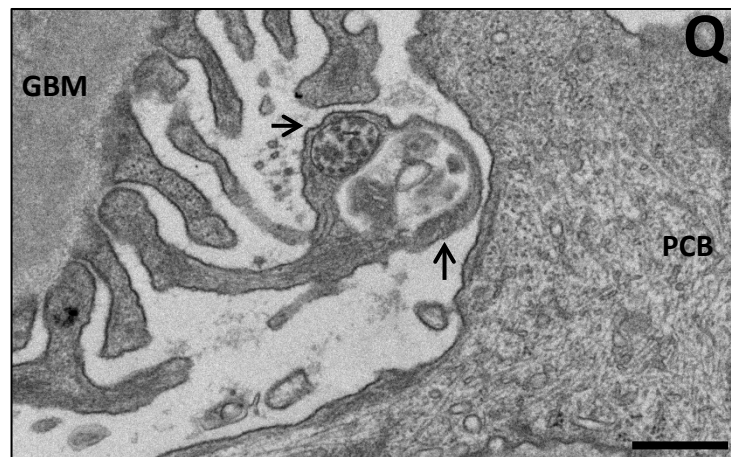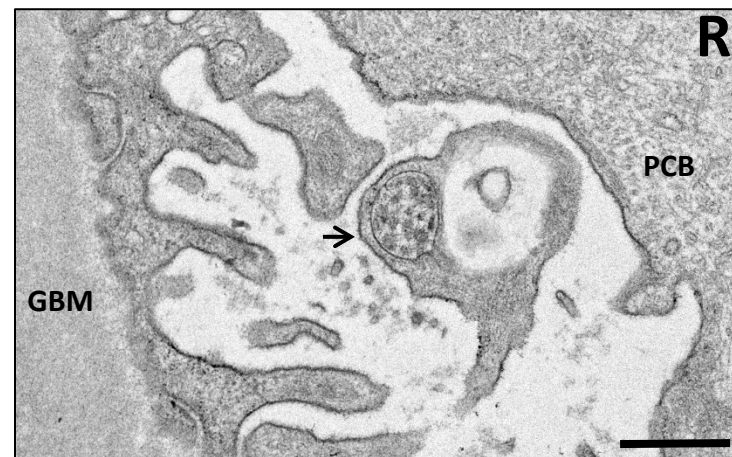

Supplement: S5 Fig — Gallery of EM images. (A-K) LpX particles bind to PCBs and PFPs (arrows) in the urinary space (US; indicated in (A)). Double asterisks in (B, F) denote large lipid droplets within a PCB, and, single asterisks denote representative vacuoles seen in PCBs (D,J,M-P). Note the membrane ruffling at sites of LpX binding in (E,F). (L-R) PCBs and PFPs also often contain multivesicular bodies (MVB) with numerous small unilamellar vesicles (black arrows). (O,Q,R) Larger MVBs were also seen to contain partially degraded LpX particles. CL = capillary lumen; GBM = glomerular basement membrane. Scale bars: B, D, O = 200 nm; L, N, P = 300 nm; K = 400 nm; A, C, E–J, M, Q, R = 500 nm. (PDF) [file pone.0150083.s005.pdf]

**S6**

***Lcat*<sup>-/-</sup> mouse glomerular podocyte  
LpX uptake**

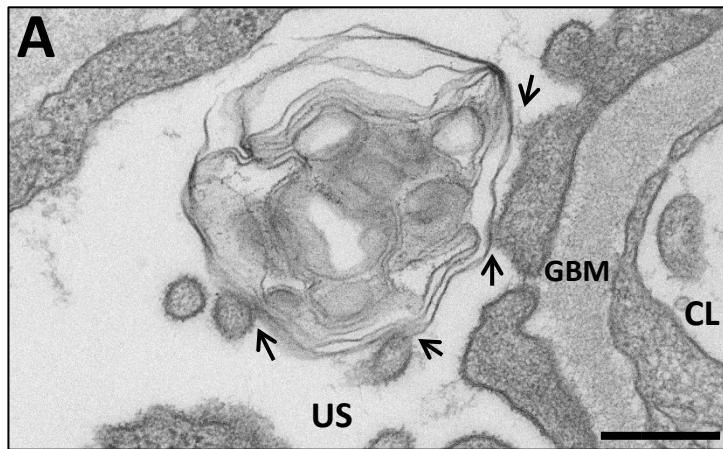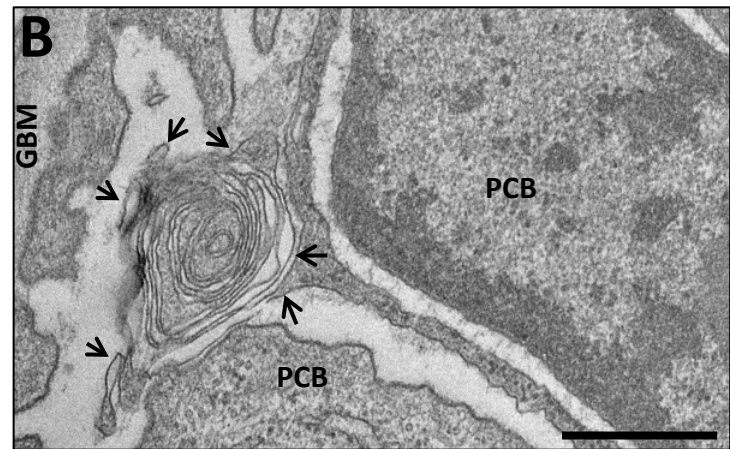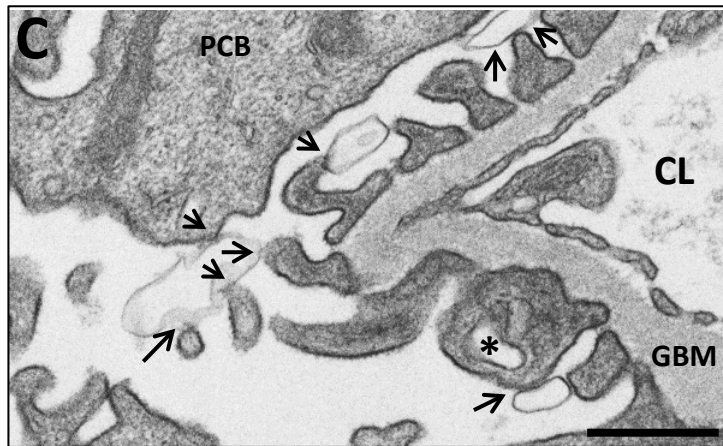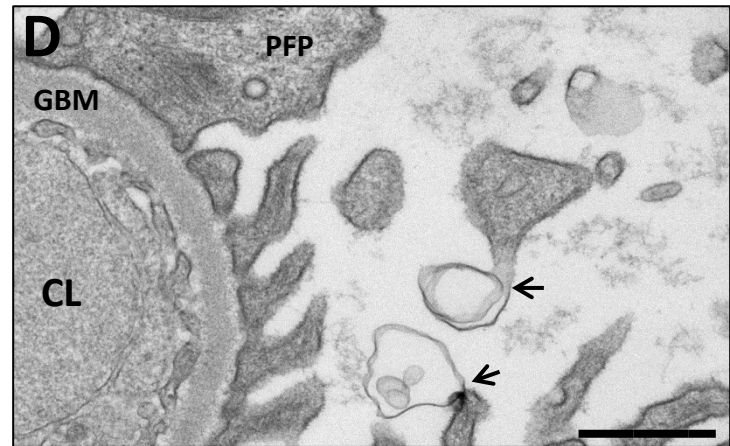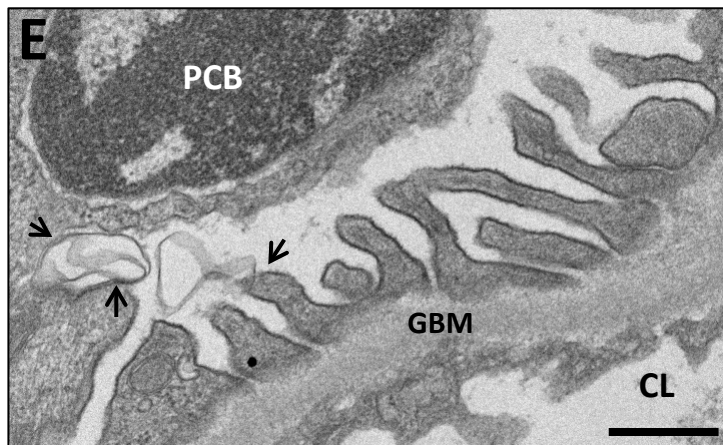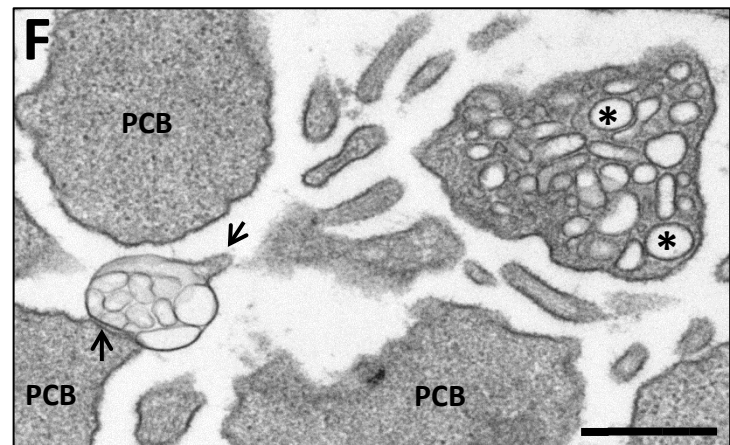

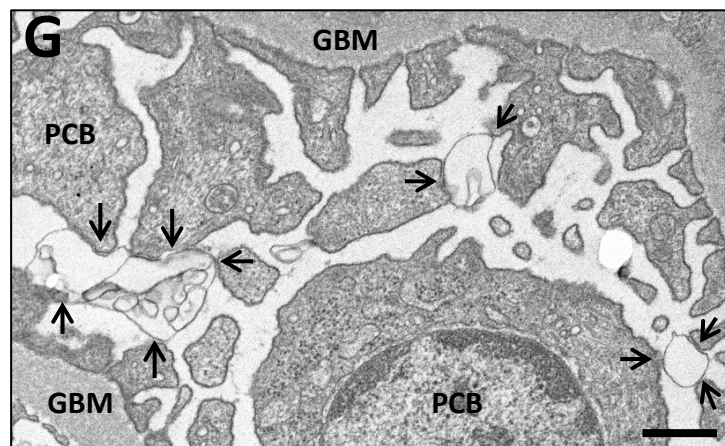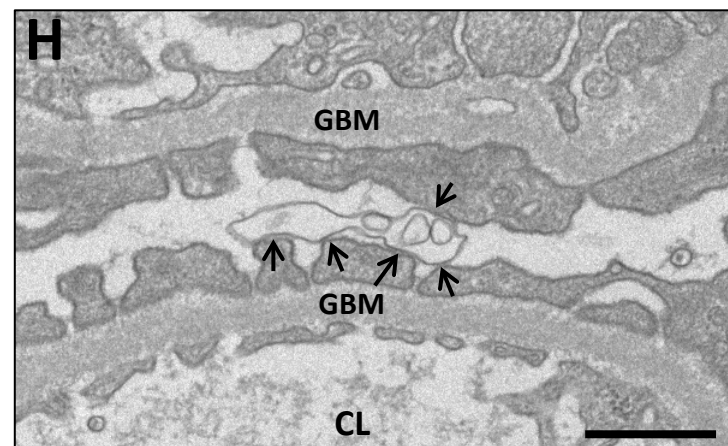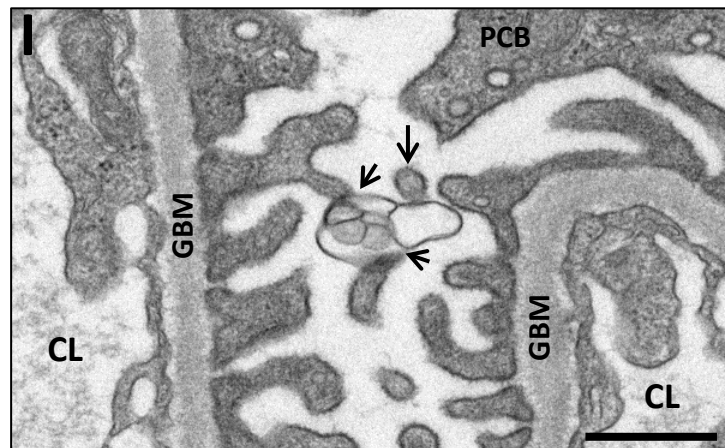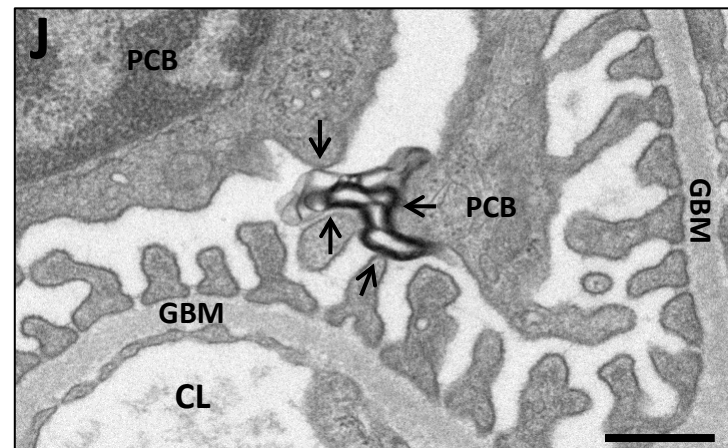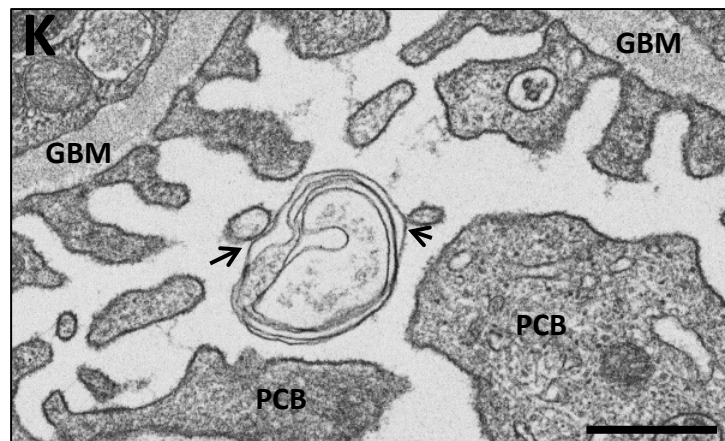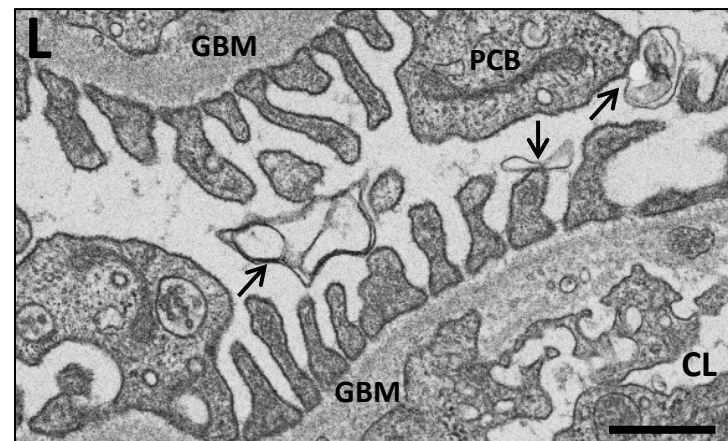

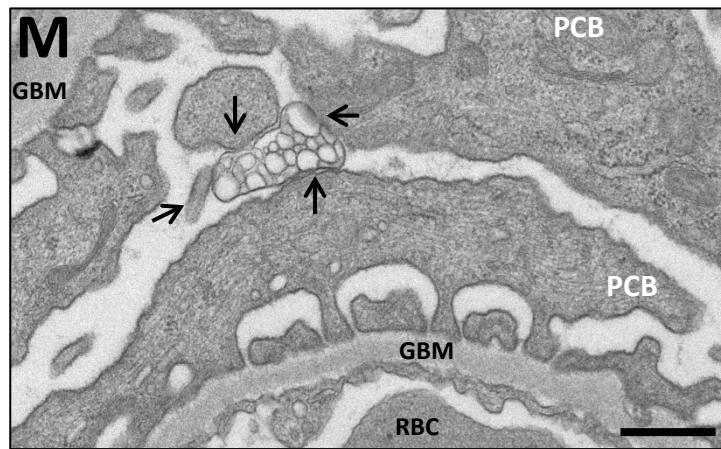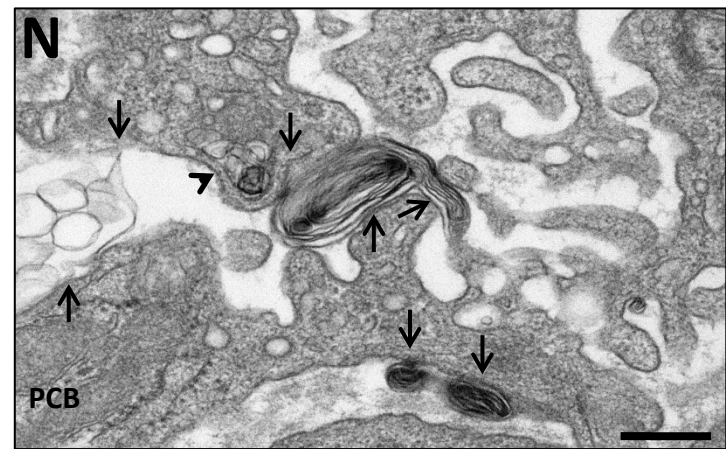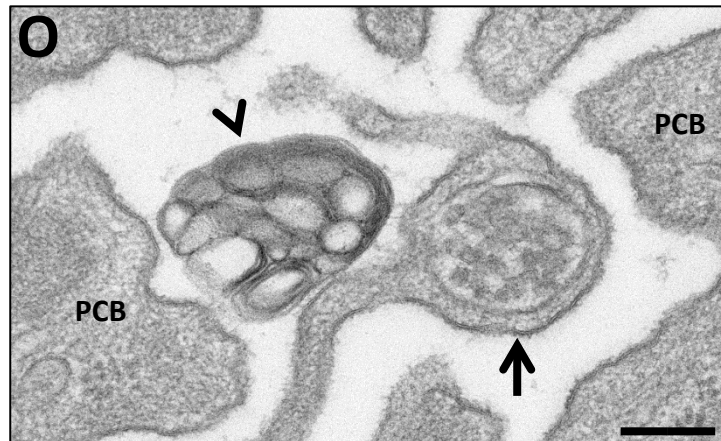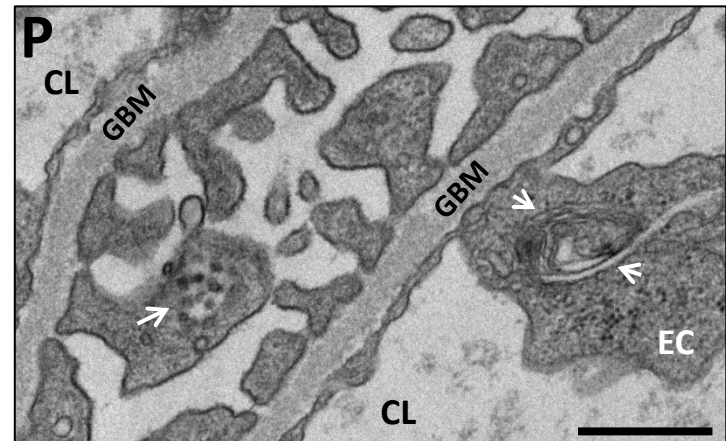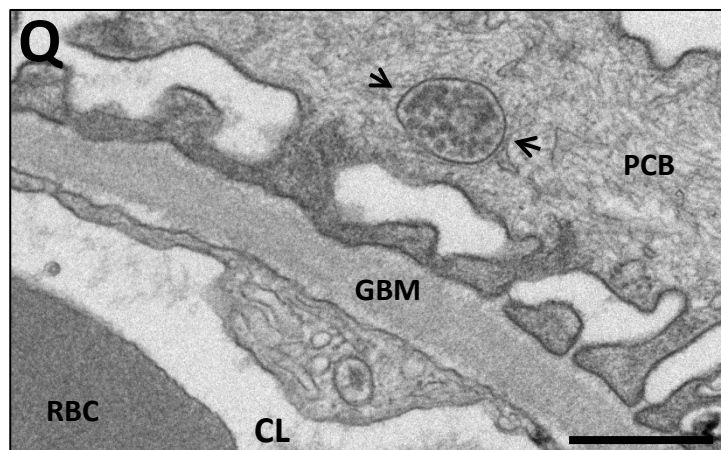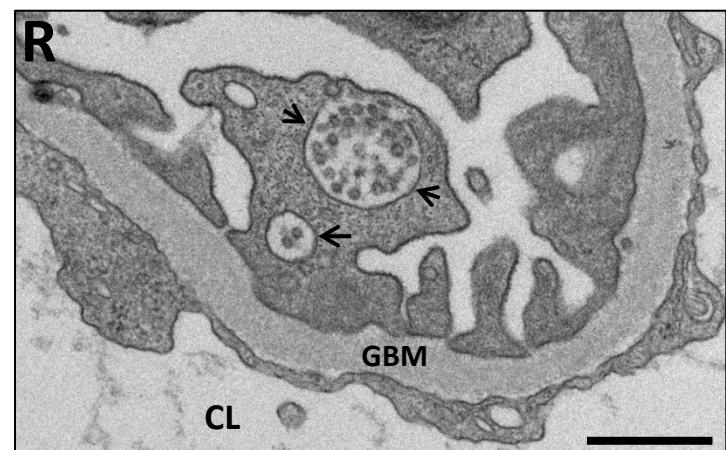

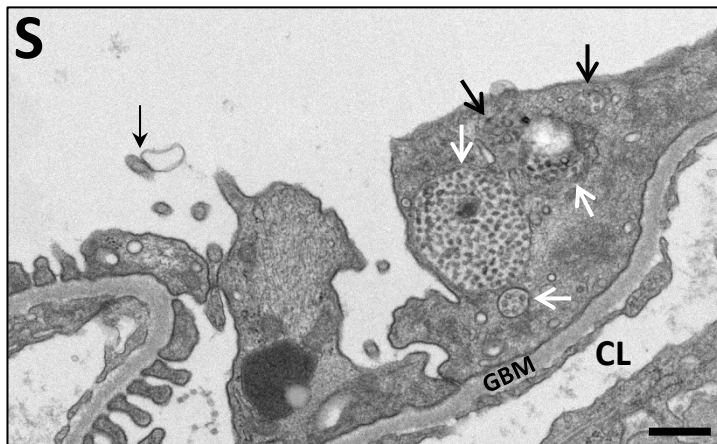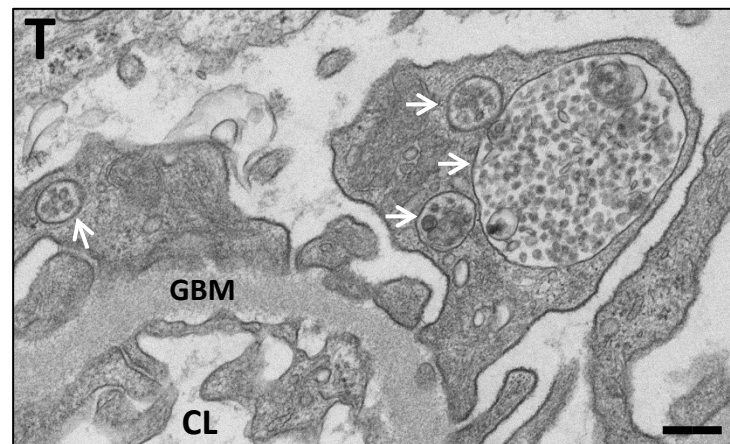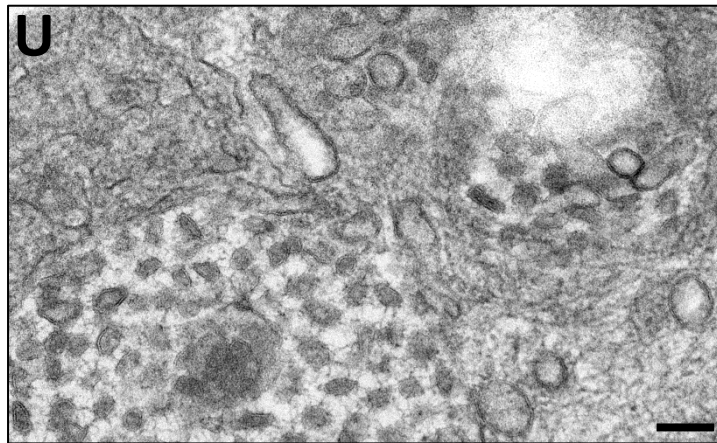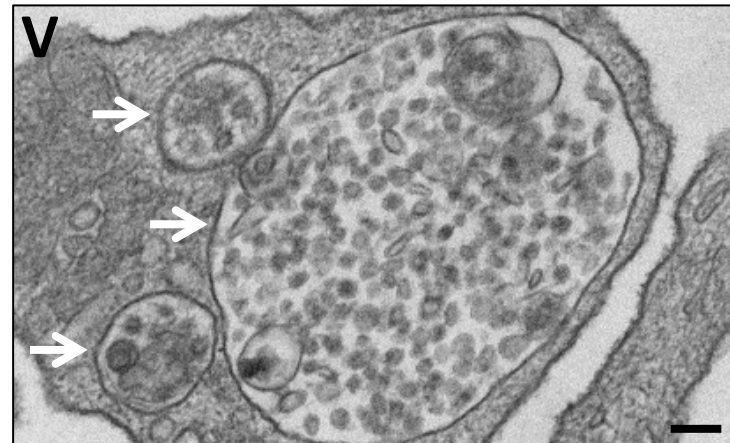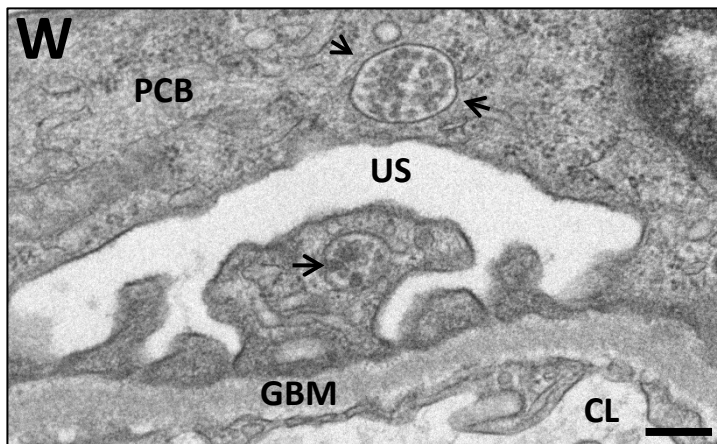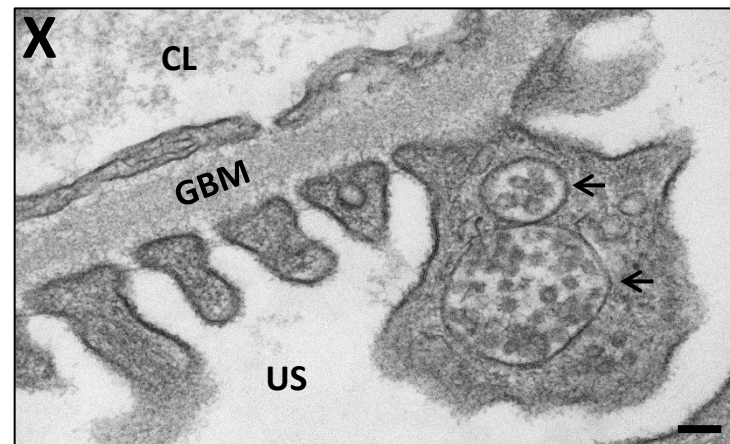

Supplement: S6 Fig — Gallery of EM images. (A-N) LpX particles bind to PCBs and PFPs (arrows) in the urinary space (US; indicated in (A)). (C,F) Asterisks denote representative vacuoles seen in PCBs. A multilamellar LpX particle in a phagosome is indicated by the arrowhead in (N). (O-W) LpX degradation in podocyte phagolysosomes. MVBs containing small unilamellar vesicles as well as large phagolysosomes containing both partially degraded LpX particles as well as unilamellar vesicles are indicated by white and black and arrows, respectively. LpX particles in the urinary space are indicated by the arrowhead in (O) and small arrow in (S). (U) and (V) are enlargements of (S) and (T), respectively. Note the large phagosome containing a large multilamellar LpX particle in a renal capillary EC in (P) (small white arrows). Scale bars: U, V, X = 100 nm; A, O, T = 200 nm; W = 250 nm; N = 300 nm; B–M, P-S = 500 nm. (PDF) [file pone.0150083.s006.pdf]

**S7**

**LpX movement into WT mouse  
glomerular mesangium**

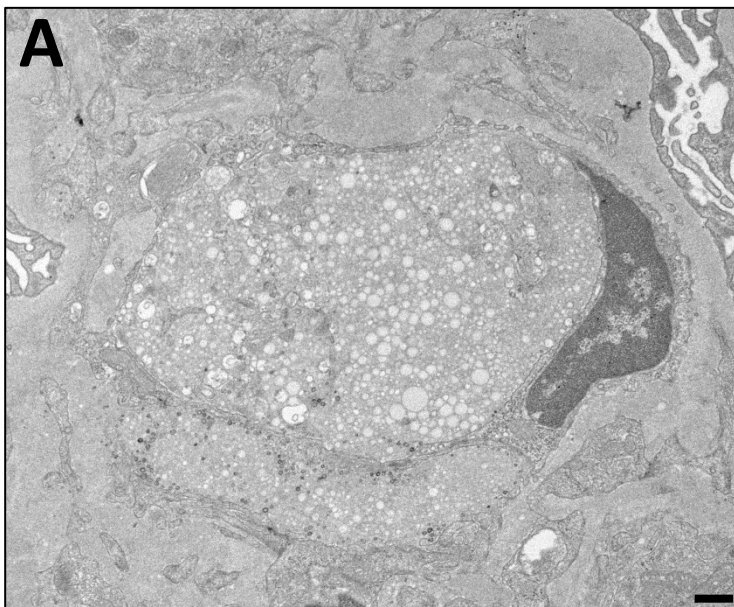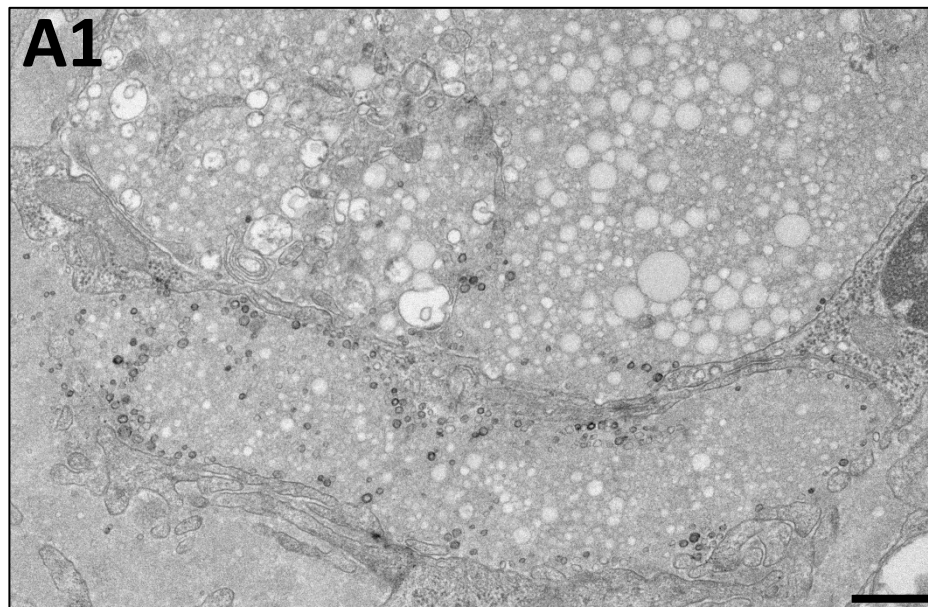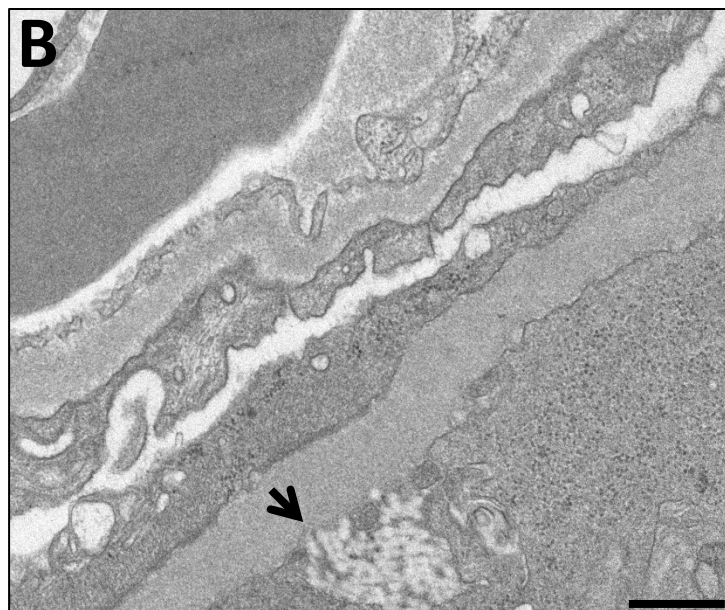

Supplement: S7 Fig — Gallery of EM images. Lpid deposition in mesangial cells (MC) and matrix (MM). (A) Foamy MC containing numerous fat droplets. (A1) Higher magnification of the cell in (A). (B) Fat deposition in MM (black arrow). Scale bars: 500 nm. (PDF) [file pone.0150083.s007.pdf]

**S8**

**LpX movement into *Lcat*<sup>-/-</sup> mouse  
glomerular mesangium**

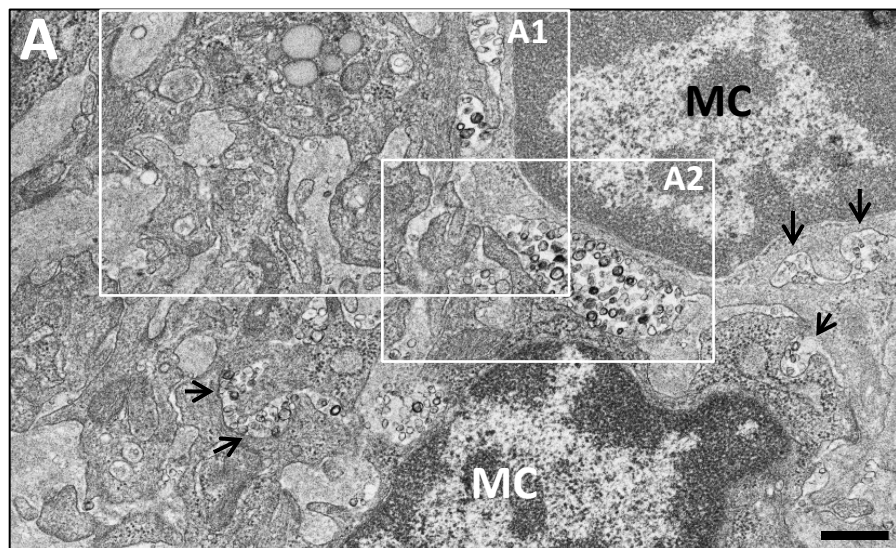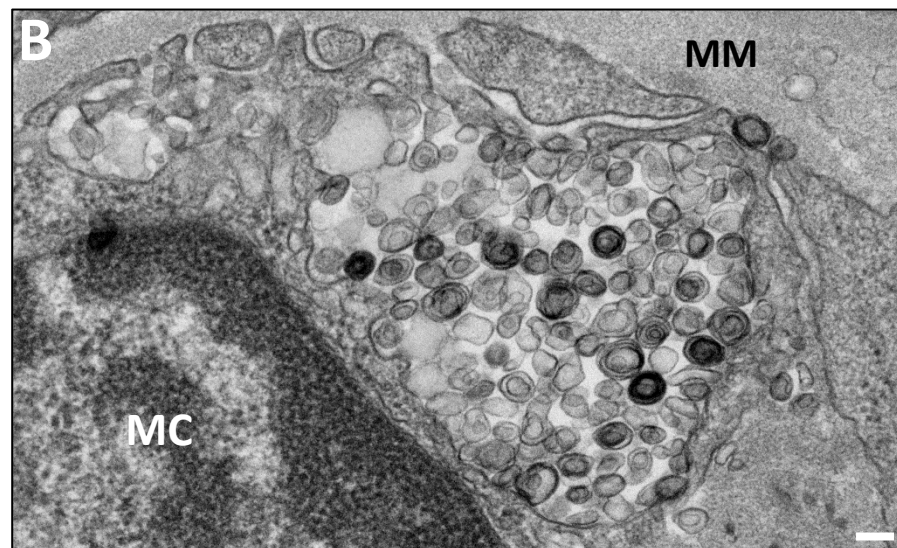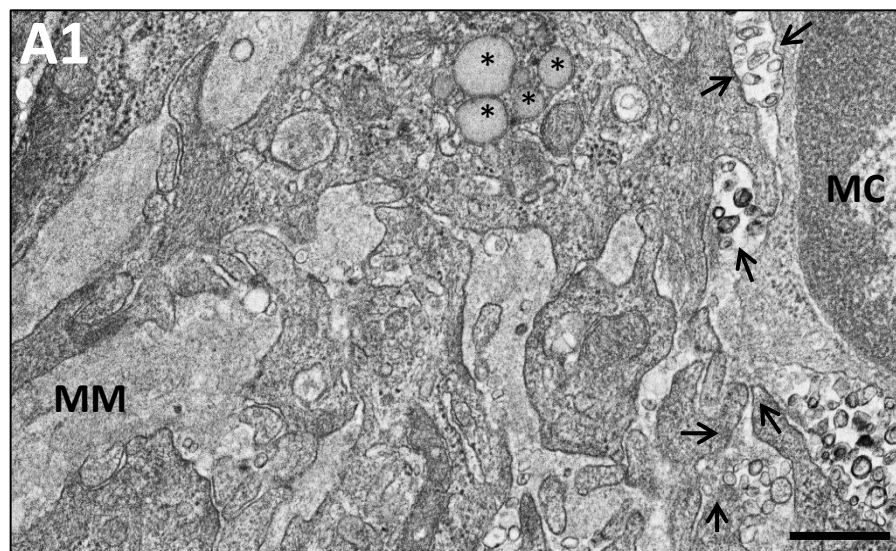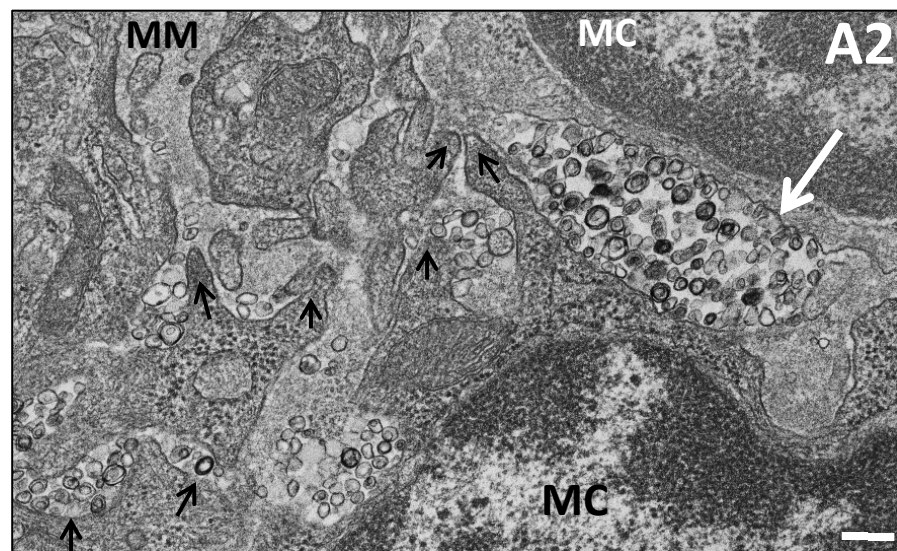



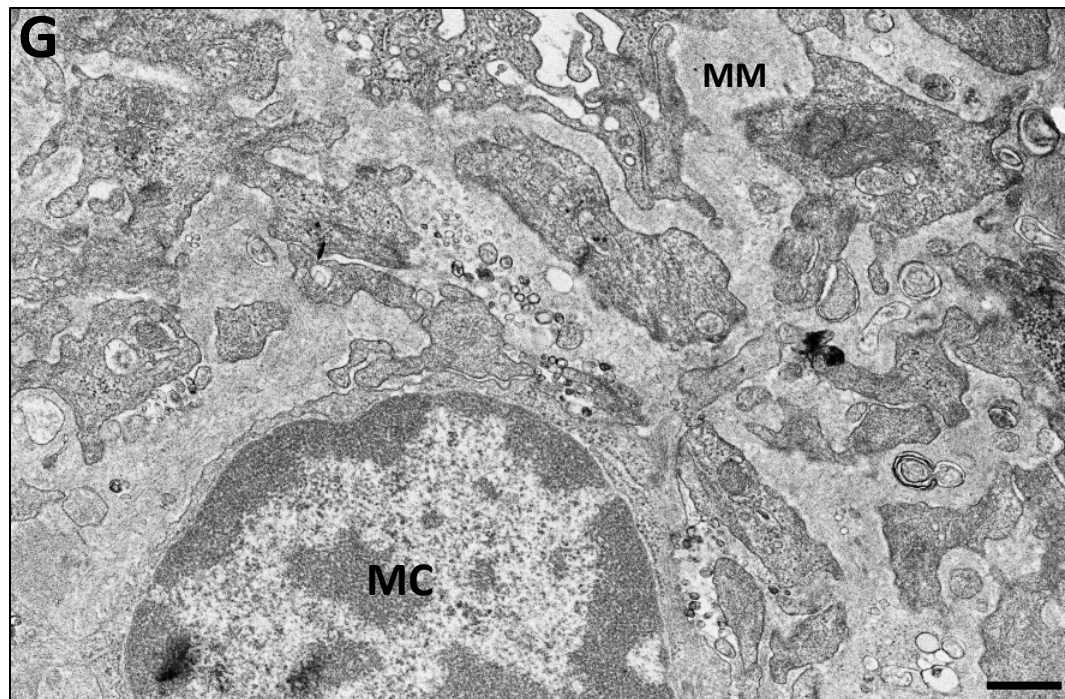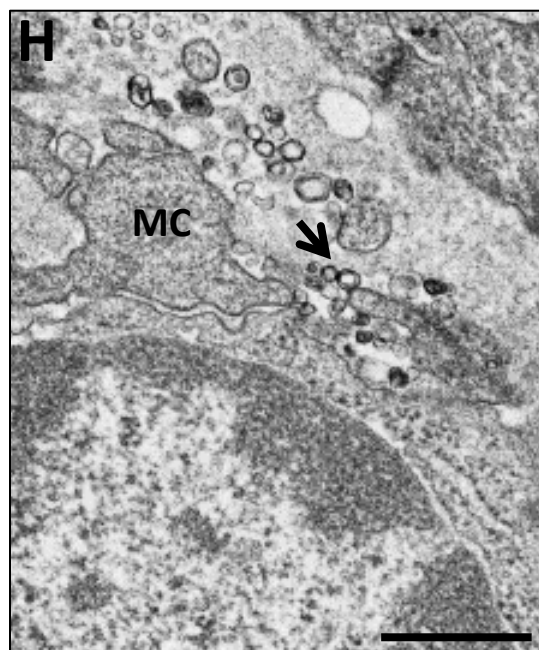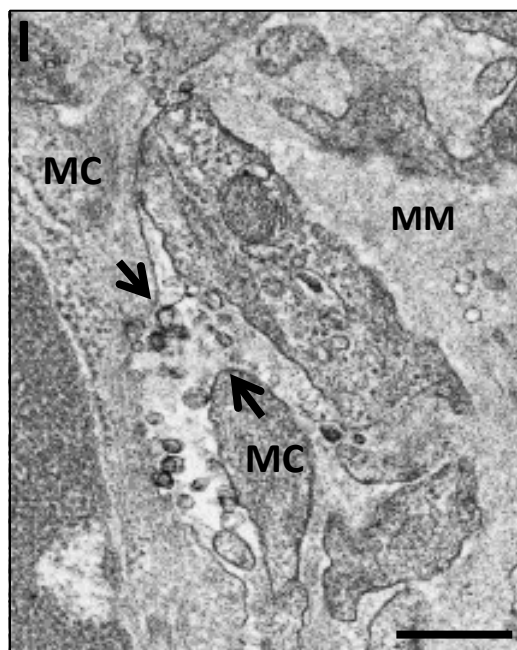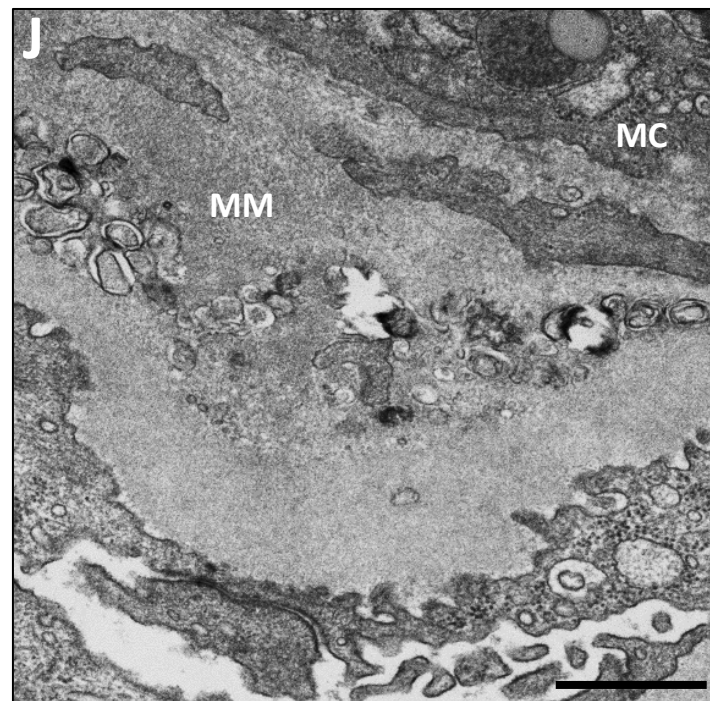

Supplement: S8 Fig — Gallery of EM images. (A) LpX accumulates in the mesangial matrix and is taken up by mesangial cells (MC) by macropinocytosis. LpX binds to MCs which extend lamellipodia (black arrows) entrapping matrical LpX. Regions enclosed by white boxes in (A) are enlarged in (A1, A2). Fat droplets within MCs are denoted by asterisks, and MC lamellipodia entrapping LpX particles are indicated by black arrows. (A, B) Very large macropinosomes in the process of formation are seen to enclose numerous LpX particles ((A2) large white arrow; (B)). In (C), a renal capillary EC near the mesangium contains a large phagosome enveloping a large LpX particle, and abundant deposition of LpX particles concomitant with matrix degradation is seen in the nearby mesangial matrix (MM). (D-G). Additional examples of LpX binding to the surface of MCs, MC lamellipodial extension,engulfment of matrical LpX and in (F), internalization and degradation of LpX in MC phagolysosomes. (H,I) Enlargements of regions in (G) showing LpX binding to MC lamellipodia. (J-N) Abundant deposition of LpX in MM. Scale bars: B = 100 nm; A2 = 200 nm; M = 300 nm; A, A1, C–N = 500 nm. (PDF) [file pone.0150083.s008.pdf]

# S9

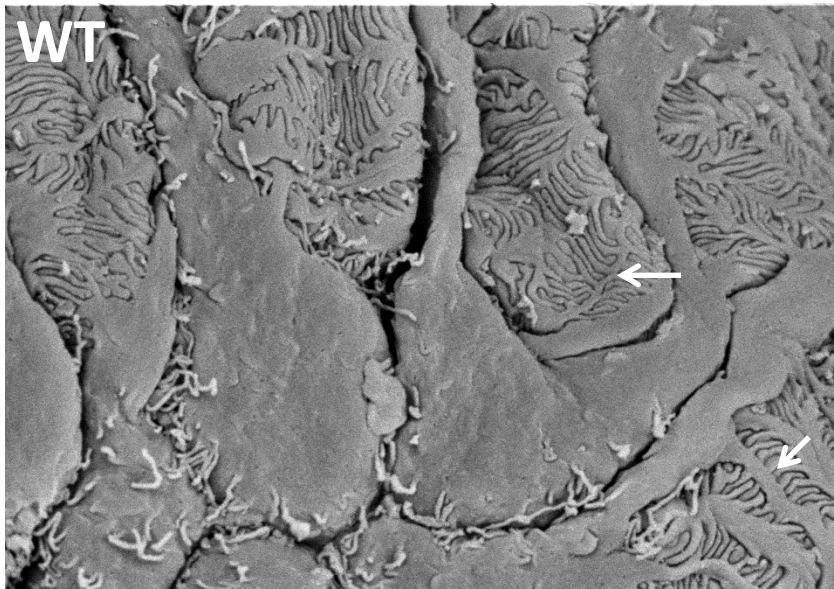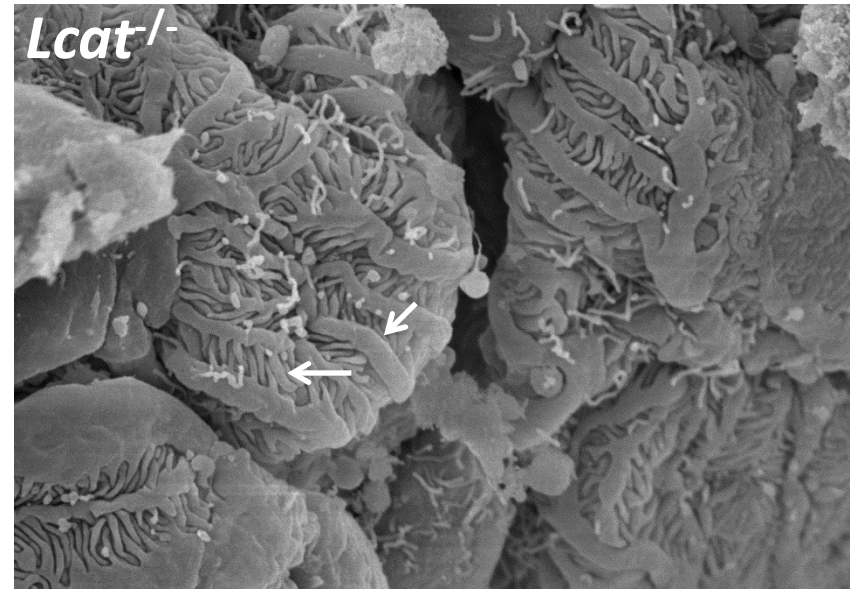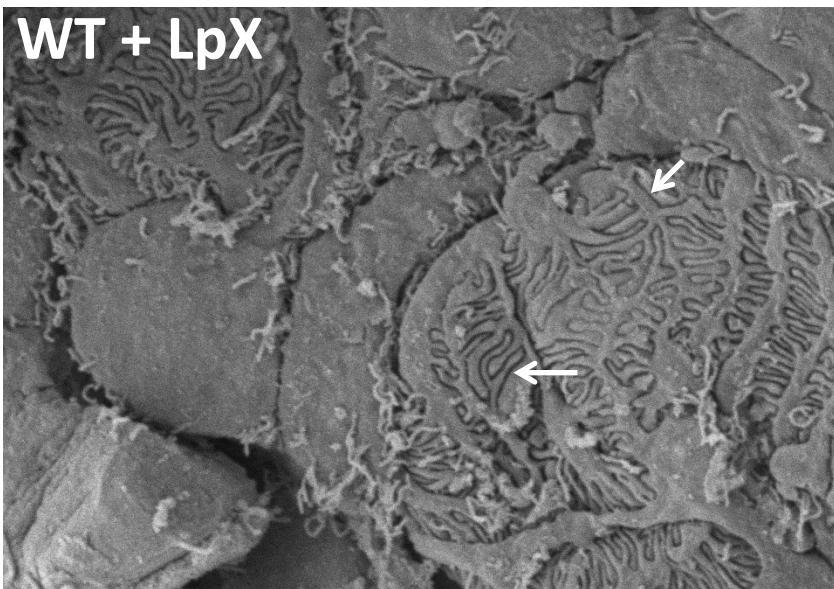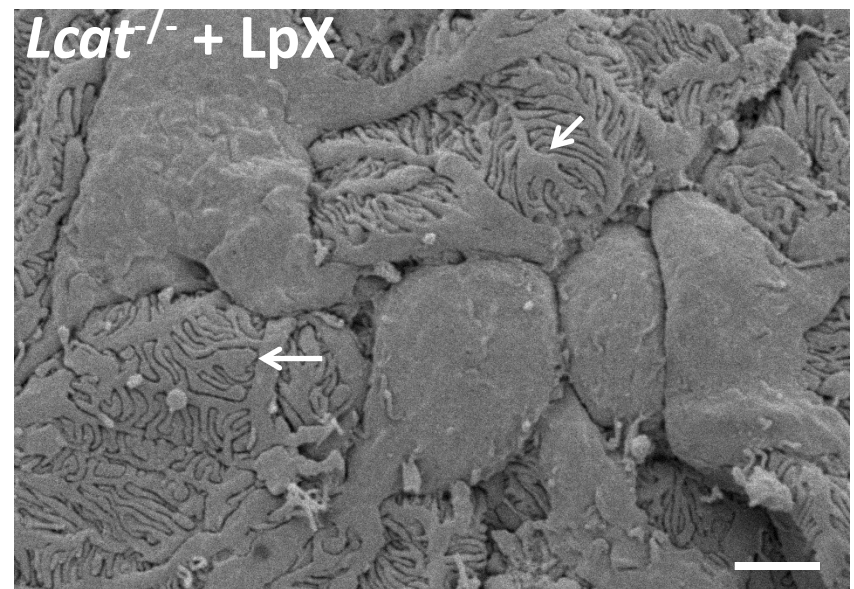

Supplement: S9 Fig — Representative SEM images of WT (left panels) and Lcat-/- (right panels) podocytes in the absence (upper panels) and presence (lower panels) of chronic LpX administration. Primary and secondary podocyte foot processes are indicated by small and large arrows, respectively. WT processes appear to be unaltered by LpX treatment (WT vs (WT + LpX)). Note the altered morphology of both primary and secondary podocyte processes in Lcat-/- glomeruli with or without LpX; (Lcat-/- + LpX) and (Lcat-/-—LpX), respectively. In Lcat-/- mice, several primary and secondary processes are bulged, exemplified by the small and large arrows, respectively; in Lcat-/- mice + LpX, note the bulged primary process (small arrow) and focal foot effacement (fused foot processes; large arrow). Scale bar = 20 μm. (PDF) [file pone.0150083.s009.pdf]
