# Supplementary material for: Lipoprotein X Causes Renal Disease in LCAT Deficiency
Source: PLoS One. 2016 Feb 26;11(2):e0150083. doi: 10.1371/journal.pone.0150083 (PMC4769176; doi:10.1371/journal.pone.0150083)
Supplement: S1 Table — The expression of a panel of genes known to be involved in the nephrotoxicity was analyzed in Lcat-/- mice either chronically-treated with LpX (n = 5) or with saline (n = 5). Differences between LpX- treated vs saline-treated Lcat-/- mice are reported in this table. Genes whose expressionare statistically different (Paired t-test) are highlighted. (DOCX) [file pone.0150083.s010.docx]

S1 Table: Kidney gene expression after LpX treatment in *Lcat-/-* mice

| **Gene** | **Fold Change**  **LpX injection vs saline** | **P Value** |
| --- | --- | --- |
| A2m | 1.06 | 0.709 |
| Aass | 1.41 | 0.186 |
| Abcb1a | -1.40 | 0.379 |
| Abcc2 | 1.51 | 0.060 |
| Aldh1a1 | 1.22 | 0.330 |
| Angptl4 | 1.06 | 0.670 |
| Anxa5 | 1.12 | 0.495 |
| Atf3 | 2.47 | 0.054 |
| Bhmt | -1.56 | 0.820 |
| Bmp1 | -1.03 | 0.949 |
| Bmp4 | -2.49 | 0.224 |
| **Btg2** | **1.51** | **0.044** |
| Calb1 | 1.07 | 0.942 |
| Cat | 1.42 | 0.149 |
| Ccl3 | 1.25 | 0.470 |
| Ccnd1 | -1.01 | 0.918 |
| Ccng1 | 1.27 | 0.182 |
| Ccs | 1.24 | 0.209 |
| Cd24a | 1.04 | 0.747 |
| Cd44 | 1.45 | 0.136 |
| Cdkn1a | 1.11 | 0.780 |
| Clu | 1.13 | 0.749 |
| Cp | -1.08 | 0.997 |
| Cst3 | 1.05 | 0.796 |
| Ctss | 1.26 | 0.262 |
| **Cxcl10** | **1.62** | **0.050** |
| Cxcl3 | -1.94 | 0.429 |
| Cyp2c54 | 1.65 | 0.188 |
| Cyp2d22 | -1.03 | 0.983 |
| Cyr61 | 2.51 | 0.064 |
| Egf | -1.04 | 0.801 |
| Fgb | 1.06 | 0.631 |
| Fmo2 | 1.36 | 0.095 |
| Fn1 | -1.03 | 0.964 |
| **G6pc** | **1.57** | **0.012** |
| G6pdx | 1.50 | 0.073 |
| Gadd45a | 1.75 | 0.039 |
| Gamt | 1.25 | 0.149 |
| Gatm | 1.41 | 0.095 |
| Gc | -1.41 | 0.864 |
| Ghr | 1.30 | 0.131 |
| Glul | -1.15 | 0.484 |
| Gpnmb | 1.08 | 0.730 |
| Gpx8 | 1.28 | 0.240 |
| Gstk1 | 1.25 | 0.126 |
| **Gstp1** | **1.39** | **0.039** |
| Havcr1 | 3.64 | 0.221 |
| Hmox1 | 2.06 | 0.149 |
| **Hmox2** | **1.48** | **0.009** |
| Hsp90aa1 | 1.32 | 0.329 |
| Idh1 | 1.34 | 0.243 |
| Igfbp1 | -1.15 | 0.848 |
| Igfbp3 | 1.20 | 0.136 |
| **Ipmk** | **1.38** | **0.010** |
| Klk1 | 1.06 | 0.706 |
| Lcn2 | 1.13 | 0.597 |
| Lgals3 | 1.21 | 0.182 |
| Mcm6 | 1.25 | 0.150 |
| Mgp | 1.25 | 0.286 |
| Mt1 | -1.23 | 0.056 |
| Nox4 | 1.10 | 0.473 |
| Nphs2 | 1.56 | 0.018 |
| **Nqo1** | **2.12** | **0.008** |
| Oat | -1.05 | 0.832 |
| Odc1 | 1.98 | 0.198 |
| **Rgn** | **1.70** | **0.019** |
| **Rtn4** | **1.45** | **0.024** |
| Scd1 | 1.44 | 0.414 |
| Slc22a1 | 1.36 | 0.026 |
| Slc22a5 | 1.17 | 0.272 |
| Slc22a6 | 2.27 | 0.174 |
| Socs3 | 1.25 | 0.487 |
| **Sod2** | **3.03** | **0.047** |
| **Sod3** | **2.61** | **0.014** |
| Spp1 | 1.75 | 0.178 |
| Sprr1a | 2.45 | 0.315 |
| Timp1 | 3.68 | 0.057 |
| **Tmsb10** | **2.42** | **0.008** |
| Tnfrsf12a | 2.12 | 0.057 |
| **Uchl1** | **5.80** | **0.035** |
| Ugt1a1 | 1.10 | 0.669 |
| **Ugt1a6a** | **3.35** | **0.008** |
| **Vcam1** | **2.81** | **0.046** |
| **Vim** | **2.68** | **0.029** |
